# Supplementary material for: Interventions to promote the health and well-being of children under 5s experiencing homelessness in high-income countries: a scoping review
Source: BMJ Open. 2024 Jan 12;14(1):e076492. doi: 10.1136/bmjopen-2023-076492 (PMC10806763; doi:10.1136/bmjopen-2023-076492)
Supplement: Supplementary data [file bmjopen-2023-076492supp001.pdf]

Scoping Review Protocol

TITLE

Interventions to promote the health and well- being of children under 5s experiencing homelessness in high-income countries: A Scoping Review

PROTOCOL INFORMATION

Authors: All team members
Contact person: Monica Lakhanpaul
Dates: Anticipated start date of search: December 2021
Anticipated completion date: March 2022

BACKGROUND

According to the McKinney-Vento definition of homeless, "homeless children and youths" means individuals who lack a fixed, regular, and adequate night-time residence (Miller, 2009). According to the UN Office of the High Commissioner for Human Rights, homelessness has "emerged as a worldwide human rights concern," especially in high-income nation-states with the means to address it. Between 2014 and 2018, family homelessness almost doubled in Ireland, rising from 407 to over 1,600 families. Between 2006 and 2013, New Zealand had a 44% rise in family homelessness. In 2018, the United States had around 56,300 families with children, accounting for one-third of the country's homeless population (Rosenthal DM, 2021). In 2019, the charity Shelter reported that a child loses their home every 8 min in Great Britain, which is the equivalent of 183 children per day. In 2019, the overall number of children who were homeless or in temporary housing in England climbed to 126020, including 88080 in London. According to the Children's Commissioner, there might be around 210000 homeless children in temporary accommodation or couch surfing in England, as well as roughly 585000 people who are either homeless or in danger of becoming homeless.

In particular, young children aged ≤5 years living in temporary accommodation (U5TA) have an inconspicuous plight. There is a lack of policy supporting them since they are not on the streets as homeless. However, many U5TA have pre-existing conditions such as epilepsy, asthma, anxiety, and diabetes; they might be the most susceptible to viral infection (Rosenthal et al., 2020). Moreover, the first five years of life is a critical duration for the optimal development of the brain, especially for children who experience poverty/housing/transient lifestyle, which not only leads to many health concerns but also leads to language development and motor skills and social problems (Cusick and Georgieff, 2016).

Therefore, this scoping review was undertaken to gather existing interventions, especially for families with homeless children within themes: feeding, nutrition, care practice, parenting, dental, wellbeing and mental health, safe sleeping, physical activity, parenting support, which were already effectively conducted in other HICs. Those themes are essential to the integrated health of U5TA, and there is a complex need to include all those themes to address or alleviate the current situation of homelessness. The primary objective of this review is to examine the content and method of delivery of interventions that are culturally sensitive and accessible for the demographic that is on the crossroads of poverty/housing/transient lifestyle. Specifically, our study also aims to address and answer the following question: How best to communicate with mobile populations with poor health literacy/literacy/language barriers? Who are possible and critical points of contact? How to develop an inclusive and engaging practice with

populations in homelessness? How to create appropriate, acceptable, and accessible communication materials? (e.g., through health visitors, family nurse partnerships, etc.)

The scoping review is a core work package of the project titled, “Children in Homeless Accommodations Managing Pandemic Invisibility or Non-inclusive Strategies” to map the existing global evidence on the content and method of delivery of practical strategies and interventions to improve coverage, access, and utilization of early child health programmes in marginalized and excluded under5s.

OBJECTIVES

- O-1. To identify the evidence base available around the content of delivery of interventions programmes for families with children under 5.
- O-2. To explore the method of delivery of these interventions: when to intervene, how to intervene, entry points for crucial health concerns.
- O-3. To find out the most feasible settings in which the strategies and interventions are effective

Specific parameters that will be mapped from studies that relate to the review objectives will comprise:

(i) Challenge area

(ii) Settings of interventions (Content and delivery method)

(iii) Outcome of the interventions

(iv) Culturally accessible/appropriate for mobile populations

(v) Scale of impact

(vi) Economy of impact

METHODS

We will conduct a targeted systematic rapid global scoping review (supplemented by knowledge and lived experiences from international experts) using the Arksey and O’Malley framework (Levac et al., 2010)

1.1. Review Methods
1.1.1. Inclusion Criteria
1.1.1.1. **Language and Geography:** English language studies of HICs;

1.1.1.2. **Types of studies:** Studies of any design, where the study objective was to describe, measure, or evaluate the piloting or implementation of a strategy, tactics, process, and/or method targeted at improving child health programmes in High-income countries;

1.1.1.3. **Types of participants:** Studies that include interventions for under-5 children; The review will look into two qualifiers – marginalized and excluded what? Populations?. Marginalization is defined as “a process whereby something or someone is pushed to the edge of a group and accorded lesser importance.” Social exclusion is defined as “ It involves the lack or denial of resources, rights, goods and services, and the inability to participate **in** the normal relationships and activities, available to the majority of people **in** a society, whether **in** economic, social, cultural or political

arenas." Operationally these two terminologies have been defined to be inclusive of the following keywords:

(Marginal[Title/Abstract])) OR (Marginalised[Title/Abstract])) OR (Marginalized[Title/Abstract])) OR (Refugee[Title/Abstract])) OR (Homeless[Title/Abstract])) OR (Migrant[Title/Abstract])) OR (financial catastrophe[Title/Abstract])) OR (poverty[Title/Abstract])) OR (conflict-affected[Title/Abstract])) OR (Under attack[Title/Abstract])) OR (Displaced[Title/Abstract])) OR (Temporary Accommodation[Title/Abstract])) OR (Temporary Settlement[Title/Abstract])) OR (Temporary Housing[Title/Abstract])) OR (Transitional Settlement[Title/Abstract])) OR (Transitional shelter[Title/Abstract])) OR (Emergency shelter[Title/Abstract])) OR (Emergency accommodation[Title/Abstract])) OR (Emergency housing[Title/Abstract])) OR (Makeshift shelter[Title/Abstract])) OR (Makeshift accommodation[Title/Abstract])) OR (Slash[Title/Abstract] AND Burn Cultivation[Title/Abstract])) OR (Shifting Cultivation[Title/Abstract])) OR (Feral[Title/Abstract])))

1.1.1.4. **Types of intervention:** Studies that will include interventions to improve or optimize the coverage of services, accessibility of the target population, and service utilization; we will include efficacy trials that include interventions only to improve specific health conditions within themes: feeding, nutrition, care practice, parenting, dental, wellbeing, and mental health, safe sleeping, physical activity, parenting support; inclusion of any bridging study will be decided jointly by the lead reviewers and the PI based on any added value)

1.1.1.5. **Types of comparators:** Studies that include marginality, conflict, or inequality for overall outcome, comparison, or sub-grouping.

1.1.1.6. **Types of outcome measures:** Studies that measure service output (coverage, access, utilization) and health (mortality, morbidity, quality-adjusted life years, disease/ disability-adjusted life years) and/or economic (efficiency, cost, return on investment);

1.1.1.7. **Time of Publications:** The dates searched will be from January 2000 to

FINAL SEARCH TERM (See Annexure for More Details)
((((((Child*[Title/Abstract]) OR (((Under five[Title/Abstract]) OR (Under 5[Title/Abstract])) OR (U5[Title/Abstract]))) OR (((Newborn[Title/Abstract]) OR (Infant[Title/Abstract])) OR (1000 days[Title/Abstract]))) AND (((Intervention*[Title/Abstract]) OR (Strateg*[Title/Abstract])) OR (Practice*[Title/Abstract]))) AND ((((((((((((((((((((((((Vulnerable[Title/Abstract]) OR (Marginal[Title/Abstract])) OR (Marginalised[Title/Abstract])) OR (Marginalized[Title/Abstract])) OR (Refugee[Title/Abstract])) OR (Homeless[Title/Abstract])) OR (Migrant[Title/Abstract])) OR (financial catastrophe[Title/Abstract])) OR (poverty[Title/Abstract])) OR (conflict-affected[Title/Abstract])) OR (Under attack[Title/Abstract])) OR (Displaced[Title/Abstract])) OR (Temporary Accommodation[Title/Abstract])) OR (Temporary Settlement[Title/Abstract])) OR (Temporary Housing[Title/Abstract])) OR (Transitional Settlement[Title/Abstract])) OR (Transitional shelter[Title/Abstract])) OR (Emergency shelter[Title/Abstract])) OR (Emergency accommodation[Title/Abstract])) OR (Emergency housing[Title/Abstract])) OR (Makeshift shelter[Title/Abstract])) OR (Makeshift accommodation[Title/Abstract])) OR (Slash[Title/Abstract] AND Burn Cultivation[Title/Abstract])) OR (Shifting Cultivation[Title/Abstract])) OR (Feral[Title/Abstract]))) AND (((((((Access[Title/Abstract]) OR (Accessibility[Title/Abstract])) OR (Inaccessible[Title/Abstract])) OR (inaccess[Title/Abstract])) OR (((coverage[Title/Abstract]) OR (outreach[Title/Abstract])) OR (reach[Title/Abstract]))) OR ((((Use[Title/Abstract]) OR (Usage[Title/Abstract])) OR (Utilisation[Title/Abstract])) OR (Utilization[Title/Abstract]))) OR ((Available[Title/Abstract]) OR (Availability[Title/Abstract])))) AND (((((((Feeding[Title/Abstract]) OR (Nutrition[Title/Abstract])) OR (Care practice[Title/Abstract])) OR (Parenting[Title/Abstract])) OR (((dental[Title/Abstract]) OR (wellbeing[Title/Abstract])) OR (mental health[Title/Abstract]))) OR ((((safe sleeping[Title/Abstract]) OR (physical activity[Title/Abstract])) OR (parenting support[Title/Abstract])) OR (sleep hygiene[Title/Abstract]))) OR ((sleep practice[Title/Abstract]))))) AND (Andorra*[Title/Abstract] OR Antigua and Barbuda*[Title/Abstract] OR Australia*[Title/Abstract] OR Austria*[Title/Abstract] OR Bahrain*[Title/Abstract] OR Barbados*[Title/Abstract] OR Belgium*[Title/Abstract] OR Brunei*[Title/Abstract] OR Canada*[Title/Abstract] OR Chile*[Title/Abstract] OR Croatia*[Title/Abstract] OR Cyprus*[Title/Abstract] OR Czech Republic*[Title/Abstract] OR Denmark*[Title/Abstract] OR Estonia*[Title/Abstract] OR Finland*[Title/Abstract] OR France*[Title/Abstract] OR Germany*[Title/Abstract] OR Greece*[Title/Abstract] OR Hungary*[Title/Abstract] OR Iceland*[Title/Abstract] OR Israel*[Title/Abstract] OR Italy*[Title/Abstract] OR Japan*[Title/Abstract] OR South Korea*[Title/Abstract] OR Kuwait*[Title/Abstract] OR Latvia*[Title/Abstract] OR Liechtenstein*[Title/Abstract] OR Lithuania*[Title/Abstract] OR Luxembourg*[Title/Abstract] OR Monaco*[Title/Abstract] OR Nauru*[Title/Abstract] OR Netherlands*[Title/Abstract] OR New Zealand*[Title/Abstract] OR Norway*[Title/Abstract] OR Oman*[Title/Abstract] OR Palau*[Title/Abstract] OR Poland*[Title/Abstract] OR Portugal*[Title/Abstract] OR Qatar*[Title/Abstract] OR Saint Kitts and Nevis*[Title/Abstract] OR San Marino*[Title/Abstract] OR Seychelles*[Title/Abstract] OR Singapore*[Title/Abstract] OR Slovakia*[Title/Abstract] OR Slovenia*[Title/Abstract] OR Spain*[Title/Abstract] OR Sweden*[Title/Abstract] OR Switzerland*[Title/Abstract] OR Trinidad and Tobago*[Title/Abstract] OR United Arab Emirates*[Title/Abstract] OR United States*[Title/Abstract] OR Aruba*[Title/Abstract] OR Bermuda*[Title/Abstract] OR British Virgin Islands*[Title/Abstract] OR Cayman Islands*[Title/Abstract] OR Cook Islands*[Title/Abstract] OR Curaçao*[Title/Abstract] OR Faroe Islands*[Title/Abstract] OR French Polynesia*[Title/Abstract] OR Gibraltar*[Title/Abstract] OR Greenland*[Title/Abstract] OR Guam*[Title/Abstract] OR Hong Kong*[Title/Abstract] OR Isle of Man*[Title/Abstract] OR New Caledonia*[Title/Abstract] OR Northern Mariana Islands*[Title/Abstract] OR Puerto Rico*[Title/Abstract] OR Saint Martin*[Title/Abstract] OR Taiwan*[Title/Abstract] OR Turks and Caicos Islands*[Title/Abstract] OR U.S. Virgin Islands*[Title/Abstract])AND (("2000/01/01"[Date - Publication] : "3000"[Date - Publication]))

December 2021.

1.1.2. Search Database

1.1.2.1. **Electronic Database:**

i. Databases: PubMed, MEDLINE, SCOPUS, The Cochrane Library (Cochrane Database of Systematic Reviews, Cochrane Central Register of Controlled Trials [CENTRAL]), and Google Scholar.

ii. Clinical trial registries: ClinicalTrials.gov, Current Controlled Trials meta Register of Controlled Trials, International Clinical Trials Registry Platform (ICTRP) and.

1.1.2.2. **Grey literature:** Conference abstracts, Third Sector Reports.

1.1.2.3. **Correspondence:** Experts from Donor/ Philanthropic organizations and other key experts selected for an interview will be contacted for any additional published or unpublished work.

1.1.3. Relational and Citation Search:

We will conduct snowballing to screen cited articles of the 1st iteration of selections, followed by citations and related articles for each inclusion in PubMed, Google Scholar, and PubMed respectively. A maximum of three iterations will be undertaken.

1.1.4. DATA COLLECTION

1.1.4.1. **Screening Tool and Selection Method:** All articles will be extracted and compiled in a single spreadsheet. The spreadsheet will be equipped with duplicate study filters, and inclusion filters (inclusion criteria not managed through Search Term)

**Screening Filters**
**Geography:** High income countries (HICs) (Inclusion)<br>**Types of Emergency:** Grade 1, Grade 2, Grade 3 emergencies (WHO),<br>**Age of Target Population:** 0-1 (inclusion), 1-5 (inclusion), 5-14 (Exclusion)<br>**Interventions:** Interventions on access, coverage, utilization of health services; (inclusion)<br>**Measurements:** Output, outcome, impact (inclusion) input, process (exclusion)

and selection columns. Inclusion filters will be filled in independently by one reviewer (YT). Selection filters will be applied independently by two reviewers (YT and KS). All studies selected by two reviewers will be included. All studies rejected by two reviewers will be rejected. All studies having one inclusion will be marked as disputed selection. In case of dispute in selection third expert reviewer (ML) will make the final inclusion decision.

1.1.4.2. **Rounds of Screening:** Two round of screening will be undertaken– Title-Abstract (TiAb) and Full Text (FT).

1.1.4.3. **Preferred Reporting Items for Systematic Reviews and Meta-Analyses (PRISMA) Compliance:** The reasons for exclusion will be recorded and mapped in a flow chart as per PRISMA guidelines.

1.1.4.4. **Data Extraction:** A data extraction form (DEF) will be developed as per CEB Critically Appraised Topics (CAT) guidelines by KS and piloted by two authors (YT and KS) on the first 6 article inclusions (3 each). The piloting will essentially inform whether the DEF can extract necessary and sufficient information as per the objectives set. Any queries raised by the pilot will be reviewed by ML, NS, and ZP. The form will be amended based on the pilot. Two reviewers (YT and KS) will use the finalized form to extract data from full-text articles. If clarification is required, a domain expert (NS, YP, ML) will be consulted.

1.1.5. **Assessment of Risk of Bias (RoB):** RoB assessment will only be used for interventional studies for which results will be used for quantitative analysis or pooling. We will use multiple tools for risk of bias assessment based on the study type–

i. Systematic Reviews: **GRADE** for bias due to selective publication and selective non-reporting for systematic reviews

ii. Randomized Trials: **Cochrane RoB 2.0** to assess multiple sources of bias

iii. Non-randomised studies of interventions: **RoBANS** for bias due to selective non-reporting and bias in the selection of the reported result.

1.1.6. **Critical Appraisal for the included studies:** All studies will be critically appraised as per the CAT grading.

1.2. **Strategy for data synthesis**

The set of evidence will be categorized into qualitative, quantitative and mixed method. The qualitative evidence will be coded to find qualifiers through Word Cloud. The quantitative evidence will be subjected to descriptive analysis, followed by meta-analysis if possible.

1.3. **Referencing Software:** ENDNOTE

1.4. **Quality Assessment**

https://www.who.int/hrh/retention/annex1_grade_evidence_profiles.pdf

1.5. **Method Schema**

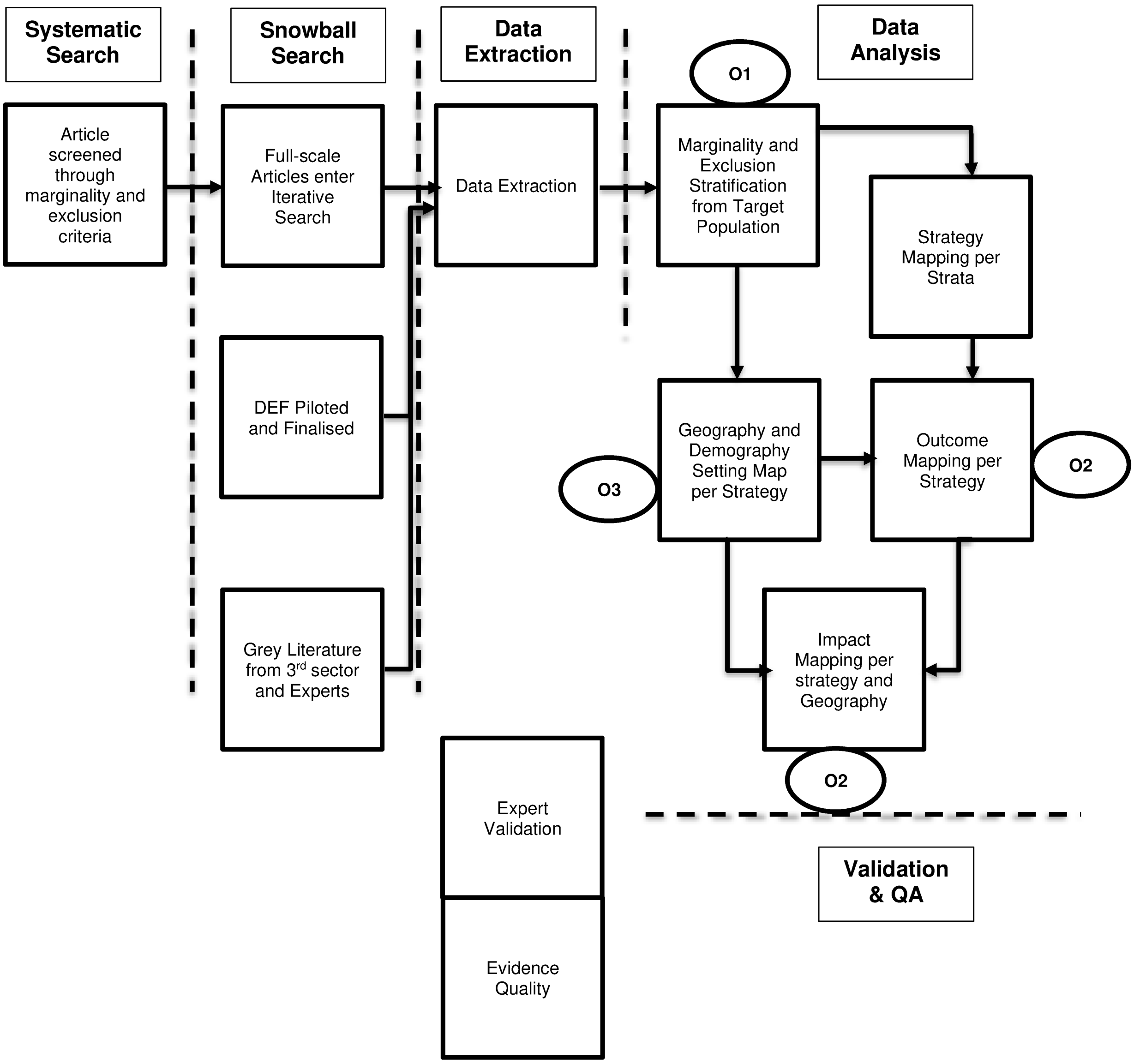

ANNEXURE

Search Strategy

Search number	Query	Sort By	Filters	Search Details	Results	Time
18	(("2000/01/01"[Date - Publication] : "3000/12/31"[Date - Publication])) AND ((((((("child*"[Title/Abstract]) OR ("under five"[Title/Abstract] OR "under 5"[Title/Abstract] OR "U5"[Title/Abstract])) OR ("Newborn"[Title/Abstract] OR "Infant"[Title/Abstract] OR "1000 days"[Title/Abstract])) AND ("intervention*"[Title/Abstract] OR "strategy*"[Title/Abstract] OR "practice*"[Title/Abstract])) AND ("Vulnerable"[Title/Abstract] OR "Marginal"[Title/Abstract] OR "Marginalised"[Title/Abstract] OR "Marginalized"[Title/Abstract] OR "Refugee"[Title/Abstract] OR "Homeless"[Title/Abstract] OR "Migrant"[Title/Abstract] OR "financial catastrophe"[Title/Abstract] OR "poverty"[Title/Abstract] OR "conflict-affected"[Title/Abstract] OR ("Under"[All Fields] OR "attack"[Title/Abstract]) OR "Displaced"[Title/Abstract] OR "temporary accommodation"[Title/Abstract] OR "temporary settlement"[Title/Abstract] OR "temporary housing"[Title/Abstract] OR (("transit"[All Fields] OR "transited"[All Fields] OR "transiting"[All Fields] OR "transition"[All Fields] OR "Transitional"[All Fields] OR "transitionals"[All Fields] OR "transitioned"[All Fields] OR "transitioning"[All Fields] OR "transitions"[All Fields] OR "transits"[All Fields]) OR "Settlement"[Title/Abstract]) OR "transitional shelter"[Title/Abstract] OR "emergency shelter"[Title/Abstract] OR "emergency accommodation"[Title/Abstract] OR "emergency housing"[Title/Abstract] OR ("Makeshift"[All Fields] OR "shelter"[Title/Abstract]) OR ("Makeshift"[All Fields] OR "Accommodation"[Title/Abstract]) OR ("Slash"[Title/Abstract] OR "burn cultivation"[Title/Abstract]) OR "shifting cultivation"[Title/Abstract] OR "Feral"[Title/Abstract)) AND (((("Access"[Title/Abstract] OR "Accessibility"[Title/Abstract] OR "Inaccessible"[Title/Abstract] OR "inaccess"[Title/Abstract]) OR ("coverage"[Title/Abstract] OR "outreach"[Title/Abstract] OR "reach"[Title/Abstract])) OR ("Use"[Title/Abstract] OR "Usage"[Title/Abstract] OR "Utilisation"[Title/Abstract] OR "Utilization"[Title/Abstract])) OR ("Available"[Title/Abstract] OR			("child*"[Title/Abstract] OR ("under five"[Title/Abstract] OR "under 5"[Title/Abstract] OR "U5"[Title/Abstract]) OR ("Newborn"[Title/Abstract] OR "Infant"[Title/Abstract] OR "1000 days"[Title/Abstract])) AND ("intervention*"[Title/Abstract] OR "strategy*"[Title/Abstract] OR "practice*"[Title/Abstract]) AND ("Vulnerable"[Title/Abstract] OR "Marginal"[Title/Abstract] OR "Marginalised"[Title/Abstract] OR "Marginalized"[Title/Abstract] OR "Refugee"[Title/Abstract] OR "Homeless"[Title/Abstract] OR "Migrant"[Title/Abstract] OR "financial catastrophe"[Title/Abstract] OR "poverty"[Title/Abstract] OR "conflict-affected"[Title/Abstract] OR ("Under"[All Fields] AND "attack"[Title/Abstract]) OR "Displaced"[Title/Abstract] OR "temporary accommodation"[Title/Abstract] OR "temporary settlement"[Title/Abstract] OR "temporary housing"[Title/Abstract] OR (("transit"[All Fields] OR "transited"[All Fields] OR "transiting"[All Fields] OR "transition"[All Fields] OR "Transitional"[All Fields] OR "transitionals"[All Fields] OR "transitioned"[All Fields] OR "transitioning"[All Fields] OR "transitions"[All Fields] OR "transits"[All Fields]) AND "Settlement"[Title/Abstract]) OR "transitional shelter"[Title/Abstract] OR "emergency shelter"[Title/Abstract] OR "emergency accommodation"[Title/Abstract] OR "emergency housing"[Title/Abstract] OR ("Makeshift"[All Fields] AND "shelter"[Title/Abstract]) OR ("Makeshift"[All Fields]	523	2:33:18

	"Availability"[Title/Abstract]))) AND ((Feeding*[Title/Abstract]) OR (nutrition*[Title/Abstract]) OR (care practice*[Title/Abstract]) OR (parenting*[Title/Abstract]) OR (dental*[Title/Abstract]) OR (wellbeing*[Title/Abstract]) OR (mental health*[Title/Abstract]) OR (safe sleeping*[Title/Abstract]) OR (physical activity*[Title/Abstract]) OR (parenting support*[Title/Abstract]) OR (sleep hygiene*[Title/Abstract]) OR (physical activity*[Title/Abstract]))) AND (Andorra*[Title/Abstract] OR Antigua and Barbuda*[Title/Abstract] OR Australia*[Title/Abstract] OR Austria*[Title/Abstract] OR Bahrain*[Title/Abstract] OR Barbados*[Title/Abstract] OR Belgium*[Title/Abstract] OR Brunei*[Title/Abstract] OR Canada*[Title/Abstract] OR Chile*[Title/Abstract] OR Croatia*[Title/Abstract] OR Cyprus*[Title/Abstract] OR Czech Republic*[Title/Abstract] OR Denmark*[Title/Abstract] OR Estonia*[Title/Abstract] OR Finland*[Title/Abstract] OR France*[Title/Abstract] OR Germany*[Title/Abstract] OR Greece*[Title/Abstract] OR Hungary*[Title/Abstract] OR Iceland*[Title/Abstract] OR Israel*[Title/Abstract] OR Italy*[Title/Abstract] OR Japan*[Title/Abstract] OR South Korea*[Title/Abstract] OR Kuwait*[Title/Abstract] OR Latvia*[Title/Abstract] OR Liechtenstein*[Title/Abstract] OR Lithuania*[Title/Abstract] OR Luxembourg*[Title/Abstract] OR Monaco*[Title/Abstract] OR Nauru*[Title/Abstract] OR Netherlands*[Title/Abstract] OR New Zealand*[Title/Abstract] OR Norway*[Title/Abstract] OR Oman*[Title/Abstract] OR Palau*[Title/Abstract] OR Poland*[Title/Abstract] OR Portugal*[Title/Abstract] OR Qatar*[Title/Abstract] OR Saint Kitts and Nevis*[Title/Abstract] OR San Marino*[Title/Abstract] OR Seychelles*[Title/Abstract] OR Singapore*[Title/Abstract] OR Slovakia*[Title/Abstract] OR Slovenia*[Title/Abstract] OR Spain*[Title/Abstract] OR Sweden*[Title/Abstract] OR Switzerland*[Title/Abstract] OR Trinidad and Tobago*[Title/Abstract] OR United Arab Emirates*[Title/Abstract] OR United States*[Title/Abstract] OR Aruba*[Title/Abstract] OR Bermuda*[Title/Abstract] OR British Virgin Islands*[Title/Abstract] OR Cayman Islands*[Title/Abstract] OR Cook Islands*[Title/Abstract] OR Curaçao*[Title/Abstract] OR Faroe Islands*[Title/Abstract] OR French Polynesia*[Title/Abstract] OR			AND "Accommodation"[Title/Abstract]) OR ("Slash"[Title/Abstract] AND "burn cultivation"[Title/Abstract]) OR "shifting cultivation"[Title/Abstract] OR "Feral"[Title/Abstract]) AND ("Access"[Title/Abstract] OR "Accessibility"[Title/Abstract] OR "Inaccessible"[Title/Abstract] OR "inaccess"[Title/Abstract] OR ("coverage"[Title/Abstract] OR "outreach"[Title/Abstract] OR "reach"[Title/Abstract]) OR ("Use"[Title/Abstract] OR "Usage"[Title/Abstract] OR "Utilisation"[Title/Abstract] OR "Utilization"[Title/Abstract]) OR ("Available"[Title/Abstract] OR "Availability"[Title/Abstract])) AND (Feeding*[Title/Abstract])) OR (nutrition*[Title/Abstract])) OR (care practice*[Title/Abstract])) OR (parenting*[Title/Abstract])) OR (dental*[Title/Abstract])) OR (wellbeing*[Title/Abstract])) OR (mental health*[Title/Abstract])) OR (safe sleeping*[Title/Abstract])) OR (physical activity*[Title/Abstract])) OR (parenting support*[Title/Abstract])) OR (sleep hygiene*[Title/Abstract])) OR (physical activity*[Title/Abstract])) AND (Andorra*[Title/Abstract] OR Antigua and Barbuda*[Title/Abstract] OR Australia*[Title/Abstract] OR Austria*[Title/Abstract] OR Bahrain*[Title/Abstract] OR Barbados*[Title/Abstract] OR Belgium*[Title/Abstract] OR Brunei*[Title/Abstract] OR Canada*[Title/Abstract] OR Chile*[Title/Abstract] OR Croatia*[Title/Abstract] OR Cyprus*[Title/Abstract] OR Czech Republic*[Title/Abstract] OR		

	Gibraltar*[Title/Abstract] OR Greenland*[Title/Abstract] OR Guam*[Title/Abstract] OR Hong Kong*[Title/Abstract] OR Isle of Man*[Title/Abstract] OR New Caledonia*[Title/Abstract] OR Northern Mariana Islands*[Title/Abstract] OR Puerto Rico*[Title/Abstract] OR Saint Martin*[Title/Abstract] OR Taiwan*[Title/Abstract] OR Turks and Caicos Islands*[Title/Abstract] OR U.S. Virgin Islands*[Title/Abstract]))			Denmark*[Title/Abstract] OR Estonia*[Title/Abstract] OR Finland*[Title/Abstract] OR France*[Title/Abstract] OR Germany*[Title/Abstract] OR Greece*[Title/Abstract] OR Hungary*[Title/Abstract] OR Iceland*[Title/Abstract] OR Israel*[Title/Abstract] OR Italy*[Title/Abstract] OR Japan*[Title/Abstract] OR South Korea*[Title/Abstract] OR Kuwait*[Title/Abstract] OR Latvia*[Title/Abstract] OR Liechtenstein*[Title/Abstract] OR Lithuania*[Title/Abstract] OR Luxembourg*[Title/Abstract] OR Monaco*[Title/Abstract] OR Nauru*[Title/Abstract] OR Netherlands*[Title/Abstract] OR New Zealand*[Title/Abstract] OR Norway*[Title/Abstract] OR Oman*[Title/Abstract] OR Palau*[Title/Abstract] OR Poland*[Title/Abstract] OR Portugal*[Title/Abstract] OR Qatar*[Title/Abstract] OR Saint Kitts and Nevis*[Title/Abstract] OR San Marino*[Title/Abstract] OR Seychelles*[Title/Abstract] OR Singapore*[Title/Abstract] OR Slovakia*[Title/Abstract] OR Slovenia*[Title/Abstract] OR Spain*[Title/Abstract] OR Sweden*[Title/Abstract] OR Switzerland*[Title/Abstract] OR Trinidad and Tobago*[Title/Abstract] OR United Arab Emirates*[Title/Abstract] OR United States*[Title/Abstract] OR Aruba*[Title/Abstract] OR Bermuda*[Title/Abstract] OR British Virgin Islands*[Title/Abstract] OR Cayman Islands*[Title/Abstract] OR Cook Islands*[Title/Abstract] OR Curaçao*[Title/Abstract] OR Faroe Islands*[Title/Abstract] OR French Polynesia*[Title/Abstract] OR Gibraltar*[Title/Abstract] OR Greenland*[Title/Abstract] OR Guam*[Title/Abstract] OR Hong Kong*[Title/Abstract] OR		

				Isle of Man*[Title/Abstract] OR New Caledonia*[Title/Abstract] OR Northern Mariana Islands*[Title/Abstract] OR Puerto Rico*[Title/Abstract] OR Saint Martin*[Title/Abstract] OR Taiwan*[Title/Abstract] OR Turks and Caicos Islands*[Title/Abstract] OR U.S. Virgin Islands*[Title/Abstract]))AND 2010/01/01:3000/12/31[Date - Publication]		

17	((((((("child*"[Title/Abstract]) OR ("under five"[Title/Abstract] OR "under 5"[Title/Abstract] OR "U5"[Title/Abstract])) OR ("Newborn"[Title/Abstract] OR "Infant"[Title/Abstract] OR "1000 days"[Title/Abstract])) AND ("intervention*"[Title/Abstract] OR "strategy*"[Title/Abstract] OR "practice*"[Title/Abstract])) AND ("Vulnerable"[Title/Abstract] OR "Marginal"[Title/Abstract] OR "Marginalised"[Title/Abstract] OR "Marginalized"[Title/Abstract] OR "Refugee"[Title/Abstract] OR "Homeless"[Title/Abstract] OR "Migrant"[Title/Abstract] OR "financial catastrophe"[Title/Abstract] OR "poverty"[Title/Abstract] OR "conflict-affected"[Title/Abstract] OR ("Under"[All Fields] OR "attack"[Title/Abstract]) OR "Displaced"[Title/Abstract] OR "temporary accommodation"[Title/Abstract] OR "temporary settlement"[Title/Abstract] OR "temporary housing"[Title/Abstract] OR (("transit"[All Fields] OR "transited"[All Fields] OR "transiting"[All Fields] OR "transition"[All Fields] OR "Transitional"[All Fields] OR "transitionals"[All Fields] OR "transitioned"[All Fields] OR "transitioning"[All Fields] OR "transitions"[All Fields] OR "transits"[All Fields]) OR "Settlement"[Title/Abstract]) OR "transitional shelter"[Title/Abstract] OR "emergency shelter"[Title/Abstract] OR "emergency accommodation"[Title/Abstract] OR "emergency housing"[Title/Abstract] OR ("Makeshift"[All Fields] OR "shelter"[Title/Abstract]) OR ("Makeshift"[All Fields] OR "Accommodation"[Title/Abstract]) OR ("Slash"[Title/Abstract] OR "burn cultivation"[Title/Abstract]) OR "shifting cultivation"[Title/Abstract] OR "Feral"[Title/Abstract])) AND (((("Access"[Title/Abstract] OR "Accessibility"[Title/Abstract] OR "Inaccessible"[Title/Abstract] OR "inaccess"[Title/Abstract]) OR ("coverage"[Title/Abstract] OR "outreach"[Title/Abstract] OR "reach"[Title/Abstract])) OR ("Use"[Title/Abstract] OR "Usage"[Title/Abstract] OR "Utilisation"[Title/Abstract] OR "Utilization"[Title/Abstract])) OR ("Available"[Title/Abstract] OR "Availability"[Title/Abstract]))) AND ((Feeding*[Title/Abstract]) OR (nutrition*[Title/Abstract]) OR (care practice*[Title/Abstract]) OR (parenting*[Title/Abstract]) OR (dental*[Title/Abstract]) OR (wellbeing*[Title/Abstract]) OR (mental health*[Title/Abstract]) OR (safe sleeping*[Title/Abstract]) OR (physical activity*[Title/Abstract]) OR			(("child*"[Title/Abstract] OR ("under five"[Title/Abstract] OR "under 5"[Title/Abstract] OR "U5"[Title/Abstract]) OR ("Newborn"[Title/Abstract] OR "Infant"[Title/Abstract] OR "1000 days"[Title/Abstract])) AND ("intervention*"[Title/Abstract] OR "strategy*"[Title/Abstract] OR "practice*"[Title/Abstract]) AND ("Vulnerable"[Title/Abstract] OR "Marginal"[Title/Abstract] OR "Marginalised"[Title/Abstract] OR "Marginalized"[Title/Abstract] OR "Refugee"[Title/Abstract] OR "Homeless"[Title/Abstract] OR "Migrant"[Title/Abstract] OR "financial catastrophe"[Title/Abstract] OR "poverty"[Title/Abstract] OR "conflict-affected"[Title/Abstract] OR ("Under"[All Fields] AND "attack"[Title/Abstract]) OR "Displaced"[Title/Abstract] OR "temporary accommodation"[Title/Abstract] OR "temporary settlement"[Title/Abstract] OR "temporary housing"[Title/Abstract] OR (("transit"[All Fields] OR "transited"[All Fields] OR "transiting"[All Fields] OR "transition"[All Fields] OR "Transitional"[All Fields] OR "transitionals"[All Fields] OR "transitioned"[All Fields] OR "transitioning"[All Fields] OR "transitions"[All Fields] OR "transits"[All Fields]) AND "Settlement"[Title/Abstract]) OR "transitional shelter"[Title/Abstract] OR "emergency shelter"[Title/Abstract] OR "emergency accommodation"[Title/Abstract] OR "emergency housing"[Title/Abstract] OR ("Makeshift"[All Fields] AND "shelter"[Title/Abstract]) OR ("Makeshift"[All Fields] AND "Accommodation"[Title/Abstract]) OR ("Slash"[Title/Abstract] AND "burn cultivation"[Title/Abstract]) OR "shifting cultivation"[Title/Abstract]	642	2:32:48

	(parenting support*[Title/Abstract]) OR (sleep hygiene*[Title/Abstract]) OR (physical activity*[Title/Abstract]))) AND (Andorra*[Title/Abstract] OR Antigua and Barbuda*[Title/Abstract] OR Australia*[Title/Abstract] OR Austria*[Title/Abstract] OR Bahrain*[Title/Abstract] OR Barbados*[Title/Abstract] OR Belgium*[Title/Abstract] OR Brunei*[Title/Abstract] OR Canada*[Title/Abstract] OR Chile*[Title/Abstract] OR Croatia*[Title/Abstract] OR Cyprus*[Title/Abstract] OR Czech Republic*[Title/Abstract] OR Denmark*[Title/Abstract] OR Estonia*[Title/Abstract] OR Finland*[Title/Abstract] OR France*[Title/Abstract] OR Germany*[Title/Abstract] OR Greece*[Title/Abstract] OR Hungary*[Title/Abstract] OR Iceland*[Title/Abstract] OR Israel*[Title/Abstract] OR Italy*[Title/Abstract] OR Japan*[Title/Abstract] OR South Korea*[Title/Abstract] OR Kuwait*[Title/Abstract] OR Latvia*[Title/Abstract] OR Liechtenstein*[Title/Abstract] OR Lithuania*[Title/Abstract] OR Luxembourg*[Title/Abstract] OR Monaco*[Title/Abstract] OR Nauru*[Title/Abstract] OR Netherlands*[Title/Abstract] OR New Zealand*[Title/Abstract] OR Norway*[Title/Abstract] OR Oman*[Title/Abstract] OR Palau*[Title/Abstract] OR Poland*[Title/Abstract] OR Portugal*[Title/Abstract] OR Qatar*[Title/Abstract] OR Saint Kitts and Nevis*[Title/Abstract] OR San Marino*[Title/Abstract] OR Seychelles*[Title/Abstract] OR Singapore*[Title/Abstract] OR Slovakia*[Title/Abstract] OR Slovenia*[Title/Abstract] OR Spain*[Title/Abstract] OR Sweden*[Title/Abstract] OR Switzerland*[Title/Abstract] OR Trinidad and Tobago*[Title/Abstract] OR United Arab Emirates*[Title/Abstract] OR United States*[Title/Abstract] OR Aruba*[Title/Abstract] OR Bermuda*[Title/Abstract] OR British Virgin Islands*[Title/Abstract] OR Cayman Islands*[Title/Abstract] OR Cook Islands*[Title/Abstract] OR Curaçao*[Title/Abstract] OR Faroe Islands*[Title/Abstract] OR French Polynesia*[Title/Abstract] OR Gibraltar*[Title/Abstract] OR Greenland*[Title/Abstract] OR Guam*[Title/Abstract] OR Hong Kong*[Title/Abstract] OR Isle of Man*[Title/Abstract] OR New Caledonia*[Title/Abstract] OR Northern Mariana Islands*[Title/Abstract] OR Puerto Rico*[Title/Abstract] OR Saint Martin*[Title/Abstract] OR			OR "Feral"[Title/Abstract]) AND ("Access"[Title/Abstract] OR "Accessibility"[Title/Abstract] OR "Inaccessible"[Title/Abstract] OR "inaccess"[Title/Abstract] OR ("coverage"[Title/Abstract] OR "outreach"[Title/Abstract] OR "reach"[Title/Abstract]) OR ("Use"[Title/Abstract] OR "Usage"[Title/Abstract] OR "Utilisation"[Title/Abstract] OR "Utilization"[Title/Abstract]) OR ("Available"[Title/Abstract] OR "Availability"[Title/Abstract]))) AND (Feeding*[Title/Abstract])) OR (nutrition*[Title/Abstract])) OR (care practice*[Title/Abstract])) OR (parenting*[Title/Abstract])) OR (dental*[Title/Abstract])) OR (wellbeing*[Title/Abstract])) OR (mental health*[Title/Abstract])) OR (safe sleeping*[Title/Abstract])) OR (physical activity*[Title/Abstract])) OR (parenting support*[Title/Abstract])) OR (sleep hygiene*[Title/Abstract])) OR (physical activity*[Title/Abstract]) AND (Andorra*[Title/Abstract] OR Antigua and Barbuda*[Title/Abstract] OR Australia*[Title/Abstract] OR Austria*[Title/Abstract] OR Bahrain*[Title/Abstract] OR Barbados*[Title/Abstract] OR Belgium*[Title/Abstract] OR Brunei*[Title/Abstract] OR Canada*[Title/Abstract] OR Chile*[Title/Abstract] OR Croatia*[Title/Abstract] OR Cyprus*[Title/Abstract] OR Czech Republic*[Title/Abstract] OR Denmark*[Title/Abstract] OR Estonia*[Title/Abstract] OR Finland*[Title/Abstract] OR France*[Title/Abstract] OR Germany*[Title/Abstract] OR Greece*[Title/Abstract] OR		

	Taiwan*[Title/Abstract] OR Turks and Caicos Islands*[Title/Abstract] OR U.S. Virgin Islands*[Title/Abstract])			Hungary*[Title/Abstract] OR Iceland*[Title/Abstract] OR Israel*[Title/Abstract] OR Italy*[Title/Abstract] OR Japan*[Title/Abstract] OR South Korea*[Title/Abstract] OR Kuwait*[Title/Abstract] OR Latvia*[Title/Abstract] OR Liechtenstein*[Title/Abstract] OR Lithuania*[Title/Abstract] OR Luxembourg*[Title/Abstract] OR Monaco*[Title/Abstract] OR Nauru*[Title/Abstract] OR Netherlands*[Title/Abstract] OR New Zealand*[Title/Abstract] OR Norway*[Title/Abstract] OR Oman*[Title/Abstract] OR Palau*[Title/Abstract] OR Poland*[Title/Abstract] OR Portugal*[Title/Abstract] OR Qatar*[Title/Abstract] OR Saint Kitts and Nevis*[Title/Abstract] OR San Marino*[Title/Abstract] OR Seychelles*[Title/Abstract] OR Singapore*[Title/Abstract] OR Slovakia*[Title/Abstract] OR Slovenia*[Title/Abstract] OR Spain*[Title/Abstract] OR Sweden*[Title/Abstract] OR Switzerland*[Title/Abstract] OR Trinidad and Tobago*[Title/Abstract] OR United Arab Emirates*[Title/Abstract] OR United States*[Title/Abstract] OR Aruba*[Title/Abstract] OR Bermuda*[Title/Abstract] OR British Virgin Islands*[Title/Abstract] OR Cayman Islands*[Title/Abstract] OR Cook Islands*[Title/Abstract] OR Curaçao*[Title/Abstract] OR Faroe Islands*[Title/Abstract] OR French Polynesia*[Title/Abstract] OR Gibraltar*[Title/Abstract] OR Greenland*[Title/Abstract] OR Guam*[Title/Abstract] OR Hong Kong*[Title/Abstract] OR Isle of Man*[Title/Abstract] OR New Caledonia*[Title/Abstract] OR Northern Mariana Islands*[Title/Abstract] OR Puerto Rico*[Title/Abstract] OR Saint Martin*[Title/Abstract] OR		

				Taiwan*[Title/Abstract] OR Turks and Caicos Islands*[Title/Abstract] OR U.S. Virgin Islands*[Title/Abstract]))		

16	(((((Child*[Title/Abstract]) OR (((Under five[Title/Abstract]) OR (Under 5[Title/Abstract])) OR (U5[Title/Abstract]))) OR (((Newborn[Title/Abstract]) OR (Infant[Title/Abstract])) OR (1000 days[Title/Abstract]))) AND (((Intervention*[Title/Abstract]) OR (Strateg*[Title/Abstract])) OR (Practice*[Title/Abstract]))) AND (((((((((((((((((((((((Vulnerable[Title/Abstract]) OR (Marginal[Title/Abstract])) OR (Marginalised[Title/Abstract])) OR (Marginalized[Title/Abstract])) OR (Refugee[Title/Abstract])) OR (Homeless[Title/Abstract])) OR (Migrant[Title/Abstract])) OR (financial catastrophe[Title/Abstract])) OR (poverty[Title/Abstract])) OR (conflict-affected[Title/Abstract])) OR (Under attack[Title/Abstract])) OR (Displaced[Title/Abstract])) OR (Temporary Accommodation[Title/Abstract])) OR (Temporary Settlement[Title/Abstract])) OR (Temporary Housing[Title/Abstract])) OR (Transitional Settlement[Title/Abstract])) OR (Transitional shelter[Title/Abstract])) OR (Emergency shelter[Title/Abstract])) OR (Emergency accommodation[Title/Abstract])) OR (Emergency housing[Title/Abstract])) OR (Makeshift shelter[Title/Abstract])) OR (Makeshift accommodation[Title/Abstract])) OR (Slash[Title/Abstract] AND Burn Cultivation[Title/Abstract])) OR (Shifting Cultivation[Title/Abstract])) OR (Feral[Title/Abstract]))) AND (((((((Access[Title/Abstract]) OR (Accessibility[Title/Abstract])) OR (Inaccessible[Title/Abstract])) OR (inaccess[Title/Abstract])) OR (((coverage[Title/Abstract]) OR (outreach[Title/Abstract])) OR (reach[Title/Abstract]))) OR ((((Use[Title/Abstract]) OR (Usage[Title/Abstract])) OR (Utilisation[Title/Abstract])) OR (Utilization[Title/Abstract]))) OR ((Available[Title/Abstract]) OR (Availability[Title/Abstract])))) AND (((((((Feeding[Title/Abstract]) OR (Nutrition[Title/Abstract])) OR (Care practice[Title/Abstract])) OR (Parenting[Title/Abstract])) OR (((dental[Title/Abstract]) OR (wellbeing[Title/Abstract])) OR (mental health[Title/Abstract]))) OR ((((safe sleeping[Title/Abstract]) OR (physical activity[Title/Abstract])) OR (parenting support[Title/Abstract])) OR (sleep hygiene[Title/Abstract]))) OR ((sleep practice[Title/Abstract])))))			("child*"[Title/Abstract] OR ("under five"[Title/Abstract] OR "under 5"[Title/Abstract] OR "U5"[Title/Abstract]) OR ("Newborn"[Title/Abstract] OR "Infant"[Title/Abstract] OR "1000 days"[Title/Abstract])) AND ("intervention*"[Title/Abstract] OR "strategy*"[Title/Abstract] OR "practice*"[Title/Abstract]) AND ("Vulnerable"[Title/Abstract] OR "Marginal"[Title/Abstract] OR "Marginalised"[Title/Abstract] OR "Marginalized"[Title/Abstract] OR "Refugee"[Title/Abstract] OR "Homeless"[Title/Abstract] OR "Migrant"[Title/Abstract] OR "financial catastrophe"[Title/Abstract] OR "poverty"[Title/Abstract] OR "conflict-affected"[Title/Abstract] OR ("Under"[All Fields] AND "attack"[Title/Abstract]) OR "Displaced"[Title/Abstract] OR "temporary accommodation"[Title/Abstract] OR "temporary settlement"[Title/Abstract] OR "temporary housing"[Title/Abstract] OR (("transit"[All Fields] OR "transited"[All Fields] OR "transiting"[All Fields] OR "transition"[All Fields] OR "Transitional"[All Fields] OR "transitionals"[All Fields] OR "transitioned"[All Fields] OR "transitioning"[All Fields] OR "transitions"[All Fields] OR "transits"[All Fields]) AND "Settlement"[Title/Abstract]) OR "transitional shelter"[Title/Abstract] OR "emergency shelter"[Title/Abstract] OR "emergency accommodation"[Title/Abstract] OR "emergency housing"[Title/Abstract] OR ("Makeshift"[All Fields] AND "shelter"[Title/Abstract]) OR ("Makeshift"[All Fields] AND "Accommodation"[Title/Abstract]) OR ("Slash"[Title/Abstract] AND "burn cultivation"[Title/Abstract]) OR "shifting cultivation"[Title/Abstract]		

				OR "Feral"[Title/Abstract]) AND ("Access"[Title/Abstract] OR "Accessibility"[Title/Abstract] OR "Inaccessible"[Title/Abstract] OR "inaccess"[Title/Abstract] OR ("coverage"[Title/Abstract] OR "outreach"[Title/Abstract] OR "reach"[Title/Abstract]) OR ("Use"[Title/Abstract] OR "Usage"[Title/Abstract] OR "Utilisation"[Title/Abstract] OR "Utilization"[Title/Abstract]) OR ("Available"[Title/Abstract] OR "Availability"[Title/Abstract])) AND (Feeding*[Title/Abstract])) OR (nutrition*[Title/Abstract])) OR (care practice*[Title/Abstract])) OR (parenting*[Title/Abstract])) OR (dental*[Title/Abstract])) OR (wellbeing*[Title/Abstract])) OR (mental health*[Title/Abstract])) OR (safe sleeping*[Title/Abstract])) OR (physical activity*[Title/Abstract])) OR (parenting support*[Title/Abstract])) OR (sleep hygiene*[Title/Abstract])) OR (physical activity*[Title/Abstract])		

15	(((((Child*[Title/Abstract]) OR (((Under five[Title/Abstract]) OR (Under 5[Title/Abstract])) OR (U5[Title/Abstract]))) OR (((Newborn[Title/Abstract]) OR (Infant[Title/Abstract])) OR (1000 days[Title/Abstract]))) AND (((Intervention*[Title/Abstract]) OR (Strateg*[Title/Abstract])) OR (Practice*[Title/Abstract]))) AND (((((((((((((((((((((((((Vulnerable[Title/Abstract]) OR (Marginal[Title/Abstract])) OR (Marginalised[Title/Abstract])) OR (Marginalized[Title/Abstract])) OR (Refugee[Title/Abstract])) OR (Homeless[Title/Abstract])) OR (Migrant[Title/Abstract])) OR (financial catastrophe[Title/Abstract])) OR (poverty[Title/Abstract])) OR (conflict-affected[Title/Abstract])) OR (Under attack[Title/Abstract])) OR (Displaced[Title/Abstract])) OR (Temporary Accommodation[Title/Abstract])) OR (Temporary Settlement[Title/Abstract])) OR (Temporary Housing[Title/Abstract])) OR (Transitional Settlement[Title/Abstract])) OR (Transitional shelter[Title/Abstract])) OR (Emergency shelter[Title/Abstract])) OR (Emergency accommodation[Title/Abstract])) OR (Emergency housing[Title/Abstract])) OR (Makeshift shelter[Title/Abstract])) OR (Makeshift accommodation[Title/Abstract])) OR (Slash[Title/Abstract] AND Burn Cultivation[Title/Abstract])) OR (Shifting Cultivation[Title/Abstract])) OR (Feral[Title/Abstract]))) AND (((((((Access[Title/Abstract]) OR (Accessibility[Title/Abstract])) OR (Inaccessible[Title/Abstract])) OR (inaccess[Title/Abstract])) OR (((coverage[Title/Abstract]) OR (outreach[Title/Abstract])) OR (reach[Title/Abstract]))) OR ((((Use[Title/Abstract]) OR (Usage[Title/Abstract])) OR (Utilisation[Title/Abstract])) OR (Utilization[Title/Abstract]))) OR ((Available[Title/Abstract]) OR (Availability[Title/Abstract])))			("child*"[Title/Abstract] OR ("under five"[Title/Abstract] OR "under 5"[Title/Abstract] OR "U5"[Title/Abstract]) OR ("Newborn"[Title/Abstract] OR "Infant"[Title/Abstract] OR "1000 days"[Title/Abstract])) AND ("intervention*"[Title/Abstract] OR "strategy*"[Title/Abstract] OR "practice*"[Title/Abstract]) AND ("Vulnerable"[Title/Abstract] OR "Marginal"[Title/Abstract] OR "Marginalised"[Title/Abstract] OR "Marginalized"[Title/Abstract] OR "Refugee"[Title/Abstract] OR "Homeless"[Title/Abstract] OR "Migrant"[Title/Abstract] OR "financial catastrophe"[Title/Abstract] OR "poverty"[Title/Abstract] OR "conflict-affected"[Title/Abstract] OR ("Under"[All Fields] AND "attack"[Title/Abstract]) OR "Displaced"[Title/Abstract] OR "temporary accommodation"[Title/Abstract] OR "temporary settlement"[Title/Abstract] OR "temporary housing"[Title/Abstract] OR (("transit"[All Fields] OR "transited"[All Fields] OR "transiting"[All Fields] OR "transition"[All Fields] OR "Transitional"[All Fields] OR "transitionals"[All Fields] OR "transitioned"[All Fields] OR "transitioning"[All Fields] OR "transitions"[All Fields] OR "transits"[All Fields]) AND "Settlement"[Title/Abstract]) OR "transitional shelter"[Title/Abstract] OR "emergency shelter"[Title/Abstract] OR "emergency accommodation"[Title/Abstract] OR "emergency housing"[Title/Abstract] OR ("Makeshift"[All Fields] AND "shelter"[Title/Abstract]) OR ("Makeshift"[All Fields] AND "Accommodation"[Title/Abstract]) OR ("Slash"[Title/Abstract] AND "burn cultivation"[Title/Abstract]) OR "shifting cultivation"[Title/Abstract]	14,828	2:31:46

				OR "Feral"[Title/Abstract]) AND ("Access"[Title/Abstract] OR "Accessibility"[Title/Abstract] OR "Inaccessible"[Title/Abstract] OR "inaccess"[Title/Abstract] OR ("coverage"[Title/Abstract] OR "outreach"[Title/Abstract] OR "reach"[Title/Abstract]) OR ("Use"[Title/Abstract] OR "Usage"[Title/Abstract] OR "Utilisation"[Title/Abstract] OR "Utilization"[Title/Abstract]) OR ("Available"[Title/Abstract] OR "Availability"[Title/Abstract]))		
14	((((((Access[Title/Abstract]) OR (Accessibility[Title/Abstract])) OR (Inaccessible[Title/Abstract])) OR (inaccess[Title/Abstract])) OR (((coverage[Title/Abstract]) OR (outreach[Title/Abstract])) OR (reach[Title/Abstract]))) OR ((((Use[Title/Abstract]) OR (Usage[Title/Abstract])) OR (Utilisation[Title/Abstract])) OR (Utilization[Title/Abstract]))) OR ((Available[Title/Abstract]) OR (Availability[Title/Abstract]))			"Access"[Title/Abstract] OR "Accessibility"[Title/Abstract] OR "Inaccessible"[Title/Abstract] OR "inaccess"[Title/Abstract] OR "coverage"[Title/Abstract] OR "outreach"[Title/Abstract] OR "reach"[Title/Abstract] OR "Use"[Title/Abstract] OR "Usage"[Title/Abstract] OR "Utilisation"[Title/Abstract] OR "Utilization"[Title/Abstract] OR "Available"[Title/Abstract] OR "Availability"[Title/Abstract]	4,907,175	2:31:24
13	(Available[Title/Abstract]) OR (Availability[Title/Abstract])			"Available"[Title/Abstract] OR "Availability"[Title/Abstract]	1,358,789	2:30:57

12	(((Use[Title/Abstract]) OR (Usage[Title/Abstract])) OR (Utilisation[Title/Abstract])) OR (Utilization[Title/Abstract])			"Use"[Title/Abstract] OR "Usage"[Title/Abstract] OR "Utilisation"[Title/Abstract] OR "Utilization"[Title/Abstract]	3,385,414	2:30:36
11	((coverage[Title/Abstract]) OR (outreach[Title/Abstract])) OR (reach[Title/Abstract])			"coverage"[Title/Abstract] OR "outreach"[Title/Abstract] OR "reach"[Title/Abstract]	325,002	2:28:26
10	(((Access[Title/Abstract]) OR (Accessibility[Title/Abstract])) OR (Inaccessible[Title/Abstract])) OR (inaccess[Title/Abstract])			"Access"[Title/Abstract] OR "Accessibility"[Title/Abstract] OR "Inaccessible"[Title/Abstract] OR "inaccess"[Title/Abstract]	424,084	2:27:31
9	((((Child*[Title/Abstract]) OR (((Under five[Title/Abstract]) OR (Under 5[Title/Abstract])) OR (U5[Title/Abstract]))) OR (((Newborn[Title/Abstract]) OR (Infant[Title/Abstract])) OR (1000 days[Title/Abstract]))) AND (((Intervention*[Title/Abstract]) OR (Strateg*[Title/Abstract])) OR (Practice*[Title/Abstract]))) AND ((((((((((((((((((((((((Vulnerable[Title/Abstract]) OR (Marginal[Title/Abstract])) OR (Marginalised[Title/Abstract])) OR (Marginalized[Title/Abstract])) OR (Refugee[Title/Abstract])) OR (Homeless[Title/Abstract])) OR (Migrant[Title/Abstract])) OR (financial catastrophe[Title/Abstract])) OR (poverty[Title/Abstract])) OR (conflict-affected[Title/Abstract])) OR (Under attack[Title/Abstract])) OR (Displaced[Title/Abstract])) OR (Temporary Accommodation[Title/Abstract])) OR (Temporary Settlement[Title/Abstract])) OR (Temporary Housing[Title/Abstract])) OR (Transitional Settlement[Title/Abstract])) OR (Transitional shelter[Title/Abstract])) OR (Emergency shelter[Title/Abstract])) OR (Emergency accommodation[Title/Abstract])) OR (Emergency housing[Title/Abstract])) OR (Makeshift shelter[Title/Abstract])) OR (Makeshift accommodation[Title/Abstract])) OR (Slash[Title/Abstract] AND Burn Cultivation[Title/Abstract])) OR (Shifting Cultivation[Title/Abstract])) OR (Feral[Title/Abstract]))			("child*"[Title/Abstract] OR ("under five"[Title/Abstract] OR "under 5"[Title/Abstract] OR "U5"[Title/Abstract]) OR ("Newborn"[Title/Abstract] OR "Infant"[Title/Abstract] OR "1000 days"[Title/Abstract])) AND ("intervention*"[Title/Abstract] OR "strategy*"[Title/Abstract] OR "practice*"[Title/Abstract]) AND ("Vulnerable"[Title/Abstract] OR "Marginal"[Title/Abstract] OR "Marginalised"[Title/Abstract] OR "Marginalized"[Title/Abstract] OR "Refugee"[Title/Abstract] OR "Homeless"[Title/Abstract] OR "Migrant"[Title/Abstract] OR "financial catastrophe"[Title/Abstract] OR "poverty"[Title/Abstract] OR "conflict-affected"[Title/Abstract] OR ("Under"[All Fields] AND "attack"[Title/Abstract]) OR "Displaced"[Title/Abstract] OR "temporary accommodation"[Title/Abstract] OR "temporary settlement"[Title/Abstract] OR "temporary housing"[Title/Abstract] OR (("transit"[All Fields] OR "transited"[All Fields] OR "transiting"[All Fields] OR "transition"[All Fields] OR "Transitional"[All Fields] OR "transitionals"[All Fields] OR "transitioned"[All Fields] OR "transitioning"[All Fields] OR "transitions"[All Fields] OR "transits"[All	35,906	2:24:50

				Fields]) AND "Settlement"[Title/Abstract]) OR "transitional shelter"[Title/Abstract] OR "emergency shelter"[Title/Abstract] OR "emergency accommodation"[Title/Abstract] OR "emergency housing"[Title/Abstract] OR ("Makeshift"[All Fields] AND "shelter"[Title/Abstract]) OR ("Makeshift"[All Fields] AND "Accommodation"[Title/Abstract]) OR ("Slash"[Title/Abstract] AND "burn cultivation"[Title/Abstract]) OR "shifting cultivation"[Title/Abstract] OR "Feral"[Title/Abstract])		

8	((((((((((((((((((((((Vulnerable[Title/Abstract]) OR (Marginal[Title/Abstract])) OR (Marginalised[Title/Abstract])) OR (Marginalized[Title/Abstract])) OR (Refugee[Title/Abstract])) OR (Homeless[Title/Abstract])) OR (Migrant[Title/Abstract])) OR (financial catastrophe[Title/Abstract])) OR (poverty[Title/Abstract])) OR (conflict-affected[Title/Abstract])) OR (Under attack[Title/Abstract])) OR (Displaced[Title/Abstract])) OR (Temporary Accommodation[Title/Abstract])) OR (Temporary Settlement[Title/Abstract])) OR (Temporary Housing[Title/Abstract])) OR (Transitional Settlement[Title/Abstract])) OR (Transitional shelter[Title/Abstract])) OR (Emergency shelter[Title/Abstract])) OR (Emergency accommodation[Title/Abstract])) OR (Emergency housing[Title/Abstract])) OR (Makeshift shelter[Title/Abstract])) OR (Makeshift accommodation[Title/Abstract])) OR (Slash[Title/Abstract] AND Burn Cultivation[Title/Abstract])) OR (Shifting Cultivation[Title/Abstract])) OR (Feral[Title/Abstract])			"Vulnerable"[Title/Abstract] OR "Marginal"[Title/Abstract] OR "Marginalised"[Title/Abstract] OR "Marginalized"[Title/Abstract] OR "Refugee"[Title/Abstract] OR "Homeless"[Title/Abstract] OR "Migrant"[Title/Abstract] OR "financial catastrophe"[Title/Abstract] OR "poverty"[Title/Abstract] OR "conflict-affected"[Title/Abstract] OR ("Under"[All Fields] OR "attack"[Title/Abstract]) OR "Displaced"[Title/Abstract] OR "temporary accommodation"[Title/Abstract] OR "temporary settlement"[Title/Abstract] OR "temporary housing"[Title/Abstract] OR (("transit"[All Fields] OR "transited"[All Fields] OR "transiting"[All Fields] OR "transition"[All Fields] OR "Transitional"[All Fields] OR "transitionals"[All Fields] OR "transitioned"[All Fields] OR "transitioning"[All Fields] OR "transitions"[All Fields] OR "transits"[All Fields]) OR "Settlement"[Title/Abstract]) OR "transitional shelter"[Title/Abstract] OR "emergency shelter"[Title/Abstract] OR "emergency accommodation"[Title/Abstract] OR "emergency housing"[Title/Abstract] OR ("Makeshift"[All Fields] OR "shelter"[Title/Abstract]) OR ("Makeshift"[All Fields] OR "Accommodation"[Title/Abstract]) OR ("Slash"[Title/Abstract] OR "burn cultivation"[Title/Abstract]) OR "shifting cultivation"[Title/Abstract] OR "Feral"[Title/Abstract]	2,716,400	2:24:36

7	(((Child*[Title/Abstract]) OR (((Under five[Title/Abstract]) OR (Under 5[Title/Abstract])) OR (U5[Title/Abstract]))) OR (((Newborn[Title/Abstract]) OR (Infant[Title/Abstract])) OR (1000 days[Title/Abstract]))) AND (((Intervention*[Title/Abstract]) OR (Strateg*[Title/Abstract])) OR (Practice*[Title/Abstract]))			("child*"[Title/Abstract] OR ("under five"[Title/Abstract] OR "under 5"[Title/Abstract] OR "U5"[Title/Abstract]) OR ("Newborn"[Title/Abstract] OR "Infant"[Title/Abstract] OR "1000 days"[Title/Abstract])) AND ("intervention*"[Title/Abstract] OR "strateg*"[Title/Abstract] OR "practice*"[Title/Abstract])	262,165	2:12:15
6	((Intervention*[Title/Abstract]) OR (Strateg*[Title/Abstract])) OR (Practice*[Title/Abstract])			"intervention*"[Title/Abstract] OR "strategy*"[Title/Abstract] OR "practice*"[Title/Abstract]	2,553,193	2:11:54
5	((Child*[Title/Abstract]) OR (((Under five[Title/Abstract]) OR (Under 5[Title/Abstract])) OR (U5[Title/Abstract]))) OR (((Newborn[Title/Abstract]) OR (Infant[Title/Abstract])) OR (1000 days[Title/Abstract]))			"child*"[Title/Abstract] OR "under five"[Title/Abstract] OR "under 5"[Title/Abstract] OR "U5"[Title/Abstract] OR "Newborn"[Title/Abstract] OR "Infant"[Title/Abstract] OR "1000 days"[Title/Abstract]	1,806,063	2:10:53
4	((Newborn[Title/Abstract]) OR (Infant[Title/Abstract])) OR (1000 days[Title/Abstract])			"Newborn"[Title/Abstract] OR "Infant"[Title/Abstract] OR "1000 days"[Title/Abstract]	350,613	2:10:40
3	(Child*[Title/Abstract]) OR (((Under five[Title/Abstract]) OR (Under 5[Title/Abstract])) OR (U5[Title/Abstract]))			"child*"[Title/Abstract] OR "under five"[Title/Abstract] OR "under 5"[Title/Abstract] OR "U5"[Title/Abstract]	1,559,369	2:09:49
2	((Under five[Title/Abstract]) OR (Under 5[Title/Abstract])) OR (U5[Title/Abstract])			"under five"[Title/Abstract] OR "under 5"[Title/Abstract] OR "U5"[Title/Abstract]	16,327	2:09:27
1	Child*[Title/Abstract]			"child*"[Title/Abstract]	1,554,019	2:09:17

Data Extraction Form

(i) Citation Details

(ii) Research Design

(iii) Target Population

a. Sample Size

b. Inclusion

c. Number of participants
d. Number of sites
e. Type of Setting
f. Exclusion criteria
g. Demographics
    i. Age
    ii. Gender

(iv) Target Geography

(v) Intervention
a. Duration
b. Number of groups
c. Channels of Delivery
d. Agency for Delivery
e. Service Points
f. End-Users
g. Scale
h. Other Details

(vi) Evaluation
a. Type of Evaluation
b. Outcome Measures
    i. Primary
        1. ∆Coverage
        2. ∆Access
        3. ∆Utilization
    ii. Secondary
c. Impact Measures
    i. Health
        1. ∆Morbidity
        2. ∆Mortality
        3. ∆DALY
        4. ∆QALI
    ii. Economic
        1. RoI

2. ∆Efficiency
3. ∆Cost

REFERENCES

CUSICK, S. E. & GEORGIEFF, M. K. 2016. The Role of Nutrition in Brain Development: The Golden Opportunity of the "First 1000 Days". *J Pediatr,* 175**,** 16-21.

LEVAC, D., COLQUHOUN, H. & O'BRIEN, K. K. 2010. Scoping studies: advancing the methodology. *Implementation science,* 5**,** 1-9.

MILLER, P. M. 2009. An Examination of the McKinney-Vento Act and Its Influence on the Homeless Education Situation. *Educational Policy,* 25**,** 424-450.

ROSENTHAL DM, L. C., HEYS M, SCHOENTHALER AM, UCCI M, HAYWARD A, ET AL. 2021. Barriers to Optimal Health for Under 5s Experiencing Homelessness and Living In Temporary Accommodation in High-Income Countries: A Scoping Review. *Ann Public Health Res,* 8(1):**,** 1103.

ROSENTHAL, D. M., UCCI, M., HEYS, M., HAYWARD, A. & LAKHANPAUL, M. 2020. Impacts of COVID-19 on vulnerable children in temporary accommodation in the UK. *The Lancet Public Health,* 5**,** e241-e242.
