# Supplementary material for: Interventions to promote the health and well-being of children under 5s experiencing homelessness in high-income countries: a scoping review
Source: BMJ Open. 2024 Jan 12;14(1):e076492. doi: 10.1136/bmjopen-2023-076492 (PMC10806763; doi:10.1136/bmjopen-2023-076492)
Supplement: Author's manuscript [file bmjopen-2023-076492.draft_revisions.pdf]

BMJ Open is committed to open peer review. As part of this commitment we make the peer review history of every article we publish publicly available.

When an article is published we post the peer reviewers' comments and the authors' responses online. We also post the versions of the paper that were used during peer review. These are the versions that the peer review comments apply to.

The versions of the paper that follow are the versions that were submitted during the peer review process. They are not the versions of record or the final published versions. They should not be cited or distributed as the published version of this manuscript.

BMJ Open is an open access journal and the full, final, typeset and author-corrected version of record of the manuscript is available on our site with no access controls, subscription charges or pay-per-view fees (http://bmjopen.bmj.com).

If you have any questions on BMJ Open's open peer review process please email info.bmjopen@bmj.com

Interventions to promote the health and well- being of children under 5s experiencing homelessness in high-income countries: A Scoping Review

Journal:	*BMJ Open*
Manuscript ID	bmjopen-2023-076492
Article Type:	Original research
Date Submitted by the Author:	09-Jun-2023
Complete List of Authors:	Tu, Yanxin; University College London Great Ormond Street Institute of Child Health, Population, Policy and Practice Research and Teaching Department<br>Sarkar, Kaushik; Aceso Global Health Consultants Limited<br>Svirydzenka, Nadia; De Montfort University Faculty of Health and Life Sciences, School of Applied Social Sciences<br>Palfreyman, Zoe; De Montfort University Faculty of Health and Life Sciences, School of Applied Social Sciences<br>Parry, Yvonne; Flinders University, College of Nursing and Health Sciences<br>Ankers, Matthew; Flinders University, College of Nursing and Health Sciences<br>Parikh, Priti; University College London The Bartlett School of Sustainable Construction<br>Raghavan, Raghu; De Montfort University Faculty of Health and Life Sciences, School of Nursing and Midwifery<br>Lakhanpaul, Monica; University College London Great Ormond Street Institute of Child Health, Population, Policy and Practice Research and Teaching Department
Keywords:	Nutrition < TROPICAL MEDICINE, MENTAL HEALTH, Child protection < PAEDIATRICS, Community child health < PAEDIATRICS

SCHOLARONE™
Manuscripts

I, the Submitting Author has the right to grant and does grant on behalf of all authors of the Work (as defined in the below author licence), an exclusive licence and/or a non-exclusive licence for contributions from authors who are: i) UK Crown employees; ii) where BMJ has agreed a CC-BY licence shall apply, and/or iii) in accordance with the terms applicable for US Federal Government officers or employees acting as part of their official duties; on a worldwide, perpetual, irrevocable, royalty-free basis to BMJ Publishing Group Ltd ("BMJ") its licensees and where the relevant Journal is co-owned by BMJ to the co-owners of the Journal, to publish the Work in this journal and any other BMJ products and to exploit all rights, as set out in our licence.

The Submitting Author accepts and understands that any supply made under these terms is made by BMJ to the Submitting Author unless you are acting as an employee on behalf of your employer or a postgraduate student of an affiliated institution which is paying any applicable article publishing charge ("APC") for Open Access articles. Where the Submitting Author wishes to make the Work available on an Open Access basis (and intends to pay the relevant APC), the terms of reuse of such Open Access shall be governed by a Creative Commons licence – details of these licences and which Creative Commons licence will apply to this Work are set out in our licence referred to above.

Other than as permitted in any relevant BMJ Author's Self Archiving Policies, I confirm this Work has not been accepted for publication elsewhere, is not being considered for publication elsewhere and does not duplicate material already published. I confirm all authors consent to publication of this Work and authorise the granting of this licence.

For peer review only

Interventions to promote the health and well- being of children under 5s experiencing homelessness in high-income countries: A Scoping Review

By

Yanxin Tu[1], Kaushik Sarkar[2], Nadia Svirydzenka[3], Zoe Palfreyman[3], Yvonne K. Parry[4], Matthew Ankers[4], Priti Parikh[5], Raghu Raghavan[6], and Monica Lakhanpaul[1*]

*Corresponding author, email: m.lakhanpaul@ucl.ac.uk

Author affiliations

1. Population, Policy and Practice Research and Teaching Department, UCL Great Ormond Street Institute of Child Health, University College London, London, United Kingdom, WC1N 1EH,

2. Aceso Global Health Consultants Limited, Institute for Health Modelling and Climate Solutions, Forecasting Healthy Futures, 4 Bloomsbury Square London, WC1A 2RP

3. School of Applied Social Sciences, Faculty of Health and Life Sciences, De Montfort University, The Gateway, Leicester, United Kingdom, LE1 9BH

4. College of Nursing and Health Sciences, Caring Future Institute, Flinders University, Bedford Park, Sturt South, Adelaide 5001, South Australia, Australia

5. Bartlett School of Sustainable Construction, The Bartlett, UCL Faculty of the Built Environment, 2nd Floor 1-19 Torrington Place London, WC1E 7HB

6. Mary Seacole Research Centre (diversity, culture, and mental health), School of Nursing and Midwifery, Faculty of Health and Life Sciences, De Montfort University, The Gateway, Leicester, United Kingdom, LE1 9BH

For peer review only

ABSTRACT:

Background: Homelessness among families with children, particularly those children aged five years or less residing in temporary accommodation, is a growing global concern, especially in High-Income Countries (HICs). Despite significant impacts on health and development, these "invisible" children often fall through the gaps in policy and services. The study's primary objective is to systematically map the content and delivery methods of culturally sensitive interventions for children under 5 experiencing homelessness. It aims to understand existing intervention strategies, navigate language and cultural barriers, and create accessible materials for this transient and marginalized population.

Method: Arksey and O'Malley's five-stage framework, and the Preferred Reporting Items for Systematic reviews and Meta-Analyses extension for Scoping Reviews (PRISMA-ScR) checklist were drawn on to inform the scoping strategy. Databases include PubMed, MEDLINE, SCOPUS, The Cochrane Library, and Google Scholar were searched up to 24 March 2022. A narrative synthesis was then performed on selected articles.

Results: The database search yielded 951 results. After deduplication, abstract screening, and full review, 13 articles met the inclusion criteria. Two predominant categories of intervention delivery methods were identified in this research: group-based interventions (educational sessions) and individual-based interventions (home visits). Health professionals, trained nurses, and volunteers were often employed as critical points of contact. Research has supported the importance of home-visiting to be instrumental in breaking down language, cultural, and health literacy barriers.

Conclusion This review highlights effective interventions for promoting the health and well-being of children under 5 experiencing homelessness , including educational sessions and home visits. However, it identifies gaps in dental and obesity care targeted at marginalized families in high income countries (HICs), and the need to enhance community resource usage.

Keywords: children under 5s; homelessness; parenting support; interventions; feeding; nutrition; mental health; well-being;

STRENGTHS AND LIMITATIONS:

- This is the first scoping review of peer-reviewed literature focused on intervention approaches specifically for marginalized children under 5s across various themes.
- The review employed a systematic, methodical strategy to extensively map the available evidence and identify knowledge gaps, informing future research.
- Unlike systematic reviews, this review did not conduct formal data synthesis, potentially limiting the comprehensiveness of evidence overview.
- High heterogeneity due to various study designs and low generalizability limit the comparative and broader applicability of the results.

For peer review only

INTRODUCTIONS:

As per the McKinney-Vento definition of homeless, homeless children and youths indicates individuals who lack a fixed, regular, and adequate night-time residence [1]. The UN Office of the High Commissioner for Human Rights, acknowledges that homelessness has "emerged as a worldwide human rights concern," especially in high-income nation-states with the means to address it [2]. Homelessness among families with children is now a growing problem in High-Income Countries (HICs). Countries with Gross National Income (GNI) per capita of $13,206 or more are defined as HICs [3]. Between 2014 and 2018, family homelessness almost doubled in Ireland, rising from 407 to over 1,600 families. Between 2006 and 2013, New Zealand had a 44% rise in family homelessness. In 2018, the United States had around 56,300 families with children, accounting for one-third of the country's homeless population [4]. In 2019, the charity Shelter reported that a child loses their home every 8 min in Great Britain, which is the equivalent of 183 children per day. According to a 2019 report from the charity organization Shelter, one child in Great Britain becomes homeless every 8 minutes. This approximates to 183 children daily [4]. According to the Children's Commissioner, there might be as many as 210,000 homeless children living in temporary accommodation, or couch surfing in England, as well as roughly 585,000 people who are either homeless or in danger of becoming homeless.

In particular, the plight of children aged five years or less residing in temporary accommodation is often overlooked or underrecognized. There is a lack of policy supporting them since they are not seen on the streets as homeless. However, many of the children have pre-existing conditions such as epilepsy, asthma, anxiety, and diabetes, and potentially are the most susceptible to viral infection [4]. Moreover, the first five years of life is a critical duration for the optimal development of the brain. This is especially important for children who experience poverty/housing/transient lifestyle, as this places them at risk of failing to reach the full development of the brain, potentially leading to many health concerns and issues with language development and motor skills and social problems [5].

Our partnership initially concentrated on evidence-based and community-based participatory engagement approaches that enhance the health and wellbeing of children under 5s who are or at risk of being homeless. This was informed by an investigation into how services could adapt to, and learn from, global public health interventions and family experiences, the purpose of which was to firstly tailor current health strategies for effective outreach to populations who are homeless or at risk of homelessness. Then, secondly, in light of both existing and new findings, provide easily accessible resources to health and social care professionals, as well as families of "invisible" children. These children were excluded from research and national policies, as they are often not counted by services, or are not seen as existing as "homeless." However, current literature did not elucidate on the existing intervention strategies, the delivery channels used by these strategies, the language and cultural barriers encountered, the methods for circumventing these barriers, and the creation of acceptable materials for children under 5s. To guide the literature search and review, the authors predefined several themes of importance for the health and wellbeing of homeless children: feeding and nutrition, care practices, dental care, mental health and wellbeing, safe sleeping, physical activity, and parenting support. These themes, identified as common problems in homeless populations and known to have been effectively addressed in other Low- and Middle-Income Countries (LMICs) [6, 7], served as the foundation for this scoping review. These themes are critical for the integrated health of

For peer review only

children under 5s living in temporary accommodation, and there is a complex need to incorporate all these aspects to address or alleviate the current situation of homelessness.

The primary objective of this review is to systematically map the content, and method of delivery of interventions that are culturally sensitive and accessible for populations at the crossroads of poverty/housing/transient lifestyle and focussed on children under 5s. Specifically, our study aims to address the following points:

- Develop inclusive and engaging practice interventions for populations living in temporary accommodation/homelessness, with children under 5s
- Identify the potential critical points of contact. These refer to individuals who play pivotal roles in delivering health services or interventions and engaging with the target population. They serve as key intermediaries, bridging the gap between the health system or intervention and the individuals it aims to benefit.
- Understand how to communicate with mobile populations who have poor health knowledge, literacy, and/or language barriers
- Identify methods of creating appropriate, acceptable, and accessible communication materials

METHODS:

This focused and methodical scoping review, which was enriched by insights and real-world experiences from global experts, followed the guidelines set forth by the Arksey and O'Malley framework [8] as well as the PRISMA-ScR checklist for scoping reviews [9].

1. Eligibility Criteria

The inclusion/exclusion criteria for this scoping review are outline in the table below:

Table 1. Summary of Eligibility Criteria for Literature Search.

Inclusion	Exclusion
English language	Language other than English
Studies from High Income Countries(HIC) as established by the World bank [3]	Countries not included in the World bank's list of HIC's [3]
Interventions focused on the $\leq$ 5 years	Interventions on the > 5 years
Children from marginalized or socially excluded families/population groups	Children/families from non-marginalized or socially excluded backgrounds.
Studies which describe, measure, or evaluate a pilot or implementation of a strategy,	Those not listed.

For peer review only

tactics, process, and/or method targeted at improving child health programmes	
Interventions that improved outcomes, including service coverage and optimization, access, and utilization, and that focus on the nine identified areas of - 1. Feeding, 2. Nutrition, 3. Care practice, 4. Parenting, 5. Dental, 6. Wellbeing, 6. Mental health, 7. Safe sleeping, 8. Physical activity, and 9. Parenting support	Intervention that made no difference or did not help in the outlined areas
Date range 2000 to 2022	Studies from outside the specified date range

By adhering to these eligibility criteria, the scoping review will ensure that the findings are based on the highest quality evidence and are relevant to the topic of interest. In case the number of these studies is less than 30, we will broaden our investigation to include studies on interventions aimed at children over the age of 5, but only if they offer valuable insights or strategies that can be adapted for younger age groups.

2. Information Sources and Search Strategy

The databases searched from inception of this project up to 24 March 2022 included: PubMed, MEDLINE, SCOPUS, The Cochrane Library (Cochrane Database of Systematic Reviews, Cochrane Central Register of Controlled Trials [CENTRAL]), and Google Scholar. One researcher (YT) developed the search strategy after preliminary deliberations and consensus within the review team. Conference abstracts and Third Sector Reports were also searched for grey literature. Experts from Donor/ Philanthropic organizations and other key experts were contacted for any additional published or unpublished work.

The search strategy was developed to ensure that any relevant studies were identified helping to make the review exhaustive. In the search for literature on the homeless population, two concepts were utilized: 'marginalization' and 'social exclusion'. The search strategy was comprised of key search terms drawn together into search strings and then customized for each individual electronic database. The authors also conducted relational and citation searches to screen cited articles of the 1st iteration of selections, followed by citations and related articles for each inclusion in PubMed, Google Scholar, and PubMed respectively.

3. Study Selection

Search results were uploaded to the Covidence software management system [10] where several reviewers screened content. All articles were extracted and compiled in a single spreadsheet. The spreadsheet was equipped with duplicate study filters, and inclusion filters (inclusion criteria not managed through Search Term) and selection columns. Inclusion and

For peer review only

selection filters were filled in independently by one reviewer (YT). All studies selected by two reviewers in Covidence were included, while all studies rejected by two reviewers in Covidence, were rejected. All studies marked for inclusion by one reviewer were marked as disputed selection. In the case of disputes, a third expert reviewer (ML) made the final inclusion decision. Two round of screening was undertaken: Title-Abstract (TiAb) and Full Text (FT). The reasons for exclusion was recorded and mapped in a flow chart, as per PRISMA guidelines.

4. Data charting process

A data extraction form (DEF) was developed as per CEB Critically Appraised Topics (CAT) guidelines by KS and piloted by YT on the first 6 article included. The piloting essentially informed whether the DEF could extract necessary and sufficient information as per the objectives set. The form was then amended based on the pilot and the finalized form used to extract data from full-text articles. The authors abstracted data on article characteristics (target geography, study design), demographic characteristics (age and gender), and intervention characteristics (number of participants, number of sites, type of setting, duration, channels and agency of delivery, service point, end-users).

5. Methodological Quality Appraisal

The methodological quality of included articles was assessed using different tools, relative to the article type. For example, the assessment of Risk of Bias (RoB) tool was only used for interventional studies when results were quantitative analysis or pooling. GRADE [11] was used for assessment of bias in selective publications and selective non-reporting for systematic reviews. For randomized trials, Cochrane RoB 2.0 [12] was used to assess multiple sources of bias and RoBANS [13] was used for assess bias due to selective non-reporting and bias in the selection of the reported result. All studies were critically appraised as per the CAT grading.

RESULTS:

1. The Literature Search

The database search yielded a total of 951 results; no additional articles were found in the grey literature search (Figure 1). 523 records were evaluated after deduplication. After screening title and abstract, 433 relevant articles were excluded. This resulted in 90 articles for review, of which 77 were excluded, as they did not meet the inclusion and exclusion criteria (Table 1).

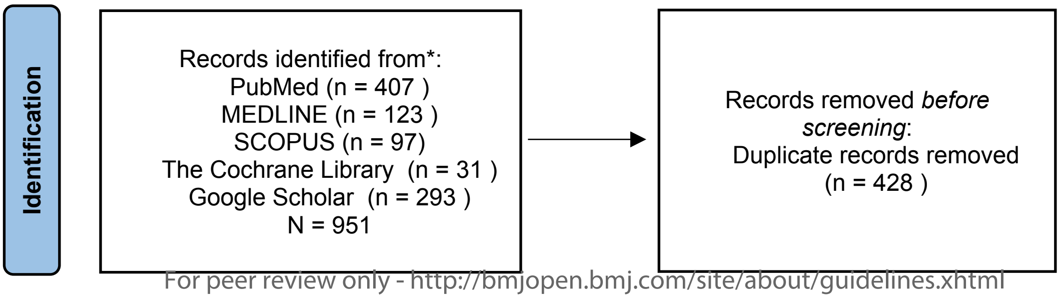

For peer review only

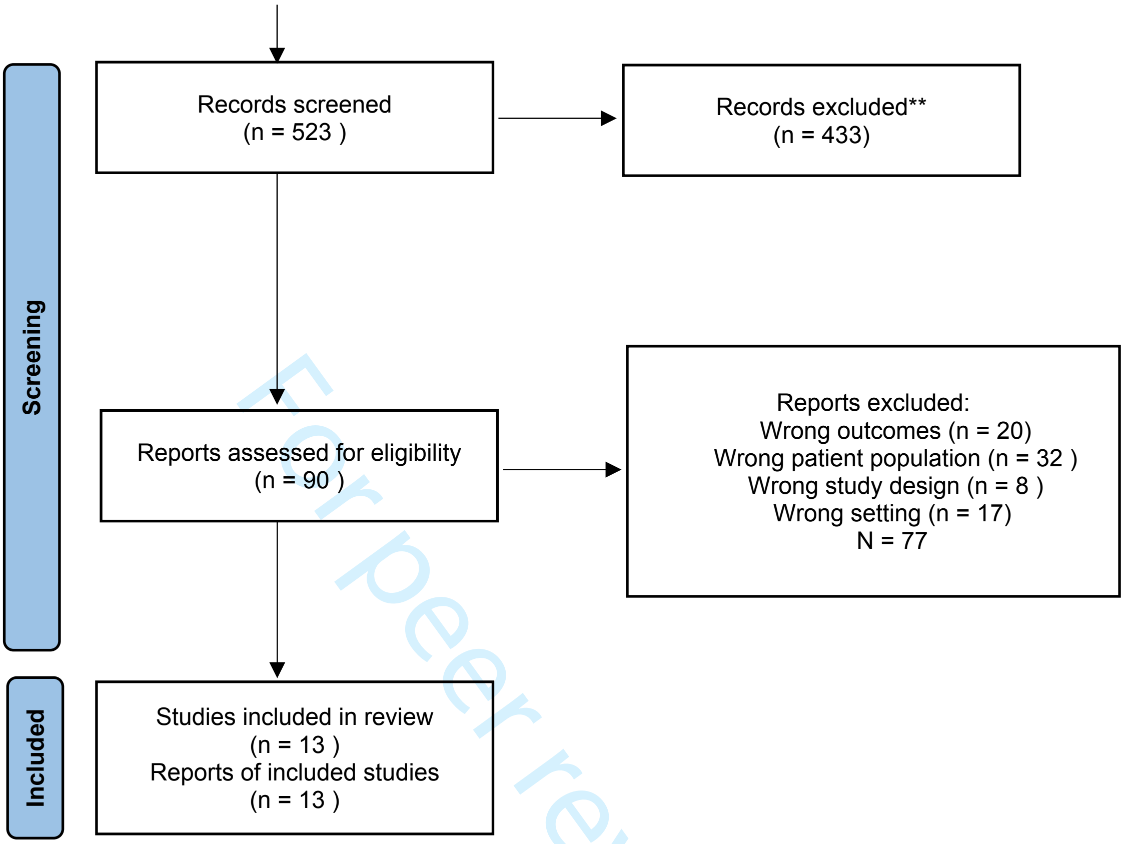

Figure 1. PRISMA 2020 Flow Diagram.

For peer review only

2. Data Summary and Synthesis

Data from included studies were collated, and Table 2 presents characteristics and conclusions reported.

Table 2. Summarization in data extraction of included studies.

Authors	Age	Location	Sample size	Contact Points	Outcomes	Intervention focus
Burgi et al., 2012	5.2 years (SD=0.6)	Switzerland	N =652	Health promoters (volunteers) and preschool teacher	Reduce adiposity and improve fitness, nutrition, media use, safe sleep	Sessions of Physical activity (PA) for children, workshops for teachers, interactive information and discussion evenings for parents
Foka et al., 2021	7-12 years	Greece	N =72	Trained facilitators (volunteers)	Promoting well-being (optimistic thinking and hope, mindfulness) and resilience in refugee children	An intervention aimed at fostering resilience: a group-focused, interactive, and educational non-clinical program led by a trained female facilitator along with a local interpreter who is a refugee
Goodman et al., 2022	6 months	USA	N =1243	Trained nurses and staff	Community service utilization, positive parenting support, infant health and wellbeing, parent health and wellbeing	Family Connects (FC): A home visit of two hours duration is implemented with the aim to enhance the health and overall well-being of families having infants and young children
Grace et al., 2019	15 months	Australia	N =363	Trained volunteer	Social isolation, community	A weekly 2-hour home visit is conducted by a trained

For peer review only

					connectedness, parenting competence	volunteer. Each volunteer has undergone a minimum of 30 hours of preparatory training prior to being paired with a family
Gross et al., 2009	2-4 years	USA	N =292	Trained volunteer	Enhances parental responses that amplify positive recognition for preferred child conduct and diminishes severe and inconsistent reactions to challenging child behavior	The program uses recorded video scenarios and facilitates group discussions that are related to the principles presented in each scenario. It is structured as 11 weekly sessions, with a supplemental session offered two months after the final regular session
Guerrero et al., 2021	0-12 years old	USA	N =1749	Trained staff (volunteer)	Enhance parents' understanding and perception of chronic stress and depression, with a focus on the frequency of depressive episodes and the impact on children's behaviors	The method involves educational sessions, supplemented with annotated slides for low literacy audiences, a concise four-page brochure, and tip sheets on stress and depression topics
Holtrop and Holcomb, 2018	3.18 years old (SD=1.66)	USA	N =12	Marriage and family therapist (health professionals)	Bolster positive parenting practices, address child socioemotional,	The Oregon Model of PMTO encourages five parenting strategies (skill encouragement, boundary setting, monitoring, problem-solving, active participation) through

For peer review only

					behavioral, and academic problems.	educational sessions, practice tasks, and satisfaction surveys
Melley, 2010	2 weeks - 3 years old	USA	*N* =87	Trained nurses and parents	Improve Children's resilience during and after the stress of homelessness and shelter living	6 intervention methods combined: weekly Parent–Child Therapy Sessions; Family Ties Breakfast; Separation Practice; Attachment-Based Circle Time; Parent-Child Activity; Parent Group
Ristkari et al., 2019	4 years old	Finland	*N* =463	Professionals in health care and social services	(1) fundamental affirmative parenting techniques, (2) applicable parental abilities, and (3) reinforcement of learned skills to sustain beneficial parenting and alleviate disruptive behavior	Web-based (online sessions, e-mail) parent training intervention with follow-up telephone coaching
Rowe et al., 2012	0 to 4 years old	Australia	*N* =116	Professionals specializing in early childhood and nurses with extensive expertise in maternal and child health.	Positive parenting skills (sleeping and settling, feeding and meal time, establishing routines)	Tweddle Child & Family Health Service (TCFHS): one on one sessions, group sessions, and secondary consults<br>The Day Stay Program (DSP) service: group level educational based sessions

For peer review only

Dugravier et al., 2013	prenatal to 24 months old	France	*N* =440	Phycologists (health professionals)	Enable mothers to build their parenting abilities (focusing on attachment), efficiently utilize health and social services, and fully leverage their personal connections and local community resources	In the CAPEDP model, a specialized group of psychologists conduct home visits to foster mental wellness and quality attachment, offer socio-emotional assistance within a robust working relationship, and manage depression if it arises. The designated time frame for this study included a total of 14 planned home visits
Spijkers et al., 2010	9-11 years	Netherland	*N* =160	Trained nurses	Promote children's social, emotional, and behavioral development	Multilevel parenting program: individual consultations, group level educational sessions, home-visiting, parent-children interaction method
Yousey et al., 2007	18 months-6 years old	USA	*N* =56	Trained nurses	To improve the nutritional status of homeless children by improving nutrition knowledge of parents and food providers in homeless shelter	Education sessions was developed by health professional (dietitian), 4 sessions for mother, 3 lessons for cafeteria staff

For peer review only

3. Quality Review

Based on the screening results of Cochrane RoB 2.0, the study by Grace et al. exhibited a risk of performance bias. Caregivers and personnel administering the interventions were aware of participants' allocated intervention throughout the trial, as this information was disclosed by participants in their questionnaire responses about their experiences with services [14]. Furthermore, there was a risk of selection bias in the study by Gross et al. due to issues with the randomization process. Parents were not randomly assigned to different intervention levels, but instead chose how many group sessions they attended. Consequently, observed improvements in child behaviour might be attributed to factors associated with parental attendance, rather than the intervention itself [15]. Study of Goodman et al. is limited by the use of self-report data, which could contribute to recall and social-desirability bias [16]. Concerns were also raised regarding bias due to missing data for study by Dugravie et al. Only half of the participants completed their perinatal home-visiting program [17].

In light of the screening results of RoBANS, study of Guerrero et al. is also constrained by the use of self-report data [18]. The challenge of retaining parents was also a notable drawback of the research by Holtrop et al., since only 50 of the participants attended more than fifty percent of the sessions [19]. In general, we did not exclude any studies based on risk of bias assessment.

4. Characteristics of Intervention Programmes

4.1 Intervention Themes

In total, 13 studies were included for review (Table 2). Of the nine targeted intervention themes in the inclusion criteria, none pertaining to dental outcomes for marginalized children in HICs were identified in the search. Several overlapping themes, such as feeding, nutrition, safe sleeping, and mental health, were commonly addressed through enhancing positive parenting skills and support. Overall, four intervention theme categories were identified from the evidence synthesis.

- Positive Parenting Skills (sleeping and settling, safe sleep, feeding and meal time, establishing routines, attachment and boding) [15, 16, 19-22]
- Mental Health and Wellbeing (social isolation, chronic stress and depression, toddler behavior) [14, 17, 18, 23, 24]
- Nutrition (Improve nutrition knowledge of parents) [25]
- Physical Activity (life style, adiposity and fitness) [26]

4.2 Characteristics of Participants

Upon an initial exploration of the literature, the number of studies within this age range was limited. Given the importance of our research question and the potential benefits of gaining a

For peer review only - http://bmjopen.bmj.com/site/about/guidelines.xhtml

broader understanding of the available interventions, this study encompassed studies involving children above five. The majority of the studies encompassing children aged under 5-year-olds [14-22, 25, 26], 2 studies included were targeted on early childhood (ages 6 -11) [23, 24]. The justification for this approach lies in the potential transferability of interventions for slightly older children to younger age groups.

Gender distribution is generally even and homogeneous across different study. However, in a non-randomized intervention study by Holtrop et al. [19], the sample consisted of 75% females and 25% males. Gender distribution was not specified in the studies by Yousey et al. and Guerrero et al. [18, 25]. The backgrounds of the participants varied widely across the included studies. Participants encompassed children of migrant and/or parents with low education levels, children from homeless or poverty-stricken families, new-born infants from transitional housing communities, and families living in low socioeconomic areas.

4.3 The Method of Delivery

Detailed description of the intervention programmes are shown in Table 2. The intervention duration varies from 1 week to 2 years, with four included studies having intervention durations of over 12 months [14, 15, 17, 26], four studies ranging from 6 to 12 months [16, 18, 19, 24], and five studies lasting less than 6 months [20-23, 25]. The interventions identified in this study have been broadly categorized as follows:

- Education-based group interventions, performed as group-based intervention, such as those based on the Webster-Stratton Model [27] and the Oregon Model [28], are effective in improving parenting practices and child behaviour, especially in socio-economically disadvantaged communities [15, 19, 20, 22, 25, 26]. These include methods such as web coaching, videotaped vignettes, group discussions, and family meetings. Broadening the intervention scope to include other involved adults and offering education on nutrition, physical activity, media use, and sleep further boosts their efficacy. Programs like the Strengths for the Journey in refugee camps have utilized positive psychology concepts[23], while the Parent-Child Interaction Therapy model [29] successfully strengthened parent-child relationships in shelters. Overall, these education-based group interventions offer a cost-effective, holistic approach to child welfare.

- Home-visiting interventions involved trained professionals offering support and education to families with young children in their homes, with interventions being adapted to each family's unique needs, often complemented by group-based interventions. The goal was to foster community connectivity and independent utilization of services [14, 16, 17]. Some studies, such as Guerrero et al.'s work, supplement these home visits with group-based education, training staff and parents in stress and depression management via a flexible, Train-the-Trainer approach [18]. Additionally, the Positive Parenting Program (Triple P) employs a multilevel system of family intervention combining home-visiting and education session approaches, escalating in intensity based on the severity of the child's behavioural and emotional issues [24].

For peer review only

4.4 Critical Points of Contact

Marginalized children may face a variety of barriers that can prevent them from accessing healthcare, education, social services, and other resources that are necessary for their well-being. Designing contextual, culturally sensitive, and diverse interventions remains a challenge. However, identifying effective points of contact for interventions can help overcome these obstacles. Studies have utilized maternal and child health nurses [16, 20, 21, 24, 25], and health professionals with relevant expertise [17, 19, 20, 22], as critical points of contact. Trained volunteers, often acting as health promoters or facilitators, have also served this role [14, 15, 18, 23, 26]. Nurses and trained volunteers are cost-effective, easy to train and access. Health professionals typically possess greater knowledge, skills, and experience, adhering to strict professional standards. It was hypothesized that professionals who were more highly trained in interested field would be more competent in implementing interventions precisely and effectively [10]. The choice of critical points of contact hinges on the study's complexity and scope, warranting a cost-effectiveness analysis.

5. Communication with Poor Health Literacy Population

This review notably aimed to explore strategies for creating suitable, acceptable, and accessible communication materials, especially for mobile populations with limited health knowledge, literacy, or language skills, particularly those with children under five. In relation to the development of such materials, the Chicago Parent Program (CPP), as described in the study by Gross et al., devised an intervention content and strategy. Communication materials were created in partnership with an advisory council of parents, including seven African-American and five Latino parents from different neighbourhoods in Chicago. This council provided input to the program creators about their parenting challenges, the scenarios they'd prefer to see in video format, and the best ways to present parenting techniques that align with their values, lifestyle, and cultural norms. This approach made the content patient-oriented, readily acceptable, and culturally sensitive [15]. Yousey et al. and Guerrero et al. discussed making teaching materials, handouts, and class activities in a low-literacy format, visually appealing (with pictures and varied colours), and game-oriented (puzzles, riddles) as useful strategy that make acceptable, and accessible communication material [18, 25]. As materials developed for marginalized parents with poor health literacy or low education levels, it is important to provide information in different languages. Burgi et al. also equipped native speakers of the main foreign languages to answer any questions from parents throughout the intervention to break language barriers [26]. In terms of the communication method with marginalized population, the reviewed articles emphasized the importance of home-visiting, as a less formal, relationship-based approach is potentially crucial in overcoming barriers to service engagement, such as language and cultural obstacles, and limited health literacy [14, 16, 17, 24].

For peer review only

DISCUSSIONS:

1. Group-based interventions

Educational sessions were the most commonly employed strategy in group-based interventions included in this scoping review. Providing educational sessions in group level can be a valuable and effective approach to supporting families with children under 5, who experience homelessness in high-income countries. Families experiencing homelessness may feel isolated and disconnected, and participating in a group-based intervention can provide an opportunity for them to connect with other families in similar situations [14]. Therefore, group-based interventions can provide families with a sense of social support and community. Educational sessions can also be tailored to meet the specific needs of families experiencing homelessness with children under 5, since the content of sessions are dependent on the health needs. In short, group-based educational session interventions can empower families experiencing homelessness by providing them with the tools, knowledge, and support they need to improve their situation.

Web-based sessions are an innovated form of carrier of education and thus modifying behaviours and improve health outcomes in children under 5 experiencing homlessness. Parent training programs that utilize technology can have numerous advantages compared to conventional interventions, including improved consistency, enhanced availability, increased convenience, and a reduction in both time and financial expenditure [30, 31]. The concept of technology-based parent training isn't new; dating back to 1988, Webster-Stratton and his team [27] used video recordings as the main medium to conduct a parent training initiative. The innovation of web-based sessions offers an efficient platform for imparting necessary knowledge, modifying behaviours, and improving health outcomes in the children under 5 living in temporary accommodation. Given the high usage of cell phones among the homeless population in High-Income Countries (HICs), the potential for web-based interventions becomes even more evident. This suggests a promising intersection between technology and accessible parent training programs within the context of homelessness. Evidence indicated that most people experiencing homelessness have cell phones in HICs. In a case report conducted in Los Angeles, USA, 85% of homeless people used a cell phone and used text messaging daily, and 51% accessed the Internet on their cell phone [32]. A number of other studies have postulated a convergence on the similar findings [33-35]. The current cell phone using status support the possibility of Web-based intervention use in the homelessness context. Web-based interventions can potentially eliminate the obstacles related to in-person sessions, allowing individuals to pursue assistance for mental health issues without the concern of stigma [36-38]. The web-based intervention was demonstrated to be low cost, low threshold and of great implications for evidence-based interventions in the future. Accordingly, recent studies in the sphere of parent training have pivoted towards online training programs. These interactive online training platforms for parents can successfully surmount numerous obstacles typically encountered during the execution phase of traditional programs, suggesting a promising intersection between technology and accessible parent training programs within the context of homelessness. [22].

For peer review only

2. Individualized interventions

Parents, and particularly mothers, are susceptible to social isolation, especially during the initial transition to parenthood when they may experience intense feelings of fatigue or a lack of readiness. In research concerning parents facing additional challenges, such as parenting a child with a disability, recent immigration, experiencing cognitive or mental health difficulties, the prevalence of social isolation was found to be significant [39-41]. To address this issue, one commonly utilized strategy is home-visiting. Typically coordinated by a local organization, this intervention involves assigning a volunteer or professional to provide social support to individuals identified as needing additional help. Through regular visits, these health providers offer a variety of support services and work towards enhancing the individual's engagement with formal services, fostering greater community connection, and promoting independent utilization of services.

Individualized interventions (home-visiting) can be more flexible than group-based interventions, as they can be adapted to meet the changing needs of families over time, provide personalized support tailoring to different family's needs, provide families with access to a range of resources and services, and provide a greater sense of confidentiality and privacy. These individualized approaches are instrumental in dismantling barriers to service engagement, including those tied to language and culture [42]. Recent studies advocate for the possible benefits of home visits by volunteers, such as facilitating the dissemination of health-related information [43], enhancing social networks for those in isolation, fostering emotional wellbeing and parental proficiency, and endorsing positive health outcomes [44]. Furthermore, previous research has emphasized the value of home-visiting, a less formal, relationship-based approach that complements other more structured services in the service landscape [42]. This idea suggests that combining group-level educational sessions with home visits could reinforce training content by allowing for individual feedback collection and catering to personal needs. Given these potential benefits of individualized interventions, the strategy of home-visiting has gained attention in both research and practice. By bridging the benefits of both individualized interventions and community support, home-visiting appears to be a promising strategy that could be more widely applied in future intervention designs for homeless population.

3. Research Gap

While there's a growing focus on understanding barriers to dental services for the homeless population in High-Income Countries (HICs), it's evident that service provision, particularly for homeless children, is woefully inadequate [45-47]. Studies have shown a lack of dental health care services for the homeless across governmental, private, and third-sector levels [45]. Moreover, current peer-reviewed literature is sparse on strategies improving access to, and uptake of, dental care for this marginalized group [48]. A scoping review of grey literature identified only two services specifically catering to the homeless population in Australia [48]. The effectiveness of these services remains unknown, raising questions about their generalizability across various geographic settings or age groups. The issue is pressing considering the vulnerability of marginalized children to adverse dental problems. An estimated three million children in Europe are believed to receive inadequate dental treatment [49], and children from low-income families are twice as likely to have cavities compared to

For peer review only

those from higher-income households [50]. Consequently, there is an urgent public health need to implement targeted dental services for young children within the homeless population.

In the context of our scoping review, another noticeable gap is that only one study focused specifically on the theme of physical activity [26]. This paucity of research is a concern given the global public health issue of childhood overweight and obesity [51]. In addition, children of migrant parents and those with low socioeconomic status are considered high risk groups for the development of obesity and of low fitness, which is known as the hunger-obesity paradox [52]. In addressing this issue, it was scoped that Burgi et al. developed a lifestyle intervention in Switzerland aimed at enhancing physical activity level in preschool children, primarily from migrant or lower education level (EL) families [26]. This intervention showed beneficial effects, yet it remains an outlier; overall, interventions in these populations have shown less effectiveness [53-56]. Given the magnitude of the obesity and low fitness issue among homeless populations, the paucity of targeted interventions underlines a pressing need for further research. Future studies should aim to develop effective, evidence-based approaches that integrate health promotion programs within the broader context of social and cultural values. This could help in designing interventions tailored to this vulnerable population, thereby addressing this significant gap in the research.

4. Strengths and Limitations

This study represents the first scoping review of peer-reviewed literature dedicated specifically to intervention approaches for marginalized children living in temporary accommodation under five years old across various themes. We deployed a rigorous and systematic strategy to extensively map available evidence, with the aim of identifying knowledge gaps to inform future research. The quality of all included studies was high, underscoring the credibility of our findings. Despite these strengths, several limitations should be acknowledged. Unlike systematic reviews, scoping reviews typically do not conduct formal data synthesis. Consequently, the results may not provide as comprehensive or robust an overview of the evidence as systematic reviews do. Furthermore, this review exhibits a high level of heterogeneity due to the incorporation of studies with diverse designs and methodologies, which can make comparing and synthesizing results challenging. Lastly, the review may suffer from limited generalizability due to the specific demographics and settings of the included studies.

CONCLUSIONS:

This review sheds light on health interventions that effectively reach children under five years old who are homeless or at risk of homelessness. It contributes not only to the literature but also provides actionable resources for health and social care professionals and the families of these often 'invisible' children. While a robust body of research focuses on parenting support, mental health, well-being, nutrition and feeding, and care practices, we found a significant gap in addressing dental health and over-weight within marginalized families in HICs. The review of thirteen interventions revealed that two primary methods, group-based educational sessions

For peer review only

and individual-based home visits, hold promise. Notably, an integrated approach combining these two methods appears to be feasible. It was also identified that health professionals, nurses, and trained volunteers play crucial roles in delivering these interventions. Despite these insights, several areas necessitate further investigation. For example, a comparison of cost-effectiveness between group-based and individualized interventions remains elusive. Moreover, the review identified a common issue of low community connectedness and service utilization, highlighting the need to explore ways to enhance community resource usage in HICs further.

Acknowledgements

We express our profound gratitude to Dr. Kaushik Sarkar, whose invaluable guidance and expertise were instrumental in shaping this study. His leadership in developing the study protocol and formulating the search strategy played a critical role in the success of our research. We wish to thank all team members of the ESRC funded Champions project through which the need for this review was recognised.

Contributors

Each author made substantial contributions to the design and execution of the study, and to the writing and revising of this article. YT played a primary role in conceptualizing the study, data collection and analysis, and drafting and revising the manuscript. KS, ML and NS guided the study protocol and developed the search strategy. KS was actively involved in data collection and analysis. ML and NS were key contributor to the study conception and design, guided data analysis, and revised the manuscript. PP, ZP, YP, MA, and RR were responsible for all major areas of concept development and study planning, were consulted on the data analysis and its interpretation and provided manuscript edits. All authors reviewed and finalised the manuscript.

Funding

This work was supported by the ESRC as part of UK Research & Innovation's rapid response to COVID-19 (ES/V016253/1). Monica Lakhanpaul was also supported by the National Institute for Health Research (NIHR) Biomedical Research Centre based at UCL Great Ormond Street Institute of Child Health/Great Ormond Street Hospital NHS Foundation Trust and the NIHR Applied Research Collaboration (ARC) North Thames.

Competing interests

The authors report that there are no competing interests to declare.

Patient and public involvement

Not applicable.

Patient consent for publication

Not applicable.

For peer review only

Ethics approval

Research ethics approval was not required as this study did not involve participants.

Provenance and peer review

Not commissioned; externally peer reviewed.

Data availability statement

Data are available upon reasonable request to the corresponding author (https://orcid.org/0000-0002-9855-2043).

For peer review only

REFERENCES:

1. Miller, P.M., *An examination of the McKinney-Vento Act and its influence on the homeless education situation.* Educational Policy, 2011. **25**(3): p. 424-450.
2. housing, S.R.o.t.r.t.a. *Report on homelessness as a global human rights crisis*. 2015 [cited 2022 04/26]; Available from: https://www.ohchr.org/en/calls-for-input/report-homelessness-global-human-rights-crisis.
3. Bank, W., *World Bank country and lending groups—World Bank data help desk.* World Bank Country and Lending Groups–World Bank Data Help Desk, 2021.
4. Rosenthal, D.M., et al., *Impacts of COVID-19 on vulnerable children in temporary accommodation in the UK.* The Lancet Public Health, 2020. **5**(5): p. e241-e242.
5. Cusick, S.E. and M.K. Georgieff, *The role of nutrition in brain development: the golden opportunity of the "first 1000 days".* The Journal of pediatrics, 2016. **175**: p. 16-21.
6. Zlotnick, C., T. Tam, and S. Zerger, *Common needs but divergent interventions for U.S. homeless and foster care children: results from a systematic review.* Health Soc Care Community, 2012. **20**(5): p. 449-76.
7. Weber, J.J., *A systematic review of nurse-led interventions with populations experiencing homelessness.* Public Health Nurs, 2019. **36**(1): p. 96-106.
8. Levac, D., H. Colquhoun, and K.K. O'Brien, *Scoping studies: advancing the methodology.* Implementation science, 2010. **5**: p. 1-9.
9. Tricco, A.C., et al., *PRISMA extension for scoping reviews (PRISMA-ScR): checklist and explanation.* Annals of internal medicine, 2018. **169**(7): p. 467-473.
10. Innovation, V.H. *Covidence systematic review software, Melbourne, Australia. Available online: www.covidence.org.*
11. Morgan, R.L., et al., *A risk of bias instrument for non-randomized studies of exposures: a users' guide to its application in the context of GRADE.* Environment international, 2019. **122**: p. 168-184.

12. Sterne, J.A., et al., *RoB 2: a revised tool for assessing risk of bias in randomised trials.* bmj, 2019. **366**.
13. Park, J., et al. *Risk of bias assessment tool for non-randomized studies (RoBANS): development and validation of a new instrument*. in *19th Cochrane Colloquium*. 2011.
14. Grace, R., et al., *Effectiveness of the Volunteer Family Connect Program in Reducing Isolation of Vulnerable Families and Supporting Their Parenting: Randomized Controlled Trial With Intention-To-Treat Analysis of Primary Outcome Variables.* JMIR Pediatr Parent, 2019. **2**(2): p. e13023.
15. Gross, D., et al., *Efficacy of the Chicago parent program with low-income African American and Latino parents of young children.* Prev Sci, 2009. **10**(1): p. 54-65.
16. Goodman, W.B., et al., *Evaluation of a Family Connects Dissemination to Four High-Poverty Rural Counties.* Matern Child Health J, 2022.
17. Dugravier, R., et al., *Impact of a manualized multifocal perinatal home-visiting program using psychologists on postnatal depression: the CAPEDP randomized controlled trial.* PLoS One, 2013. **8**(8): p. e72216.
18. Guerrero, A.D., et al., *Evaluation of a Health Education Intervention to Improve Parental Knowledge and Attitudes About Chronic Stress and Depression Among Head Start Families.* Health Promot Pract, 2021: p. 15248399211061132.
19. Holtrop, K. and J.E. Holcomb, *Adapting and Pilot Testing a Parenting Intervention for Homeless Families in Transitional Housing.* Fam Process, 2018. **57**(4): p. 884-900.
20. Rowe, H., et al., *Admission to day stay early parenting program is associated with improvements in mental health and infant behaviour: A prospective cohort study.* Int J Ment Health Syst, 2012. **6**(1): p. 11.
21. Melley, A.H.C., Kim; Norris-Shortle, Carole; Kiser, Laurel J.; Levey, Eric B.; Coble, Catherine A.; Leviton, Audrey, *Supporting Positive Parenting for Young Children Experiencing Homelessness: The PACT Therapeutic Nursery.* Zero to Three, 2010. **30**(4): p. 39-45.
22. Ristkari, T., et al., *Web-Based Parent Training Intervention With Telephone Coaching for Disruptive Behavior in 4-Year-Old Children in Real-World Practice: Implementation Study.* J Med Internet Res, 2019. **21**(4): p. e11446.
23. Foka, S., et al., *Promoting well-being in refugee children: An exploratory controlled trial of a positive psychology intervention delivered in Greek refugee camps.* Dev Psychopathol, 2021. **33**(1): p. 87-95.
24. Spijkers, W., D.E. Jansen, and S.A. Reijneveld, *Effectiveness of Primary Care Triple P on child psychosocial problems in preventive child healthcare: a randomized controlled trial.* BMC medicine, 2013. **11**(1): p. 1-8.
25. Yousey, Y., et al., *Education in a homeless shelter to improve the nutrition of young children.* Public Health Nurs, 2007. **24**(3): p. 249-55.
26. Burgi, F., et al., *Effect of a lifestyle intervention on adiposity and fitness in socially disadvantaged subgroups of preschoolers: a cluster-randomized trial (Ballabeina).* Prev Med, 2012. **54**(5): p. 335-40.

27. Webster-Stratton, C., *Preventing conduct problems in Head Start children: strengthening parenting competencies.* Journal of consulting and clinical psychology, 1998. **66**(5): p. 715.

28. Forgatch, M.S. and G.R. Patterson, *Parent Management Training—Oregon Model: An intervention for antisocial behavior in children and adolescents.* 2010.

29. Fernandez, M.A. and S.M. Eyberg, *Predicting treatment and follow-up attrition in parent–child interaction therapy.* Journal of abnormal child psychology, 2009. **37**(3): p. 431-441.

30. Breitenstein, S.M., D. Gross, and R. Christophersen, *Digital delivery methods of parenting training interventions: a systematic review.* Worldviews on Evidence‐Based Nursing, 2014. **11**(3): p. 168-176.

31. Taylor, T.K., et al., *Computer‐based intervention with coaching: An example using the Incredible Years program.* Cognitive behaviour therapy, 2008. **37**(4): p. 233-246.

32. Rhoades, H., et al., *No digital divide? Technology use among homeless adults.* Journal of Social Distress and the Homeless, 2017. **26**(1): p. 73-77.

33. Rice, E., A. Lee, and S. Taitt, *Cell phone use among homeless youth: potential for new health interventions and research.* Journal of Urban Health, 2011. **88**(6): p. 1175-1182.

34. Freedman, M.J., et al., *Cell phones for ecological momentary assessment with cocaine-addicted homeless patients in treatment.* Journal of substance abuse treatment, 2006. **30**(2): p. 105-111.

35. Tyler, K.A. and R.M. Schmitz, *Using cell phones for data collection: Benefits, outcomes, and intervention possibilities with homeless youth.* Children and Youth Services Review, 2017. **76**: p. 59-64.

36. Griffiths, K.M., et al., *Effect of web-based depression literacy and cognitive–behavioural therapy interventions on stigmatising attitudes to depression: Randomised controlled trial.* The British Journal of Psychiatry, 2004. **185**(4): p. 342-349.

37. Finkelstein, J. and O. Lapshin, *Reducing depression stigma using a web-based program.* International journal of medical informatics, 2007. **76**(10): p. 726-734.

38. Rodda, S., et al., *Web-based counseling for problem gambling: exploring motivations and recommendations.* Journal of medical Internet research, 2013. **15**(5): p. e2474.

39. Grace, R., et al., *Far from ideal: Everyday experiences of mothers and early childhood professionals negotiating an inclusive early childhood experience in the Australian context.* Topics in Early Childhood Special Education, 2008. **28**(1): p. 18-30.

40. McMichael, C. and L. Manderson, *Somali women and well-being: Social networks and social capital among immigrant women in Australia.* Human organization, 2004. **63**(1): p. 88-99.

41. Llewellyn, G., et al., *Support network of mothers with an intellectual disability: An exploratory study.* Journal of Intellectual and Developmental Disability, 1999. **24**(1): p. 7-26.

42. Byrne, F., et al., *Structured social relationships: a review of volunteer home visiting programs for parents of young children.* Australian health review, 2015. **40**(3): p. 262-269.

43. Johnson, Z., F. Howell, and B. Molloy, *Community mothers' programme: randomised controlled trial of non-professional intervention in parenting.* British Medical Journal, 1993. **306**(6890): p. 1449-1452.

For peer review only

44. Deković, M., et al., *Tracing changes in families who participated in the home-start parenting program: Parental sense of competence as mechanism of change.* Prevention science, 2010. **11**(3): p. 263-274.

45. Paisi, M., et al., *Barriers and enablers to accessing dental services for people experiencing homelessness: A systematic review.* Community Dent Oral Epidemiol, 2019. **47**(2): p. 103-111.

46. King, T.B. and G. Gibson, *Oral health needs and access to dental care of homeless adults in the United States: a review.* Spec Care Dentist, 2003. **23**(4): p. 143-7.

47. Stormon, N., et al., *Facilitating Access to Dental Care for People Experiencing Homelessness.* JDR Clinical & Translational Research, 2021. **6**(4): p. 420-429.

48. Goode, J., H. Hoang, and L. Crocombe, *Strategies to improve access to and uptake of dental care by people experiencing homelessness in Australia: a grey literature review.* Aust Health Rev, 2020. **44**(2): p. 297-303.

49. Rigby, M.J., *Potentially over 3 million children in EU Europe believed not to be receiving needed medical and dental treatment-and parents' reasons why.* Child Care Health Dev, 2020. **46**(3): p. 390-396.

50. Dye, B.A., X. Li, and E.D. Beltran-Aguilar, *Selected oral health indicators in the United States, 2005-2008.* NCHS Data Brief, 2012(96): p. 1-8.

51. Daniels, S.R., *The consequences of childhood overweight and obesity.* The future of children, 2006. **16**(1): p. 47-67.

52. Koh, K.A., et al., *The hunger–obesity paradox: obesity in the homeless.* Journal of Urban Health, 2012. **89**(6): p. 952-964.

53. Danielzik, S., et al., *First lessons from the Kiel obesity prevention study (KOPS).* International Journal of Obesity, 2005. **29**(2): p. S78-S83.

54. Nemet, D., D. Geva, and A. Eliakim, *Health promotion intervention in low socioeconomic kindergarten children.* The Journal of pediatrics, 2011. **158**(5): p. 796-801. e1.

55. Muckelbauer, R., et al., *Promotion and provision of drinking water in schools for overweight prevention: randomized, controlled cluster trial.* Pediatrics, 2009. **123**(4): p. e661-e667.

56. Müller, M.J., S. Danielzik, and S. Pust, *School-and family-based interventions to prevent overweight in children.* Proceedings of the Nutrition Society, 2005. **64**(2): p. 249-254.

Scoping Review Protocol

TITLE

Interventions to promote the health and well- being of children under 5s experiencing homelessness in high-income countries: A Scoping Review

PROTOCOL INFORMATION

Authors: All team members
Contact person: Monica Lakhanpaul
Dates: Anticipated start date of search: December 2021
Anticipated completion date: March 2022

For

BACKGROUND

According to the McKinney-Vento definition of homeless, "homeless children and youths" means individuals who lack a fixed, regular, and adequate night-time residence (Miller, 2009). According to the UN Office of the High Commissioner for Human Rights, homelessness has "emerged as a worldwide human rights concern," especially in high-income nation-states with the means to address it. Between 2014 and 2018, family homelessness almost doubled in Ireland, rising from 407 to over 1,600 families. Between 2006 and 2013, New Zealand had a 44% rise in family homelessness. In 2018, the United States had around 56,300 families with children, accounting for one-third of the country's homeless population (Rosenthal DM, 2021). In 2019, the charity Shelter reported that a child loses their home every 8 min in Great Britain, which is the equivalent of 183 children per day. In 2019, the overall number of children who were homeless or in temporary housing in England climbed to 126020, including 88080 in London. According to the Children's Commissioner, there might be around 210000 homeless children in temporary accommodation or couch surfing in England, as well as roughly 585000 people who are either homeless or in danger of becoming homeless.

In particular, young children aged ≤5 years living in temporary accommodation (U5TA) have an inconspicuous plight. There is a lack of policy supporting them since they are not on the streets as homeless. However, many U5TA have pre-existing conditions such as epilepsy, asthma, anxiety, and diabetes; they might be the most susceptible to viral infection (Rosenthal et al., 2020). Moreover, the first five years of life is a critical duration for the optimal development of the brain, especially for children who experience poverty/housing/transient lifestyle, which not only leads to many health concerns but also leads to language development and motor skills and social problems (Cusick and Georgieff, 2016).

Therefore, this scoping review was undertaken to gather existing interventions, especially for families with homeless children within themes: feeding, nutrition, care practice, parenting, dental, wellbeing and mental health, safe sleeping, physical activity, parenting support, which were already effectively conducted in other HICs. Those themes are essential to the integrated health of U5TA, and there is a complex need to include all those themes to address or alleviate the current situation of homelessness. The primary objective of this review is to examine the content and method of delivery of interventions that are culturally sensitive and accessible for the demographic that is on the crossroads of poverty/housing/transient lifestyle. Specifically, our study also aims to address and answer the following question: How best to communicate with mobile populations with poor health literacy/literacy/language barriers? Who are possible and critical points of contact? How to develop an inclusive and engaging practice with

populations in homelessness? How to create appropriate, acceptable, and accessible communication materials? (e.g., through health visitors, family nurse partnerships, etc.)

The scoping review is a core work package of the project titled, “Children in Homeless Accommodations Managing Pandemic Invisibility or Non-inclusive Strategies” to map the existing global evidence on the content and method of delivery of practical strategies and interventions to improve coverage, access, and utilization of early child health programmes in marginalized and excluded under5s.

OBJECTIVES

- O-1. To identify the evidence base available around the content of delivery of interventions programmes for families with children under 5.
- O-2. To explore the method of delivery of these interventions: when to intervene, how to intervene, entry points for crucial health concerns.
- O-3. To find out the most feasible settings in which the strategies and interventions are effective

Specific parameters that will be mapped from studies that relate to the review objectives will comprise:

(i) Challenge area

(ii) Settings of interventions (Content and delivery method)

(iii) Outcome of the interventions

(iv) Culturally accessible/appropriate for mobile populations

(v) Scale of impact

(vi) Economy of impact

METHODS

We will conduct a targeted systematic rapid global scoping review (supplemented by knowledge and lived experiences from international experts) using the Arksey and O’Malley framework (Levac et al., 2010)

1.1. Review Methods

1.1.1. Inclusion Criteria

1.1.1.1. **Language and Geography:** English language studies of HICs;

1.1.1.2. **Types of studies:** Studies of any design, where the study objective was to describe, measure, or evaluate the piloting or implementation of a strategy, tactics, process, and/or method targeted at improving child health programmes in High-income countries;

1.1.1.3. **Types of participants:** Studies that include interventions for under-5 children; The review will look into two qualifiers – marginalized and excluded what? Populations?. Marginalization is defined as “a process whereby something or someone is pushed to the edge of a group and accorded lesser importance.” Social exclusion is defined as “ It involves the lack or denial of resources, rights, goods and services, and the inability to participate **in** the normal relationships and activities, available to the majority of people **in** a society, whether **in** economic, social, cultural or political

For peer review only

arenas." Operationally these two terminologies have been defined to be inclusive of the following keywords:

(Marginal[Title/Abstract])) OR (Marginalised[Title/Abstract])) OR (Marginalized[Title/Abstract])) OR (Refugee[Title/Abstract])) OR (Homeless[Title/Abstract])) OR (Migrant[Title/Abstract])) OR (financial catastrophe[Title/Abstract])) OR (poverty[Title/Abstract])) OR (conflict-affected[Title/Abstract])) OR (Under attack[Title/Abstract])) OR (Displaced[Title/Abstract])) OR (Temporary Accommodation[Title/Abstract])) OR (Temporary Settlement[Title/Abstract])) OR (Temporary Housing[Title/Abstract])) OR (Transitional Settlement[Title/Abstract])) OR (Transitional shelter[Title/Abstract])) OR (Emergency shelter[Title/Abstract])) OR (Emergency accommodation[Title/Abstract])) OR (Emergency housing[Title/Abstract])) OR (Makeshift shelter[Title/Abstract])) OR (Makeshift accommodation[Title/Abstract])) OR (Slash[Title/Abstract] AND Burn Cultivation[Title/Abstract])) OR (Shifting Cultivation[Title/Abstract])) OR (Feral[Title/Abstract])))

1.1.1.4. **Types of intervention:** Studies that will include interventions to improve or optimize the coverage of services, accessibility of the target population, and service utilization; we will include efficacy trials that include interventions only to improve specific health conditions within themes: feeding, nutrition, care practice, parenting, dental, wellbeing, and mental health, safe sleeping, physical activity, parenting support; inclusion of any bridging study will be decided jointly by the lead reviewers and the PI based on any added value)

1.1.1.5. **Types of comparators:** Studies that include marginality, conflict, or inequality for overall outcome, comparison, or sub-grouping.

1.1.1.6. **Types of outcome measures:** Studies that measure service output (coverage, access, utilization) and health (mortality, morbidity, quality-adjusted life years, disease/ disability-adjusted life years) and/or economic (efficiency, cost, return on investment);

1.1.1.7. **Time of Publications:** The dates searched will be from January 2000 to

FINAL SEARCH TERM (See Annexure for More Details)

((((((Child*[Title/Abstract]) OR (((Under five[Title/Abstract]) OR (Under 5[Title/Abstract])) OR (U5[Title/Abstract]))) OR (((Newborn[Title/Abstract]) OR (Infant[Title/Abstract])) OR (1000 days[Title/Abstract]))) AND (((Intervention*[Title/Abstract]) OR (Strateg*[Title/Abstract])) OR (Practice*[Title/Abstract]))) AND ((((((((((((((((((((((((Vulnerable[Title/Abstract]) OR (Marginal[Title/Abstract])) OR (Marginalised[Title/Abstract])) OR (Marginalized[Title/Abstract])) OR (Refugee[Title/Abstract])) OR (Homeless[Title/Abstract])) OR (Migrant[Title/Abstract])) OR (financial catastrophe[Title/Abstract])) OR (poverty[Title/Abstract])) OR (conflict-affected[Title/Abstract])) OR (Under attack[Title/Abstract])) OR (Displaced[Title/Abstract])) OR (Temporary Accommodation[Title/Abstract])) OR (Temporary Settlement[Title/Abstract])) OR (Temporary Housing[Title/Abstract])) OR (Transitional Settlement[Title/Abstract])) OR (Transitional shelter[Title/Abstract])) OR (Emergency shelter[Title/Abstract])) OR (Emergency accommodation[Title/Abstract])) OR (Emergency housing[Title/Abstract])) OR (Makeshift shelter[Title/Abstract])) OR (Makeshift accommodation[Title/Abstract])) OR (Slash[Title/Abstract] AND Burn Cultivation[Title/Abstract])) OR (Shifting Cultivation[Title/Abstract])) OR (Feral[Title/Abstract]))) AND (((((((Access[Title/Abstract]) OR (Accessibility[Title/Abstract])) OR (Inaccessible[Title/Abstract])) OR (inaccess[Title/Abstract])) OR (((coverage[Title/Abstract]) OR (outreach[Title/Abstract])) OR (reach[Title/Abstract]))) OR ((((Use[Title/Abstract]) OR (Usage[Title/Abstract])) OR (Utilisation[Title/Abstract])) OR (Utilization[Title/Abstract]))) OR ((Available[Title/Abstract]) OR (Availability[Title/Abstract])))) AND (((((((Feeding[Title/Abstract]) OR (Nutrition[Title/Abstract])) OR (Care practice[Title/Abstract])) OR (Parenting[Title/Abstract])) OR (((dental[Title/Abstract]) OR (wellbeing[Title/Abstract])) OR (mental health[Title/Abstract]))) OR ((((safe sleeping[Title/Abstract]) OR (physical activity[Title/Abstract])) OR (parenting support[Title/Abstract])) OR (sleep hygiene[Title/Abstract]))) OR ((sleep practice[Title/Abstract]))))) AND (Andorra*[Title/Abstract] OR Antigua and Barbuda*[Title/Abstract] OR Australia*[Title/Abstract] OR Austria*[Title/Abstract] OR Bahrain*[Title/Abstract] OR Barbados*[Title/Abstract] OR Belgium*[Title/Abstract] OR Brunei*[Title/Abstract] OR Canada*[Title/Abstract] OR Chile*[Title/Abstract] OR Croatia*[Title/Abstract] OR Cyprus*[Title/Abstract] OR Czech Republic*[Title/Abstract] OR Denmark*[Title/Abstract] OR Estonia*[Title/Abstract] OR Finland*[Title/Abstract] OR France*[Title/Abstract] OR Germany*[Title/Abstract] OR Greece*[Title/Abstract] OR Hungary*[Title/Abstract] OR Iceland*[Title/Abstract] OR Israel*[Title/Abstract] OR Italy*[Title/Abstract] OR Japan*[Title/Abstract] OR South Korea*[Title/Abstract] OR Kuwait*[Title/Abstract] OR Latvia*[Title/Abstract] OR Liechtenstein*[Title/Abstract] OR Lithuania*[Title/Abstract] OR Luxembourg*[Title/Abstract] OR Monaco*[Title/Abstract] OR Nauru*[Title/Abstract] OR Netherlands*[Title/Abstract] OR New Zealand*[Title/Abstract] OR Norway*[Title/Abstract] OR Oman*[Title/Abstract] OR Palau*[Title/Abstract] OR Poland*[Title/Abstract] OR Portugal*[Title/Abstract] OR Qatar*[Title/Abstract] OR Saint Kitts and Nevis*[Title/Abstract] OR San Marino*[Title/Abstract] OR Seychelles*[Title/Abstract] OR Singapore*[Title/Abstract] OR Slovakia*[Title/Abstract] OR Slovenia*[Title/Abstract] OR Spain*[Title/Abstract] OR Sweden*[Title/Abstract] OR Switzerland*[Title/Abstract] OR Trinidad and Tobago*[Title/Abstract] OR United Arab Emirates*[Title/Abstract] OR United States*[Title/Abstract] OR Aruba*[Title/Abstract] OR Bermuda*[Title/Abstract] OR British Virgin Islands*[Title/Abstract] OR Cayman Islands*[Title/Abstract] OR Cook Islands*[Title/Abstract] OR Curaçao*[Title/Abstract] OR Faroe Islands*[Title/Abstract] OR French Polynesia*[Title/Abstract] OR Gibraltar*[Title/Abstract] OR Greenland*[Title/Abstract] OR Guam*[Title/Abstract] OR Hong Kong*[Title/Abstract] OR Isle of Man*[Title/Abstract] OR New Caledonia*[Title/Abstract] OR Northern Mariana Islands*[Title/Abstract] OR Puerto Rico*[Title/Abstract] OR Saint Martin*[Title/Abstract] OR Taiwan*[Title/Abstract] OR Turks and Caicos Islands*[Title/Abstract] OR U.S. Virgin Islands*[Title/Abstract])AND (("2000/01/01"[Date - Publication] : "3000"[Date - Publication]))

For peer review only - http://bmjopen.bmj.com/site/about/guidelines.xhtml

December 2021.

1.1.2. Search Database

1.1.2.1. **Electronic Database:**

i. Databases: PubMed, MEDLINE, SCOPUS, The Cochrane Library (Cochrane Database of Systematic Reviews, Cochrane Central Register of Controlled Trials [CENTRAL]), and Google Scholar.

ii. Clinical trial registries: ClinicalTrials.gov, Current Controlled Trials meta Register of Controlled Trials, International Clinical Trials Registry Platform (ICTRP) and.

1.1.2.2. **Grey literature:** Conference abstracts, Third Sector Reports.

1.1.2.3. **Correspondence:** Experts from Donor/ Philanthropic organizations and other key experts selected for an interview will be contacted for any additional published or unpublished work.

1.1.3. Relational and Citation Search:

We will conduct snowballing to screen cited articles of the 1st iteration of selections, followed by citations and related articles for each inclusion in PubMed, Google Scholar, and PubMed respectively. A maximum of three iterations will be undertaken.

1.1.4. DATA COLLECTION

1.1.4.1. **Screening Tool and Selection Method:** All articles will be extracted and compiled in a single spreadsheet. The spreadsheet will be equipped with duplicate study filters, and inclusion filters (inclusion criteria not managed through Search Term)

Screening Filters

Geography: High income countries (HICs) (Inclusion)
Types of Emergency: Grade 1, Grade 2, Grade 3 emergencies (WHO),
Age of Target Population: 0-1 (inclusion), 1-5 (inclusion), 5-14 (Exclusion)
Interventions: Interventions on access, coverage, utilization of health services; (inclusion)
Measurements: Output, outcome, impact (inclusion) input, process (exclusion)

and selection columns. Inclusion filters will be filled in independently by one reviewer (YT). Selection filters will be applied independently by two reviewers (YT and KS). All studies selected by two reviewers will be included. All studies rejected by two reviewers will be rejected. All studies having one inclusion will be marked as disputed selection. In case of dispute in selection third expert reviewer (ML) will make the final inclusion decision.

1.1.4.2. **Rounds of Screening:** Two round of screening will be undertaken– Title-Abstract (TiAb) and Full Text (FT).

1.1.4.3. **Preferred Reporting Items for Systematic Reviews and Meta-Analyses (PRISMA) Compliance:** The reasons for exclusion will be recorded and mapped in a flow chart as per PRISMA guidelines.

1.1.4.4. **Data Extraction:** A data extraction form (DEF) will be developed as per CEB Critically Appraised Topics (CAT) guidelines by KS and piloted by two authors (YT and KS) on the first 6 article inclusions (3 each). The piloting will essentially inform whether the DEF can extract necessary and sufficient information as per the objectives set. Any queries raised by the pilot will be reviewed by ML, NS, and ZP. The form will be amended based on the pilot. Two reviewers (YT and KS) will use the finalized form to extract data from full-text articles. If clarification is required, a domain expert (NS, YP, ML) will be consulted.

1.1.5. **Assessment of Risk of Bias (RoB):** RoB assessment will only be used for interventional studies for which results will be used for quantitative analysis or pooling.

For peer review only - http://bmjopen.bmj.com/site/about/guidelines.xhtml

We will use multiple tools for risk of bias assessment based on the study type–

i. Systematic Reviews: **GRADE** for bias due to selective publication and selective non-reporting for systematic reviews
ii. Randomized Trials: **Cochrane RoB 2.0** to assess multiple sources of bias
iii. Non-randomised studies of interventions: **RoBANS** for bias due to selective non-reporting and bias in the selection of the reported result.

1.1.6. **Critical Appraisal for the included studies:** All studies will be critically appraised as per the CAT grading.

1.2. Strategy for data synthesis

The set of evidence will be categorized into qualitative, quantitative and mixed method. The qualitative evidence will be coded to find qualifiers through Word Cloud. The quantitative evidence will be subjected to descriptive analysis, followed by meta-analysis if possible.

1.3. **Referencing Software:** ENDNOTE

1.4. Quality Assessment

https://www.who.int/hrh/retention/annex1_grade_evidence_profiles.pdf

1.5. Method Schema

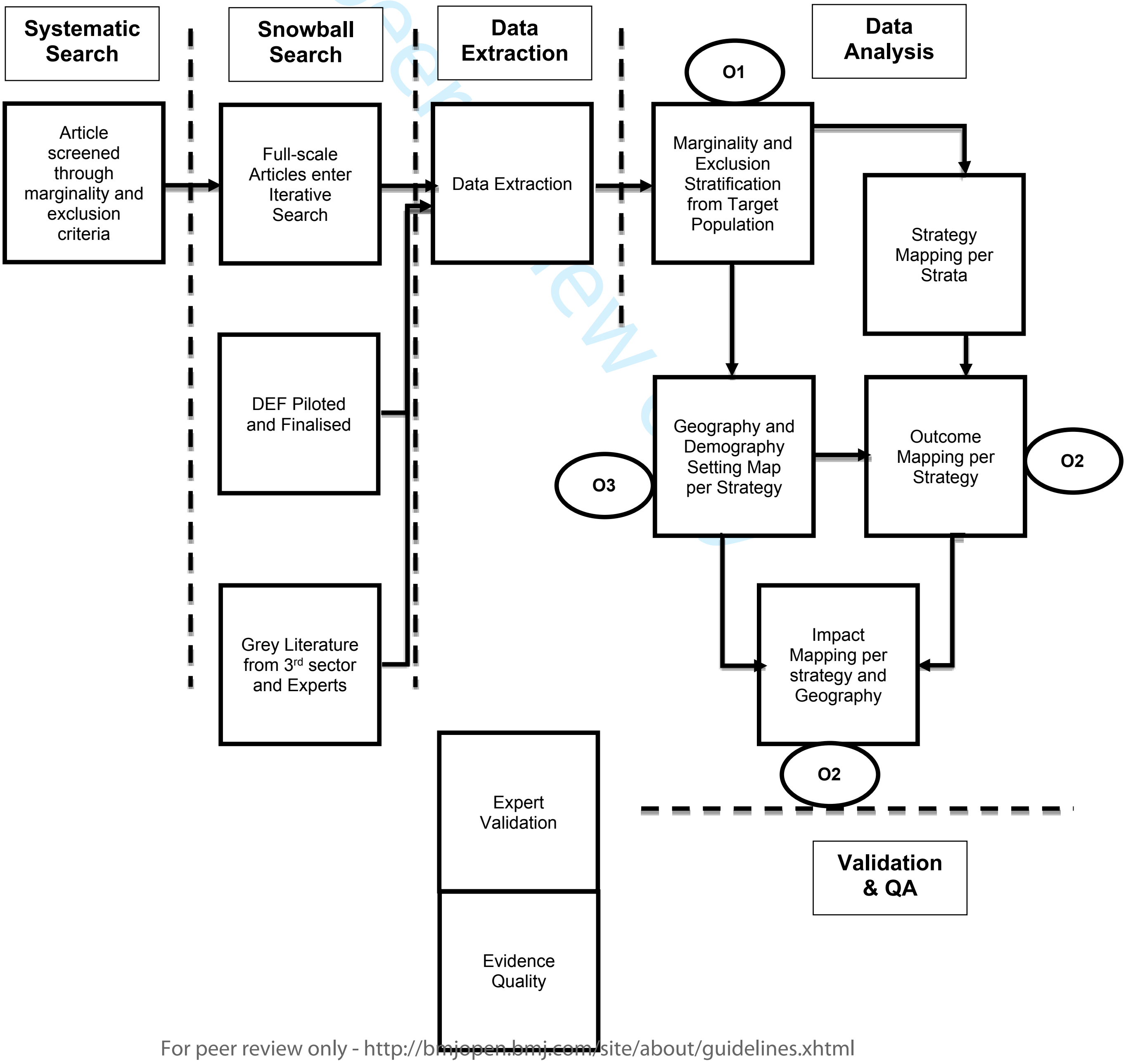

For peer review only

ANNEXURE

Search Strategy

Search number	Query	Sort By	Filters	Search Details	Results	Time
18	(("2000/01/01"[Date - Publication] : "3000/12/31"[Date - Publication])) AND (((((((("child*"[Title/Abstract]) OR ("under five"[Title/Abstract] OR "under 5"[Title/Abstract] OR "U5"[Title/Abstract])) OR ("Newborn"[Title/Abstract] OR "Infant"[Title/Abstract] OR "1000 days"[Title/Abstract])) AND ("intervention*"[Title/Abstract] OR "strategy*"[Title/Abstract] OR "practice*"[Title/Abstract])) AND ("Vulnerable"[Title/Abstract] OR "Marginal"[Title/Abstract] OR "Marginalised"[Title/Abstract] OR "Marginalized"[Title/Abstract] OR "Refugee"[Title/Abstract] OR "Homeless"[Title/Abstract] OR "Migrant"[Title/Abstract] OR "financial catastrophe"[Title/Abstract] OR "poverty"[Title/Abstract] OR "conflict-affected"[Title/Abstract] OR ("Under"[All Fields] OR "attack"[Title/Abstract]) OR "Displaced"[Title/Abstract] OR "temporary accommodation"[Title/Abstract] OR "temporary settlement"[Title/Abstract] OR "temporary housing"[Title/Abstract] OR (("transit"[All Fields] OR "transited"[All Fields] OR "transiting"[All Fields] OR "transition"[All Fields] OR "Transitional"[All Fields] OR "transitionals"[All Fields] OR "transitioned"[All Fields] OR "transitioning"[All Fields] OR "transitions"[All Fields] OR "transits"[All Fields]) OR "Settlement"[Title/Abstract]) OR "transitional shelter"[Title/Abstract] OR "emergency shelter"[Title/Abstract] OR "emergency accommodation"[Title/Abstract] OR "emergency housing"[Title/Abstract] OR ("Makeshift"[All Fields] OR "shelter"[Title/Abstract]) OR ("Makeshift"[All Fields] OR "Accommodation"[Title/Abstract]) OR ("Slash"[Title/Abstract] OR "burn cultivation"[Title/Abstract]) OR "shifting cultivation"[Title/Abstract] OR "Feral"[Title/Abstract])) AND (((("Access"[Title/Abstract] OR "Accessibility"[Title/Abstract] OR "Inaccessible"[Title/Abstract] OR "inaccess"[Title/Abstract]) OR ("coverage"[Title/Abstract] OR "outreach"[Title/Abstract] OR "reach"[Title/Abstract])) OR ("Use"[Title/Abstract] OR "Usage"[Title/Abstract] OR "Utilisation"[Title/Abstract] OR "Utilization"[Title/Abstract])) OR ("Available"[Title/Abstract] OR			("child*"[Title/Abstract] OR ("under five"[Title/Abstract] OR "under 5"[Title/Abstract] OR "U5"[Title/Abstract]) OR ("Newborn"[Title/Abstract] OR "Infant"[Title/Abstract] OR "1000 days"[Title/Abstract])) AND ("intervention*"[Title/Abstract] OR "strategy*"[Title/Abstract] OR "practice*"[Title/Abstract]) AND ("Vulnerable"[Title/Abstract] OR "Marginal"[Title/Abstract] OR "Marginalised"[Title/Abstract] OR "Marginalized"[Title/Abstract] OR "Refugee"[Title/Abstract] OR "Homeless"[Title/Abstract] OR "Migrant"[Title/Abstract] OR "financial catastrophe"[Title/Abstract] OR "poverty"[Title/Abstract] OR "conflict-affected"[Title/Abstract] OR ("Under"[All Fields] AND "attack"[Title/Abstract]) OR "Displaced"[Title/Abstract] OR "temporary accommodation"[Title/Abstract] OR "temporary settlement"[Title/Abstract] OR "temporary housing"[Title/Abstract] OR (("transit"[All Fields] OR "transited"[All Fields] OR "transiting"[All Fields] OR "transition"[All Fields] OR "Transitional"[All Fields] OR "transitionals"[All Fields] OR "transitioned"[All Fields] OR "transitioning"[All Fields] OR "transitions"[All Fields] OR "transits"[All Fields]) AND "Settlement"[Title/Abstract]) OR "transitional shelter"[Title/Abstract] OR "emergency shelter"[Title/Abstract] OR "emergency accommodation"[Title/Abstract] OR "emergency housing"[Title/Abstract] OR ("Makeshift"[All Fields] AND "shelter"[Title/Abstract]) OR ("Makeshift"[All Fields]	523	2:33:18

	"Availability"[Title/Abstract]))) AND ((Feeding*[Title/Abstract]) OR (nutrition*[Title/Abstract]) OR (care practice*[Title/Abstract]) OR (parenting*[Title/Abstract]) OR (dental*[Title/Abstract]) OR (wellbeing*[Title/Abstract]) OR (mental health*[Title/Abstract]) OR (safe sleeping*[Title/Abstract]) OR (physical activity*[Title/Abstract]) OR (parenting support*[Title/Abstract]) OR (sleep hygiene*[Title/Abstract]) OR (physical activity*[Title/Abstract]))) AND (Andorra*[Title/Abstract] OR Antigua and Barbuda*[Title/Abstract] OR Australia*[Title/Abstract] OR Austria*[Title/Abstract] OR Bahrain*[Title/Abstract] OR Barbados*[Title/Abstract] OR Belgium*[Title/Abstract] OR Brunei*[Title/Abstract] OR Canada*[Title/Abstract] OR Chile*[Title/Abstract] OR Croatia*[Title/Abstract] OR Cyprus*[Title/Abstract] OR Czech Republic*[Title/Abstract] OR Denmark*[Title/Abstract] OR Estonia*[Title/Abstract] OR Finland*[Title/Abstract] OR France*[Title/Abstract] OR Germany*[Title/Abstract] OR Greece*[Title/Abstract] OR Hungary*[Title/Abstract] OR Iceland*[Title/Abstract] OR Israel*[Title/Abstract] OR Italy*[Title/Abstract] OR Japan*[Title/Abstract] OR South Korea*[Title/Abstract] OR Kuwait*[Title/Abstract] OR Latvia*[Title/Abstract] OR Liechtenstein*[Title/Abstract] OR Lithuania*[Title/Abstract] OR Luxembourg*[Title/Abstract] OR Monaco*[Title/Abstract] OR Nauru*[Title/Abstract] OR Netherlands*[Title/Abstract] OR New Zealand*[Title/Abstract] OR Norway*[Title/Abstract] OR Oman*[Title/Abstract] OR Palau*[Title/Abstract] OR Poland*[Title/Abstract] OR Portugal*[Title/Abstract] OR Qatar*[Title/Abstract] OR Saint Kitts and Nevis*[Title/Abstract] OR San Marino*[Title/Abstract] OR Seychelles*[Title/Abstract] OR Singapore*[Title/Abstract] OR Slovakia*[Title/Abstract] OR Slovenia*[Title/Abstract] OR Spain*[Title/Abstract] OR Sweden*[Title/Abstract] OR Switzerland*[Title/Abstract] OR Trinidad and Tobago*[Title/Abstract] OR United Arab Emirates*[Title/Abstract] OR United States*[Title/Abstract] OR Aruba*[Title/Abstract] OR Bermuda*[Title/Abstract] OR British Virgin Islands*[Title/Abstract] OR Cayman Islands*[Title/Abstract] OR Cook Islands*[Title/Abstract] OR Curaçao*[Title/Abstract] OR Faroe Islands*[Title/Abstract] OR French Polynesia*[Title/Abstract] OR			AND "Accommodation"[Title/Abstract]) OR ("Slash"[Title/Abstract] AND "burn cultivation"[Title/Abstract]) OR "shifting cultivation"[Title/Abstract] OR "Feral"[Title/Abstract]) AND ("Access"[Title/Abstract] OR "Accessibility"[Title/Abstract] OR "Inaccessible"[Title/Abstract] OR "inaccess"[Title/Abstract] OR ("coverage"[Title/Abstract] OR "outreach"[Title/Abstract] OR "reach"[Title/Abstract]) OR ("Use"[Title/Abstract] OR "Usage"[Title/Abstract] OR "Utilisation"[Title/Abstract] OR "Utilization"[Title/Abstract]) OR ("Available"[Title/Abstract] OR "Availability"[Title/Abstract])) AND (Feeding*[Title/Abstract])) OR (nutrition*[Title/Abstract])) OR (care practice*[Title/Abstract])) OR (parenting*[Title/Abstract])) OR (dental*[Title/Abstract])) OR (wellbeing*[Title/Abstract])) OR (mental health*[Title/Abstract])) OR (safe sleeping*[Title/Abstract])) OR (physical activity*[Title/Abstract])) OR (parenting support*[Title/Abstract])) OR (sleep hygiene*[Title/Abstract])) OR (physical activity*[Title/Abstract]) AND (Andorra*[Title/Abstract] OR Antigua and Barbuda*[Title/Abstract] OR Australia*[Title/Abstract] OR Austria*[Title/Abstract] OR Bahrain*[Title/Abstract] OR Barbados*[Title/Abstract] OR Belgium*[Title/Abstract] OR Brunei*[Title/Abstract] OR Canada*[Title/Abstract] OR Chile*[Title/Abstract] OR Croatia*[Title/Abstract] OR Cyprus*[Title/Abstract] OR Czech Republic*[Title/Abstract] OR			

For peer review only

	Gibraltar*[Title/Abstract] OR Greenland*[Title/Abstract] OR Guam*[Title/Abstract] OR Hong Kong*[Title/Abstract] OR Isle of Man*[Title/Abstract] OR New Caledonia*[Title/Abstract] OR Northern Mariana Islands*[Title/Abstract] OR Puerto Rico*[Title/Abstract] OR Saint Martin*[Title/Abstract] OR Taiwan*[Title/Abstract] OR Turks and Caicos Islands*[Title/Abstract] OR U.S. Virgin Islands*[Title/Abstract]))			Denmark*[Title/Abstract] OR Estonia*[Title/Abstract] OR Finland*[Title/Abstract] OR France*[Title/Abstract] OR Germany*[Title/Abstract] OR Greece*[Title/Abstract] OR Hungary*[Title/Abstract] OR Iceland*[Title/Abstract] OR Israel*[Title/Abstract] OR Italy*[Title/Abstract] OR Japan*[Title/Abstract] OR South Korea*[Title/Abstract] OR Kuwait*[Title/Abstract] OR Latvia*[Title/Abstract] OR Liechtenstein*[Title/Abstract] OR Lithuania*[Title/Abstract] OR Luxembourg*[Title/Abstract] OR Monaco*[Title/Abstract] OR Nauru*[Title/Abstract] OR Netherlands*[Title/Abstract] OR New Zealand*[Title/Abstract] OR Norway*[Title/Abstract] OR Oman*[Title/Abstract] OR Palau*[Title/Abstract] OR Poland*[Title/Abstract] OR Portugal*[Title/Abstract] OR Qatar*[Title/Abstract] OR Saint Kitts and Nevis*[Title/Abstract] OR San Marino*[Title/Abstract] OR Seychelles*[Title/Abstract] OR Singapore*[Title/Abstract] OR Slovakia*[Title/Abstract] OR Slovenia*[Title/Abstract] OR Spain*[Title/Abstract] OR Sweden*[Title/Abstract] OR Switzerland*[Title/Abstract] OR Trinidad and Tobago*[Title/Abstract] OR United Arab Emirates*[Title/Abstract] OR United States*[Title/Abstract] OR Aruba*[Title/Abstract] OR Bermuda*[Title/Abstract] OR British Virgin Islands*[Title/Abstract] OR Cayman Islands*[Title/Abstract] OR Cook Islands*[Title/Abstract] OR Curaçao*[Title/Abstract] OR Faroe Islands*[Title/Abstract] OR French Polynesia*[Title/Abstract] OR Gibraltar*[Title/Abstract] OR Greenland*[Title/Abstract] OR Guam*[Title/Abstract] OR Hong Kong*[Title/Abstract] OR		

				Isle of Man*[Title/Abstract] OR New Caledonia*[Title/Abstract] OR Northern Mariana Islands*[Title/Abstract] OR Puerto Rico*[Title/Abstract] OR Saint Martin*[Title/Abstract] OR Taiwan*[Title/Abstract] OR Turks and Caicos Islands*[Title/Abstract] OR U.S. Virgin Islands*[Title/Abstract]))AND 2010/01/01:3000/12/31[Date - Publication]		

For peer review only

17	((((((("child*"[Title/Abstract]) OR ("under five"[Title/Abstract] OR "under 5"[Title/Abstract] OR "U5"[Title/Abstract])) OR ("Newborn"[Title/Abstract] OR "Infant"[Title/Abstract] OR "1000 days"[Title/Abstract])) AND ("intervention*"[Title/Abstract] OR "strategy*"[Title/Abstract] OR "practice*"[Title/Abstract])) AND ("Vulnerable"[Title/Abstract] OR "Marginal"[Title/Abstract] OR "Marginalised"[Title/Abstract] OR "Marginalized"[Title/Abstract] OR "Refugee"[Title/Abstract] OR "Homeless"[Title/Abstract] OR "Migrant"[Title/Abstract] OR "financial catastrophe"[Title/Abstract] OR "poverty"[Title/Abstract] OR "conflict-affected"[Title/Abstract] OR ("Under"[All Fields] OR "attack"[Title/Abstract]) OR "Displaced"[Title/Abstract] OR "temporary accommodation"[Title/Abstract] OR "temporary settlement"[Title/Abstract] OR "temporary housing"[Title/Abstract] OR (("transit"[All Fields] OR "transited"[All Fields] OR "transiting"[All Fields] OR "transition"[All Fields] OR "Transitional"[All Fields] OR "transitionals"[All Fields] OR "transitioned"[All Fields] OR "transitioning"[All Fields] OR "transitions"[All Fields] OR "transits"[All Fields]) OR "Settlement"[Title/Abstract]) OR "transitional shelter"[Title/Abstract] OR "emergency shelter"[Title/Abstract] OR "emergency accommodation"[Title/Abstract] OR "emergency housing"[Title/Abstract] OR ("Makeshift"[All Fields] OR "shelter"[Title/Abstract]) OR ("Makeshift"[All Fields] OR "Accommodation"[Title/Abstract]) OR ("Slash"[Title/Abstract] OR "burn cultivation"[Title/Abstract]) OR "shifting cultivation"[Title/Abstract] OR "Feral"[Title/Abstract)) AND (((("Access"[Title/Abstract] OR "Accessibility"[Title/Abstract] OR "Inaccessible"[Title/Abstract] OR "inaccess"[Title/Abstract]) OR ("coverage"[Title/Abstract] OR "outreach"[Title/Abstract] OR "reach"[Title/Abstract])) OR ("Use"[Title/Abstract] OR "Usage"[Title/Abstract] OR "Utilisation"[Title/Abstract] OR "Utilization"[Title/Abstract])) OR ("Available"[Title/Abstract] OR "Availability"[Title/Abstract]))) AND ((Feeding*[Title/Abstract]) OR (nutrition*[Title/Abstract]) OR (care practice*[Title/Abstract]) OR (parenting*[Title/Abstract]) OR (dental*[Title/Abstract]) OR (wellbeing*[Title/Abstract]) OR (mental health*[Title/Abstract]) OR (safe sleeping*[Title/Abstract]) OR (physical activity*[Title/Abstract]) OR			(("child*"[Title/Abstract] OR ("under five"[Title/Abstract] OR "under 5"[Title/Abstract] OR "U5"[Title/Abstract]) OR ("Newborn"[Title/Abstract] OR "Infant"[Title/Abstract] OR "1000 days"[Title/Abstract])) AND ("intervention*"[Title/Abstract] OR "strategy*"[Title/Abstract] OR "practice*"[Title/Abstract]) AND ("Vulnerable"[Title/Abstract] OR "Marginal"[Title/Abstract] OR "Marginalised"[Title/Abstract] OR "Marginalized"[Title/Abstract] OR "Refugee"[Title/Abstract] OR "Homeless"[Title/Abstract] OR "Migrant"[Title/Abstract] OR "financial catastrophe"[Title/Abstract] OR "poverty"[Title/Abstract] OR "conflict-affected"[Title/Abstract] OR ("Under"[All Fields] AND "attack"[Title/Abstract]) OR "Displaced"[Title/Abstract] OR "temporary accommodation"[Title/Abstract] OR "temporary settlement"[Title/Abstract] OR "temporary housing"[Title/Abstract] OR (("transit"[All Fields] OR "transited"[All Fields] OR "transiting"[All Fields] OR "transition"[All Fields] OR "Transitional"[All Fields] OR "transitionals"[All Fields] OR "transitioned"[All Fields] OR "transitioning"[All Fields] OR "transitions"[All Fields] OR "transits"[All Fields]) AND "Settlement"[Title/Abstract]) OR "transitional shelter"[Title/Abstract] OR "emergency shelter"[Title/Abstract] OR "emergency accommodation"[Title/Abstract] OR "emergency housing"[Title/Abstract] OR ("Makeshift"[All Fields] AND "shelter"[Title/Abstract]) OR ("Makeshift"[All Fields] AND "Accommodation"[Title/Abstract]) OR ("Slash"[Title/Abstract] AND "burn cultivation"[Title/Abstract]) OR "shifting cultivation"[Title/Abstract]	642	2:32:48

For peer review only

	(parenting support*[Title/Abstract]) OR (sleep hygiene*[Title/Abstract]) OR (physical activity*[Title/Abstract]))) AND (Andorra*[Title/Abstract] OR Antigua and Barbuda*[Title/Abstract] OR Australia*[Title/Abstract] OR Austria*[Title/Abstract] OR Bahrain*[Title/Abstract] OR Barbados*[Title/Abstract] OR Belgium*[Title/Abstract] OR Brunei*[Title/Abstract] OR Canada*[Title/Abstract] OR Chile*[Title/Abstract] OR Croatia*[Title/Abstract] OR Cyprus*[Title/Abstract] OR Czech Republic*[Title/Abstract] OR Denmark*[Title/Abstract] OR Estonia*[Title/Abstract] OR Finland*[Title/Abstract] OR France*[Title/Abstract] OR Germany*[Title/Abstract] OR Greece*[Title/Abstract] OR Hungary*[Title/Abstract] OR Iceland*[Title/Abstract] OR Israel*[Title/Abstract] OR Italy*[Title/Abstract] OR Japan*[Title/Abstract] OR South Korea*[Title/Abstract] OR Kuwait*[Title/Abstract] OR Latvia*[Title/Abstract] OR Liechtenstein*[Title/Abstract] OR Lithuania*[Title/Abstract] OR Luxembourg*[Title/Abstract] OR Monaco*[Title/Abstract] OR Nauru*[Title/Abstract] OR Netherlands*[Title/Abstract] OR New Zealand*[Title/Abstract] OR Norway*[Title/Abstract] OR Oman*[Title/Abstract] OR Palau*[Title/Abstract] OR Poland*[Title/Abstract] OR Portugal*[Title/Abstract] OR Qatar*[Title/Abstract] OR Saint Kitts and Nevis*[Title/Abstract] OR San Marino*[Title/Abstract] OR Seychelles*[Title/Abstract] OR Singapore*[Title/Abstract] OR Slovakia*[Title/Abstract] OR Slovenia*[Title/Abstract] OR Spain*[Title/Abstract] OR Sweden*[Title/Abstract] OR Switzerland*[Title/Abstract] OR Trinidad and Tobago*[Title/Abstract] OR United Arab Emirates*[Title/Abstract] OR United States*[Title/Abstract] OR Aruba*[Title/Abstract] OR Bermuda*[Title/Abstract] OR British Virgin Islands*[Title/Abstract] OR Cayman Islands*[Title/Abstract] OR Cook Islands*[Title/Abstract] OR Curaçao*[Title/Abstract] OR Faroe Islands*[Title/Abstract] OR French Polynesia*[Title/Abstract] OR Gibraltar*[Title/Abstract] OR Greenland*[Title/Abstract] OR Guam*[Title/Abstract] OR Hong Kong*[Title/Abstract] OR Isle of Man*[Title/Abstract] OR New Caledonia*[Title/Abstract] OR Northern Mariana Islands*[Title/Abstract] OR Puerto Rico*[Title/Abstract] OR Saint Martin*[Title/Abstract] OR			OR "Feral"[Title/Abstract]) AND ("Access"[Title/Abstract] OR "Accessibility"[Title/Abstract] OR "Inaccessible"[Title/Abstract] OR "inaccess"[Title/Abstract] OR ("coverage"[Title/Abstract] OR "outreach"[Title/Abstract] OR "reach"[Title/Abstract]) OR ("Use"[Title/Abstract] OR "Usage"[Title/Abstract] OR "Utilisation"[Title/Abstract] OR "Utilization"[Title/Abstract]) OR ("Available"[Title/Abstract] OR "Availability"[Title/Abstract]))) AND (Feeding*[Title/Abstract])) OR (nutrition*[Title/Abstract])) OR (care practice*[Title/Abstract])) OR (parenting*[Title/Abstract])) OR (dental*[Title/Abstract])) OR (wellbeing*[Title/Abstract])) OR (mental health*[Title/Abstract])) OR (safe sleeping*[Title/Abstract])) OR (physical activity*[Title/Abstract])) OR (parenting support*[Title/Abstract])) OR (sleep hygiene*[Title/Abstract])) OR (physical activity*[Title/Abstract]) AND (Andorra*[Title/Abstract] OR Antigua and Barbuda*[Title/Abstract] OR Australia*[Title/Abstract] OR Austria*[Title/Abstract] OR Bahrain*[Title/Abstract] OR Barbados*[Title/Abstract] OR Belgium*[Title/Abstract] OR Brunei*[Title/Abstract] OR Canada*[Title/Abstract] OR Chile*[Title/Abstract] OR Croatia*[Title/Abstract] OR Cyprus*[Title/Abstract] OR Czech Republic*[Title/Abstract] OR Denmark*[Title/Abstract] OR Estonia*[Title/Abstract] OR Finland*[Title/Abstract] OR France*[Title/Abstract] OR Germany*[Title/Abstract] OR Greece*[Title/Abstract] OR		

	Taiwan*[Title/Abstract] OR Turks and Caicos Islands*[Title/Abstract] OR U.S. Virgin Islands*[Title/Abstract])			Hungary*[Title/Abstract] OR Iceland*[Title/Abstract] OR Israel*[Title/Abstract] OR Italy*[Title/Abstract] OR Japan*[Title/Abstract] OR South Korea*[Title/Abstract] OR Kuwait*[Title/Abstract] OR Latvia*[Title/Abstract] OR Liechtenstein*[Title/Abstract] OR Lithuania*[Title/Abstract] OR Luxembourg*[Title/Abstract] OR Monaco*[Title/Abstract] OR Nauru*[Title/Abstract] OR Netherlands*[Title/Abstract] OR New Zealand*[Title/Abstract] OR Norway*[Title/Abstract] OR Oman*[Title/Abstract] OR Palau*[Title/Abstract] OR Poland*[Title/Abstract] OR Portugal*[Title/Abstract] OR Qatar*[Title/Abstract] OR Saint Kitts and Nevis*[Title/Abstract] OR San Marino*[Title/Abstract] OR Seychelles*[Title/Abstract] OR Singapore*[Title/Abstract] OR Slovakia*[Title/Abstract] OR Slovenia*[Title/Abstract] OR Spain*[Title/Abstract] OR Sweden*[Title/Abstract] OR Switzerland*[Title/Abstract] OR Trinidad and Tobago*[Title/Abstract] OR United Arab Emirates*[Title/Abstract] OR United States*[Title/Abstract] OR Aruba*[Title/Abstract] OR Bermuda*[Title/Abstract] OR British Virgin Islands*[Title/Abstract] OR Cayman Islands*[Title/Abstract] OR Cook Islands*[Title/Abstract] OR Curaçao*[Title/Abstract] OR Faroe Islands*[Title/Abstract] OR French Polynesia*[Title/Abstract] OR Gibraltar*[Title/Abstract] OR Greenland*[Title/Abstract] OR Guam*[Title/Abstract] OR Hong Kong*[Title/Abstract] OR Isle of Man*[Title/Abstract] OR New Caledonia*[Title/Abstract] OR Northern Mariana Islands*[Title/Abstract] OR Puerto Rico*[Title/Abstract] OR Saint Martin*[Title/Abstract] OR		

				Taiwan*[Title/Abstract] OR Turks and Caicos Islands*[Title/Abstract] OR U.S. Virgin Islands*[Title/Abstract]))		

For peer review only

16	(((((Child*[Title/Abstract]) OR (((Under five[Title/Abstract]) OR (Under 5[Title/Abstract])) OR (U5[Title/Abstract]))) OR (((Newborn[Title/Abstract]) OR (Infant[Title/Abstract])) OR (1000 days[Title/Abstract]))) AND (((Intervention*[Title/Abstract]) OR (Strateg*[Title/Abstract])) OR (Practice*[Title/Abstract]))) AND (((((((((((((((((((((((((Vulnerable[Title/Abstract]) OR (Marginal[Title/Abstract])) OR (Marginalised[Title/Abstract])) OR (Marginalized[Title/Abstract])) OR (Refugee[Title/Abstract])) OR (Homeless[Title/Abstract])) OR (Migrant[Title/Abstract])) OR (financial catastrophe[Title/Abstract])) OR (poverty[Title/Abstract])) OR (conflict-affected[Title/Abstract])) OR (Under attack[Title/Abstract])) OR (Displaced[Title/Abstract])) OR (Temporary Accommodation[Title/Abstract])) OR (Temporary Settlement[Title/Abstract])) OR (Temporary Housing[Title/Abstract])) OR (Transitional Settlement[Title/Abstract])) OR (Transitional shelter[Title/Abstract])) OR (Emergency shelter[Title/Abstract])) OR (Emergency accommodation[Title/Abstract])) OR (Emergency housing[Title/Abstract])) OR (Makeshift shelter[Title/Abstract])) OR (Makeshift accommodation[Title/Abstract])) OR (Slash[Title/Abstract] AND Burn Cultivation[Title/Abstract])) OR (Shifting Cultivation[Title/Abstract])) OR (Feral[Title/Abstract]))) AND (((((((Access[Title/Abstract]) OR (Accessibility[Title/Abstract])) OR (Inaccessible[Title/Abstract])) OR (inaccess[Title/Abstract])) OR (((coverage[Title/Abstract]) OR (outreach[Title/Abstract])) OR (reach[Title/Abstract]))) OR ((((Use[Title/Abstract]) OR (Usage[Title/Abstract])) OR (Utilisation[Title/Abstract])) OR (Utilization[Title/Abstract]))) OR ((Available[Title/Abstract]) OR (Availability[Title/Abstract]))) AND (((((((Feeding[Title/Abstract]) OR (Nutrition[Title/Abstract])) OR (Care practice[Title/Abstract])) OR (Parenting[Title/Abstract])) OR (((dental[Title/Abstract]) OR (wellbeing[Title/Abstract])) OR (mental health[Title/Abstract]))) OR ((((safe sleeping[Title/Abstract]) OR (physical activity[Title/Abstract])) OR (parenting support[Title/Abstract])) OR (sleep hygiene[Title/Abstract]))) OR ((sleep practice[Title/Abstract])))))			("child*"[Title/Abstract] OR ("under five"[Title/Abstract] OR "under 5"[Title/Abstract] OR "U5"[Title/Abstract]) OR ("Newborn"[Title/Abstract] OR "Infant"[Title/Abstract] OR "1000 days"[Title/Abstract])) AND ("intervention*"[Title/Abstract] OR "strategy*"[Title/Abstract] OR "practice*"[Title/Abstract]) AND ("Vulnerable"[Title/Abstract] OR "Marginal"[Title/Abstract] OR "Marginalised"[Title/Abstract] OR "Marginalized"[Title/Abstract] OR "Refugee"[Title/Abstract] OR "Homeless"[Title/Abstract] OR "Migrant"[Title/Abstract] OR "financial catastrophe"[Title/Abstract] OR "poverty"[Title/Abstract] OR "conflict-affected"[Title/Abstract] OR ("Under"[All Fields] AND "attack"[Title/Abstract]) OR "Displaced"[Title/Abstract] OR "temporary accommodation"[Title/Abstract] OR "temporary settlement"[Title/Abstract] OR "temporary housing"[Title/Abstract] OR (("transit"[All Fields] OR "transited"[All Fields] OR "transiting"[All Fields] OR "transition"[All Fields] OR "Transitional"[All Fields] OR "transitionals"[All Fields] OR "transitioned"[All Fields] OR "transitioning"[All Fields] OR "transitions"[All Fields] OR "transits"[All Fields]) AND "Settlement"[Title/Abstract]) OR "transitional shelter"[Title/Abstract] OR "emergency shelter"[Title/Abstract] OR "emergency accommodation"[Title/Abstract] OR "emergency housing"[Title/Abstract] OR ("Makeshift"[All Fields] AND "shelter"[Title/Abstract]) OR ("Makeshift"[All Fields] AND "Accommodation"[Title/Abstract]) OR ("Slash"[Title/Abstract] AND "burn cultivation"[Title/Abstract]) OR "shifting cultivation"[Title/Abstract]		

For peer review only

				OR "Feral"[Title/Abstract]) AND ("Access"[Title/Abstract] OR "Accessibility"[Title/Abstract] OR "Inaccessible"[Title/Abstract] OR "inaccess"[Title/Abstract] OR ("coverage"[Title/Abstract] OR "outreach"[Title/Abstract] OR "reach"[Title/Abstract]) OR ("Use"[Title/Abstract] OR "Usage"[Title/Abstract] OR "Utilisation"[Title/Abstract] OR "Utilization"[Title/Abstract]) OR ("Available"[Title/Abstract] OR "Availability"[Title/Abstract])) AND (Feeding*[Title/Abstract])) OR (nutrition*[Title/Abstract])) OR (care practice*[Title/Abstract])) OR (parenting*[Title/Abstract])) OR (dental*[Title/Abstract])) OR (wellbeing*[Title/Abstract])) OR (mental health*[Title/Abstract])) OR (safe sleeping*[Title/Abstract])) OR (physical activity*[Title/Abstract])) OR (parenting support*[Title/Abstract])) OR (sleep hygiene*[Title/Abstract])) OR (physical activity*[Title/Abstract])		

For peer review only

15	(((((Child*[Title/Abstract]) OR (((Under five[Title/Abstract]) OR (Under 5[Title/Abstract])) OR (U5[Title/Abstract]))) OR (((Newborn[Title/Abstract]) OR (Infant[Title/Abstract])) OR (1000 days[Title/Abstract]))) AND (((Intervention*[Title/Abstract]) OR (Strateg*[Title/Abstract])) OR (Practice*[Title/Abstract]))) AND ((((((((((((((((((((((((Vulnerable[Title/Abstract]) OR (Marginal[Title/Abstract])) OR (Marginalised[Title/Abstract])) OR (Marginalized[Title/Abstract])) OR (Refugee[Title/Abstract])) OR (Homeless[Title/Abstract])) OR (Migrant[Title/Abstract])) OR (financial catastrophe[Title/Abstract])) OR (poverty[Title/Abstract])) OR (conflict-affected[Title/Abstract])) OR (Under attack[Title/Abstract])) OR (Displaced[Title/Abstract])) OR (Temporary Accommodation[Title/Abstract])) OR (Temporary Settlement[Title/Abstract])) OR (Temporary Housing[Title/Abstract])) OR (Transitional Settlement[Title/Abstract])) OR (Transitional shelter[Title/Abstract])) OR (Emergency shelter[Title/Abstract])) OR (Emergency accommodation[Title/Abstract])) OR (Emergency housing[Title/Abstract])) OR (Makeshift shelter[Title/Abstract])) OR (Makeshift accommodation[Title/Abstract])) OR (Slash[Title/Abstract] AND Burn Cultivation[Title/Abstract])) OR (Shifting Cultivation[Title/Abstract])) OR (Feral[Title/Abstract]))) AND (((((((Access[Title/Abstract]) OR (Accessibility[Title/Abstract])) OR (Inaccessible[Title/Abstract])) OR (inaccess[Title/Abstract])) OR (((coverage[Title/Abstract]) OR (outreach[Title/Abstract])) OR (reach[Title/Abstract]))) OR ((((Use[Title/Abstract]) OR (Usage[Title/Abstract])) OR (Utilisation[Title/Abstract])) OR (Utilization[Title/Abstract]))) OR ((Available[Title/Abstract]) OR (Availability[Title/Abstract])))			("child*"[Title/Abstract] OR ("under five"[Title/Abstract] OR "under 5"[Title/Abstract] OR "U5"[Title/Abstract]) OR ("Newborn"[Title/Abstract] OR "Infant"[Title/Abstract] OR "1000 days"[Title/Abstract])) AND ("intervention*"[Title/Abstract] OR "strategy*"[Title/Abstract] OR "practice*"[Title/Abstract]) AND ("Vulnerable"[Title/Abstract] OR "Marginal"[Title/Abstract] OR "Marginalised"[Title/Abstract] OR "Marginalized"[Title/Abstract] OR "Refugee"[Title/Abstract] OR "Homeless"[Title/Abstract] OR "Migrant"[Title/Abstract] OR "financial catastrophe"[Title/Abstract] OR "poverty"[Title/Abstract] OR "conflict-affected"[Title/Abstract] OR ("Under"[All Fields] AND "attack"[Title/Abstract]) OR "Displaced"[Title/Abstract] OR "temporary accommodation"[Title/Abstract] OR "temporary settlement"[Title/Abstract] OR "temporary housing"[Title/Abstract] OR (("transit"[All Fields] OR "transited"[All Fields] OR "transiting"[All Fields] OR "transition"[All Fields] OR "Transitional"[All Fields] OR "transitionals"[All Fields] OR "transitioned"[All Fields] OR "transitioning"[All Fields] OR "transitions"[All Fields] OR "transits"[All Fields]) AND "Settlement"[Title/Abstract]) OR "transitional shelter"[Title/Abstract] OR "emergency shelter"[Title/Abstract] OR "emergency accommodation"[Title/Abstract] OR "emergency housing"[Title/Abstract] OR ("Makeshift"[All Fields] AND "shelter"[Title/Abstract]) OR ("Makeshift"[All Fields] AND "Accommodation"[Title/Abstract]) OR ("Slash"[Title/Abstract] AND "burn cultivation"[Title/Abstract]) OR "shifting cultivation"[Title/Abstract]	14,828	2:31:46

				OR "Feral"[Title/Abstract]) AND ("Access"[Title/Abstract] OR "Accessibility"[Title/Abstract] OR "Inaccessible"[Title/Abstract] OR "inaccess"[Title/Abstract] OR ("coverage"[Title/Abstract] OR "outreach"[Title/Abstract] OR "reach"[Title/Abstract]) OR ("Use"[Title/Abstract] OR "Usage"[Title/Abstract] OR "Utilisation"[Title/Abstract] OR "Utilization"[Title/Abstract]) OR ("Available"[Title/Abstract] OR "Availability"[Title/Abstract]))		
14	((((((Access[Title/Abstract]) OR (Accessibility[Title/Abstract])) OR (Inaccessible[Title/Abstract])) OR (inaccess[Title/Abstract])) OR (((coverage[Title/Abstract]) OR (outreach[Title/Abstract])) OR (reach[Title/Abstract]))) OR ((((Use[Title/Abstract]) OR (Usage[Title/Abstract])) OR (Utilisation[Title/Abstract])) OR (Utilization[Title/Abstract]))) OR ((Available[Title/Abstract]) OR (Availability[Title/Abstract]))			"Access"[Title/Abstract] OR "Accessibility"[Title/Abstract] OR "Inaccessible"[Title/Abstract] OR "inaccess"[Title/Abstract] OR "coverage"[Title/Abstract] OR "outreach"[Title/Abstract] OR "reach"[Title/Abstract] OR "Use"[Title/Abstract] OR "Usage"[Title/Abstract] OR "Utilisation"[Title/Abstract] OR "Utilization"[Title/Abstract] OR "Available"[Title/Abstract] OR "Availability"[Title/Abstract]	4,907,175	2:31:24
13	(Available[Title/Abstract]) OR (Availability[Title/Abstract])			"Available"[Title/Abstract] OR "Availability"[Title/Abstract]	1,358,789	2:30:57

12	(((Use[Title/Abstract]) OR (Usage[Title/Abstract])) OR (Utilisation[Title/Abstract])) OR (Utilization[Title/Abstract])			"Use"[Title/Abstract] OR "Usage"[Title/Abstract] OR "Utilisation"[Title/Abstract] OR "Utilization"[Title/Abstract]	3,385,414	2:30:36
11	((coverage[Title/Abstract]) OR (outreach[Title/Abstract])) OR (reach[Title/Abstract])			"coverage"[Title/Abstract] OR "outreach"[Title/Abstract] OR "reach"[Title/Abstract]	325,002	2:28:26
10	(((Access[Title/Abstract]) OR (Accessibility[Title/Abstract])) OR (Inaccessible[Title/Abstract])) OR (inaccess[Title/Abstract])			"Access"[Title/Abstract] OR "Accessibility"[Title/Abstract] OR "Inaccessible"[Title/Abstract] OR "inaccess"[Title/Abstract]	424,084	2:27:31
9	((((Child*[Title/Abstract]) OR (((Under five[Title/Abstract]) OR (Under 5[Title/Abstract])) OR (U5[Title/Abstract]))) OR (((Newborn[Title/Abstract]) OR (Infant[Title/Abstract])) OR (1000 days[Title/Abstract]))) AND (((Intervention*[Title/Abstract]) OR (Strateg*[Title/Abstract])) OR (Practice*[Title/Abstract]))) AND ((((((((((((((((((((((((Vulnerable[Title/Abstract]) OR (Marginal[Title/Abstract])) OR (Marginalised[Title/Abstract])) OR (Marginalized[Title/Abstract])) OR (Refugee[Title/Abstract])) OR (Homeless[Title/Abstract])) OR (Migrant[Title/Abstract])) OR (financial catastrophe[Title/Abstract])) OR (poverty[Title/Abstract])) OR (conflict-affected[Title/Abstract])) OR (Under attack[Title/Abstract])) OR (Displaced[Title/Abstract])) OR (Temporary Accommodation[Title/Abstract])) OR (Temporary Settlement[Title/Abstract])) OR (Temporary Housing[Title/Abstract])) OR (Transitional Settlement[Title/Abstract])) OR (Transitional shelter[Title/Abstract])) OR (Emergency shelter[Title/Abstract])) OR (Emergency accommodation[Title/Abstract])) OR (Emergency housing[Title/Abstract])) OR (Makeshift shelter[Title/Abstract])) OR (Makeshift accommodation[Title/Abstract])) OR (Slash[Title/Abstract] AND Burn Cultivation[Title/Abstract])) OR (Shifting Cultivation[Title/Abstract])) OR (Feral[Title/Abstract]))			("child*"[Title/Abstract] OR ("under five"[Title/Abstract] OR "under 5"[Title/Abstract] OR "U5"[Title/Abstract]) OR ("Newborn"[Title/Abstract] OR "Infant"[Title/Abstract] OR "1000 days"[Title/Abstract])) AND ("intervention*"[Title/Abstract] OR "strategy*"[Title/Abstract] OR "practice*"[Title/Abstract]) AND ("Vulnerable"[Title/Abstract] OR "Marginal"[Title/Abstract] OR "Marginalised"[Title/Abstract] OR "Marginalized"[Title/Abstract] OR "Refugee"[Title/Abstract] OR "Homeless"[Title/Abstract] OR "Migrant"[Title/Abstract] OR "financial catastrophe"[Title/Abstract] OR "poverty"[Title/Abstract] OR "conflict-affected"[Title/Abstract] OR ("Under"[All Fields] AND "attack"[Title/Abstract]) OR "Displaced"[Title/Abstract] OR "temporary accommodation"[Title/Abstract] OR "temporary settlement"[Title/Abstract] OR "temporary housing"[Title/Abstract] OR (("transit"[All Fields] OR "transited"[All Fields] OR "transiting"[All Fields] OR "transition"[All Fields] OR "Transitional"[All Fields] OR "transitionals"[All Fields] OR "transitioned"[All Fields] OR "transitioning"[All Fields] OR "transitions"[All Fields] OR "transits"[All	35,906	2:24:50

For peer review only

				Fields]) AND "Settlement"[Title/Abstract]) OR "transitional shelter"[Title/Abstract] OR "emergency shelter"[Title/Abstract] OR "emergency accommodation"[Title/Abstract] OR "emergency housing"[Title/Abstract] OR ("Makeshift"[All Fields] AND "shelter"[Title/Abstract]) OR ("Makeshift"[All Fields] AND "Accommodation"[Title/Abstract]) OR ("Slash"[Title/Abstract] AND "burn cultivation"[Title/Abstract]) OR "shifting cultivation"[Title/Abstract] OR "Feral"[Title/Abstract])		

For peer review only

8	((((((((((((((((((((((Vulnerable[Title/Abstract]) OR (Marginal[Title/Abstract])) OR (Marginalised[Title/Abstract])) OR (Marginalized[Title/Abstract])) OR (Refugee[Title/Abstract])) OR (Homeless[Title/Abstract])) OR (Migrant[Title/Abstract])) OR (financial catastrophe[Title/Abstract])) OR (poverty[Title/Abstract])) OR (conflict-affected[Title/Abstract])) OR (Under attack[Title/Abstract])) OR (Displaced[Title/Abstract])) OR (Temporary Accommodation[Title/Abstract])) OR (Temporary Settlement[Title/Abstract])) OR (Temporary Housing[Title/Abstract])) OR (Transitional Settlement[Title/Abstract])) OR (Transitional shelter[Title/Abstract])) OR (Emergency shelter[Title/Abstract])) OR (Emergency accommodation[Title/Abstract])) OR (Emergency housing[Title/Abstract])) OR (Makeshift shelter[Title/Abstract])) OR (Makeshift accommodation[Title/Abstract])) OR (Slash[Title/Abstract] AND Burn Cultivation[Title/Abstract])) OR (Shifting Cultivation[Title/Abstract])) OR (Feral[Title/Abstract])			"Vulnerable"[Title/Abstract] OR "Marginal"[Title/Abstract] OR "Marginalised"[Title/Abstract] OR "Marginalized"[Title/Abstract] OR "Refugee"[Title/Abstract] OR "Homeless"[Title/Abstract] OR "Migrant"[Title/Abstract] OR "financial catastrophe"[Title/Abstract] OR "poverty"[Title/Abstract] OR "conflict-affected"[Title/Abstract] OR ("Under"[All Fields] OR "attack"[Title/Abstract]) OR "Displaced"[Title/Abstract] OR "temporary accommodation"[Title/Abstract] OR "temporary settlement"[Title/Abstract] OR "temporary housing"[Title/Abstract] OR (("transit"[All Fields] OR "transited"[All Fields] OR "transiting"[All Fields] OR "transition"[All Fields] OR "Transitional"[All Fields] OR "transitionals"[All Fields] OR "transitioned"[All Fields] OR "transitioning"[All Fields] OR "transitions"[All Fields] OR "transits"[All Fields]) OR "Settlement"[Title/Abstract]) OR "transitional shelter"[Title/Abstract] OR "emergency shelter"[Title/Abstract] OR "emergency accommodation"[Title/Abstract] OR "emergency housing"[Title/Abstract] OR ("Makeshift"[All Fields] OR "shelter"[Title/Abstract]) OR ("Makeshift"[All Fields] OR "Accommodation"[Title/Abstract]) OR ("Slash"[Title/Abstract] OR "burn cultivation"[Title/Abstract]) OR "shifting cultivation"[Title/Abstract] OR "Feral"[Title/Abstract]	2,716,400	2:24:36

For peer review only

7	(((Child*[Title/Abstract]) OR (((Under five[Title/Abstract]) OR (Under 5[Title/Abstract])) OR (U5[Title/Abstract]))) OR (((Newborn[Title/Abstract]) OR (Infant[Title/Abstract])) OR (1000 days[Title/Abstract]))) AND (((Intervention*[Title/Abstract]) OR (Strateg*[Title/Abstract])) OR (Practice*[Title/Abstract]))			("child*"[Title/Abstract] OR ("under five"[Title/Abstract] OR "under 5"[Title/Abstract] OR "U5"[Title/Abstract]) OR ("Newborn"[Title/Abstract] OR "Infant"[Title/Abstract] OR "1000 days"[Title/Abstract])) AND ("intervention*"[Title/Abstract] OR "strateg*"[Title/Abstract] OR "practice*"[Title/Abstract])	262,165	2:12:15
6	((Intervention*[Title/Abstract]) OR (Strateg*[Title/Abstract])) OR (Practice*[Title/Abstract])			"intervention*"[Title/Abstract] OR "strategy*"[Title/Abstract] OR "practice*"[Title/Abstract]	2,553,193	2:11:54
5	((Child*[Title/Abstract]) OR (((Under five[Title/Abstract]) OR (Under 5[Title/Abstract])) OR (U5[Title/Abstract]))) OR (((Newborn[Title/Abstract]) OR (Infant[Title/Abstract])) OR (1000 days[Title/Abstract]))			"child*"[Title/Abstract] OR "under five"[Title/Abstract] OR "under 5"[Title/Abstract] OR "U5"[Title/Abstract] OR "Newborn"[Title/Abstract] OR "Infant"[Title/Abstract] OR "1000 days"[Title/Abstract]	1,806,063	2:10:53
4	((Newborn[Title/Abstract]) OR (Infant[Title/Abstract])) OR (1000 days[Title/Abstract])			"Newborn"[Title/Abstract] OR "Infant"[Title/Abstract] OR "1000 days"[Title/Abstract]	350,613	2:10:40
3	(Child*[Title/Abstract]) OR (((Under five[Title/Abstract]) OR (Under 5[Title/Abstract])) OR (U5[Title/Abstract]))			"child*"[Title/Abstract] OR "under five"[Title/Abstract] OR "under 5"[Title/Abstract] OR "U5"[Title/Abstract]	1,559,369	2:09:49
2	((Under five[Title/Abstract]) OR (Under 5[Title/Abstract])) OR (U5[Title/Abstract])			"under five"[Title/Abstract] OR "under 5"[Title/Abstract] OR "U5"[Title/Abstract]	16,327	2:09:27
1	Child*[Title/Abstract]			"child*"[Title/Abstract]	1,554,019	2:09:17

Data Extraction Form

(i) Citation Details

(ii) Research Design

(iii) Target Population

a. Sample Size

b. Inclusion

For peer review only

c. Number of participants

d. Number of sites

e. Type of Setting

f. Exclusion criteria

g. Demographics

i. Age

ii. Gender

(iv) Target Geography

(v) Intervention

a. Duration

b. Number of groups

c. Channels of Delivery

d. Agency for Delivery

e. Service Points

f. End-Users

g. Scale

h. Other Details

(vi) Evaluation

a. Type of Evaluation

b. Outcome Measures

i. Primary

1. ∆Coverage

2. ∆Access

3. ∆Utilization

ii. Secondary

c. Impact Measures

i. Health

1. ∆Morbidity

2. ∆Mortality

3. ∆DALY

4. ∆QALI

ii. Economic

1. RoI

For peer review only

2. ∆Efficiency
3. ∆Cost

REFERENCES

CUSICK, S. E. & GEORGIEFF, M. K. 2016. The Role of Nutrition in Brain Development: The Golden Opportunity of the "First 1000 Days". *J Pediatr,* 175**,** 16-21.
LEVAC, D., COLQUHOUN, H. & O'BRIEN, K. K. 2010. Scoping studies: advancing the methodology. *Implementation science,* 5**,** 1-9.
MILLER, P. M. 2009. An Examination of the McKinney-Vento Act and Its Influence on the Homeless Education Situation. *Educational Policy,* 25**,** 424-450.
ROSENTHAL DM, L. C., HEYS M, SCHOENTHALER AM, UCCI M, HAYWARD A, ET AL. 2021. Barriers to Optimal Health for Under 5s Experiencing Homelessness and Living In Temporary Accommodation in High-Income Countries: A Scoping Review. *Ann Public Health Res,* 8(1):**,** 1103.
ROSENTHAL, D. M., UCCI, M., HEYS, M., HAYWARD, A. & LAKHANPAUL, M. 2020. Impacts of COVID-19 on vulnerable children in temporary accommodation in the UK. *The Lancet Public Health,* 5**,** e241-e242.

For peer review only

PRISMA 2020 Checklist

Section and Topic	Item #	Checklist item	Location where item is reported
**TITLE**			
Title	1	Identify the report as a systematic review.	Title
**ABSTRACT**			
Abstract	2	See the PRISMA 2020 for Abstracts checklist.	Abstract
**INTRODUCTION**			
Rationale	3	Describe the rationale for the review in the context of existing knowledge.	Introduction
Objectives	4	Provide an explicit statement of the objective(s) or question(s) the review addresses.	Introduction
**METHODS**			
Eligibility criteria	5	Specify the inclusion and exclusion criteria for the review and how studies were grouped for the syntheses.	Eligibility Criteria
Information sources	6	Specify all databases, registers, websites, organisations, reference lists and other sources searched or consulted to identify studies. Specify the date when each source was last searched or consulted.	Information sources and search strategy
Search strategy	7	Present the full search strategies for all databases, registers and websites, including any filters and limits used.	Information sources and search strategy
Selection process	8	Specify the methods used to decide whether a study met the inclusion criteria of the review, including how many reviewers screened each record and each report retrieved, whether they worked independently, and if applicable, details of automation tools used in the process.	Study Selection
Data collection process	9	Specify the methods used to collect data from reports, including how many reviewers collected data from each report, whether they worked independently, any processes for obtaining or confirming data from study investigators, and if applicable, details of automation tools used in the process.	Data charting process
Data items	10a	List and define all outcomes for which data were sought. Specify whether all results that were compatible with each outcome domain in each study were sought (e.g. for all measures, time points, analyses), and if not, the methods used to decide which results to collect.	Data charting process
	10b	List and define all other variables for which data were sought (e.g. participant and intervention characteristics, funding sources). Describe any assumptions made about any missing or unclear information.	Data charting process
Study risk of bias assessment	11	Specify the methods used to assess risk of bias in the included studies, including details of the tool(s) used, how many reviewers assessed each study and whether they worked independently, and if applicable, details of automation tools used in the process.	Methodological Quality Appraisal
Effect measures	12	Specify for each outcome the effect measure(s) (e.g. risk ratio, mean difference) used in the synthesis or presentation of results.	NA
Synthesis methods	13a	Describe the processes used to decide which studies were eligible for each synthesis (e.g. tabulating the study intervention characteristics and comparing against the planned groups for each synthesis (item #5)).	NA
	13b	Describe any methods required to prepare the data for presentation or synthesis, such as handling of missing summary statistics, or data conversions.	NA
	13c	Describe any methods used to tabulate or visually display results of individual studies and syntheses.	NA
	13d	Describe any methods used to synthesize results and provide a rationale for the choice(s). If meta-analysis was performed, describe the model(s), method(s) to identify the presence and extent of statistical heterogeneity, and software package(s) used.	NA

For peer review only - http://bmjopen.bmj.com/site/about/guidelines.xhtml

PRISMA 2020 Checklist

Section and Topic	Item #	Checklist item	Location where item is reported
	13e	Describe any methods used to explore possible causes of heterogeneity among study results (e.g. subgroup analysis, meta-regression).	NA
	13f	Describe any sensitivity analyses conducted to assess robustness of the synthesized results.	NA
Reporting bias assessment	14	Describe any methods used to assess risk of bias due to missing results in a synthesis (arising from reporting biases).	Methodological Quality Appraisal
Certainty assessment	15	Describe any methods used to assess certainty (or confidence) in the body of evidence for an outcome.	Methodological Quality Appraisal
**RESULTS**			
Study selection	16a	Describe the results of the search and selection process, from the number of records identified in the search to the number of studies included in the review, ideally using a flow diagram.	The Literature Search
	16b	Cite studies that might appear to meet the inclusion criteria, but which were excluded, and explain why they were excluded.	The Literature Search
Study characteristics	17	Cite each included study and present its characteristics.	Characteristics of Intervention Programmes
Risk of bias in studies	18	Present assessments of risk of bias for each included study.	Quality Review
Results of individual studies	19	For all outcomes, present, for each study: (a) summary statistics for each group (where appropriate) and (b) an effect estimate and its precision (e.g. confidence/credible interval), ideally using structured tables or plots.	NA
Results of syntheses	20a	For each synthesis, briefly summarise the characteristics and risk of bias among contributing studies.	Data Summary and Synthesis
	20b	Present results of all statistical syntheses conducted. If meta-analysis was done, present for each the summary estimate and its precision (e.g. confidence/credible interval) and measures of statistical heterogeneity. If comparing groups, describe the direction of the effect.	NA
	20c	Present results of all investigations of possible causes of heterogeneity among study results.	NA
	20d	Present results of all sensitivity analyses conducted to assess the robustness of the synthesized results.	NA
Reporting biases	21	Present assessments of risk of bias due to missing results (arising from reporting biases) for each synthesis assessed.	Quality Review
Certainty of evidence	22	Present assessments of certainty (or confidence) in the body of evidence for each outcome assessed.	Quality Review
**DISCUSSION**			
Discussion	23a	Provide a general interpretation of the results in the context of other evidence.	Discussions
	23b	Discuss any limitations of the evidence included in the review.	Strengths and Limitations
	23c	Discuss any limitations of the review processes used.	Strengths and Limitations
	23d	Discuss implications of the results for practice, policy, and future research.	Conclusion
**OTHER INFORMATION**			

For peer review only

PRISMA 2020 Checklist

Section and Topic	Item #	Checklist item	Location where item is reported
Registration and protocol	24a	Provide registration information for the review, including register name and registration number, or state that the review was not registered.	NA
	24b	Indicate where the review protocol can be accessed, or state that a protocol was not prepared.	NA
	24c	Describe and explain any amendments to information provided at registration or in the protocol.	NA
Support	25	Describe sources of financial or non-financial support for the review, and the role of the funders or sponsors in the review.	Funding
Competing interests	26	Declare any competing interests of review authors.	Competing Interests
Availability of data, code and other materials	27	Report which of the following are publicly available and where they can be found: template data collection forms; data extracted from included studies; data used for all analyses; analytic code; any other materials used in the review.	Data Availability Statement

From: Page MJ, McKenzie JE, Bossuyt PM, Boutron I, Hoffmann TC, Mulrow CD, et al. The PRISMA 2020 statement: an updated guideline for reporting systematic reviews. BMJ 2021;372:n71. doi: 10.1136/bmj.n71

For more information, visit: http://www.prisma-statement.org/

For peer review only

BMJ Open

Interventions to promote the health and well- being of children under 5s experiencing homelessness in high-income countries: A Scoping Review

Journal:	*BMJ Open*
Manuscript ID	bmjopen-2023-076492.R1
Article Type:	Original research
Date Submitted by the Author:	29-Nov-2023

Complete List of Authors:	Tu, Yanxin; University College London Great Ormond Street Institute of Child Health, Population, Policy and Practice Research and Teaching Department<br>Sarkar, Kaushik; Aceso Global Health Consultants Limited<br>Svirydzenka, Nadia; De Montfort University Faculty of Health and Life Sciences, School of Applied Social Sciences<br>Palfreyman, Zoe; De Montfort University Faculty of Health and Life Sciences, School of Applied Social Sciences<br>Parry, Yvonne; Flinders University, College of Nursing and Health Sciences<br>Ankers, Matthew; Flinders University, College of Nursing and Health Sciences<br>Parikh, Priti; University College London The Bartlett School of Sustainable Construction<br>Raghavan, Raghu; De Montfort University Faculty of Health and Life Sciences, School of Nursing and Midwifery<br>Lakhanpaul, Monica; University College London Great Ormond Street Institute of Child Health, Population, Policy and Practice Research and Teaching Department

**Primary Subject Heading**:	Public health
Secondary Subject Heading:	Paediatrics
Keywords:	Nutrition < TROPICAL MEDICINE, MENTAL HEALTH, Child protection < PAEDIATRICS, Community child health < PAEDIATRICS

SCHOLARONE™
Manuscripts

I, the Submitting Author has the right to grant and does grant on behalf of all authors of the Work (as defined in the below author licence), an exclusive licence and/or a non-exclusive licence for contributions from authors who are: i) UK Crown employees; ii) where BMJ has agreed a CC-BY licence shall apply, and/or iii) in accordance with the terms applicable for US Federal Government officers or employees acting as part of their official duties; on a worldwide, perpetual, irrevocable, royalty-free basis to BMJ Publishing Group Ltd ("BMJ") its licensees and where the relevant Journal is co-owned by BMJ to the co-owners of the Journal, to publish the Work in this journal and any other BMJ products and to exploit all rights, as set out in our licence.

The Submitting Author accepts and understands that any supply made under these terms is made by BMJ to the Submitting Author unless you are acting as an employee on behalf of your employer or a postgraduate student of an affiliated institution which is paying any applicable article publishing charge ("APC") for Open Access articles. Where the Submitting Author wishes to make the Work available on an Open Access basis (and intends to pay the relevant APC), the terms of reuse of such Open Access shall be governed by a Creative Commons licence – details of these licences and which Creative Commons licence will apply to this Work are set out in our licence referred to above.

Other than as permitted in any relevant BMJ Author's Self Archiving Policies, I confirm this Work has not been accepted for publication elsewhere, is not being considered for publication elsewhere and does not duplicate material already published. I confirm all authors consent to publication of this Work and authorise the granting of this licence.

For peer review only

Interventions to promote the health and well- being of children under 5s experiencing homelessness in high-income countries: A Scoping Review

By

Yanxin Tu[1], Kaushik Sarkar[2], Nadia Svirydzenka[3], Zoe Palfreyman[3], Yvonne K. Parry[4], Matthew Ankers[4], Priti Parikh[5], Raghu Raghavan[6], and Monica Lakhanpaul[1*]

*Corresponding author, email: m.lakhanpaul@ucl.ac.uk

Author affiliations

1. Population, Policy and Practice Research and Teaching Department, UCL Great Ormond Street Institute of Child Health, University College London, London, United Kingdom, WC1N 1EH,

2. Aceso Global Health Consultants Limited, Institute for Health Modelling and Climate Solutions, Forecasting Healthy Futures, 4 Bloomsbury Square London, WC1A 2RP

3. School of Applied Social Sciences, Faculty of Health and Life Sciences, De Montfort University, The Gateway, Leicester, United Kingdom, LE1 9BH

4. College of Nursing and Health Sciences, Caring Future Institute, Flinders University, Bedford Park, Sturt South, Adelaide 5001, South Australia, Australia

5. Bartlett School of Sustainable Construction, The Bartlett, UCL Faculty of the Built Environment, 2nd Floor 1-19 Torrington Place London, WC1E 7HB

6. Mary Seacole Research Centre (diversity, culture, and mental health), School of Nursing and Midwifery, Faculty of Health and Life Sciences, De Montfort University, The Gateway, Leicester, United Kingdom, LE1 9BH

For peer review only - http://bmjopen.bmj.com/site/about/guidelines.xhtml

ABSTRACT:

Objectives Homelessness among families with children under 5 residing in temporary accommodation, is a growing global concern, especially in High-Income Countries (HICs). Despite significant impacts on health and development, these "invisible" children often fall through the gaps in policy and services. The study's primary objective is to map the content and delivery methods of culturally sensitive interventions for children under 5 experiencing homelessness in HICs.

Design A scoping review guided by the Preferred Reporting Items for Systematic reviews and Meta-Analyses extension for Scoping Reviews (PRISMA-ScR) checklist.

Data sources Databases include PubMed, MEDLINE, SCOPUS, The Cochrane Library, and Google Scholar were searched up to 24 March 2022.

Eligibility criteria This scoping review includes studies that describe, measure, or evaluate intervention strategies aimed at improving child health programs, specifically those yielding positive outcomes in key areas like feeding, nutrition, care practices, and parenting.

Data extraction and synthesis Articles were selected and evaluated by two independent reviewers, with a dispute resolution system involving a third reviewer for contested selections. The methodological quality of the studies was assessed using various tools including the Risk of Bias (RoB) tool, Cochrane RoB 2.0, RoBANS, and GRADE, each selected according to the type of article.

Results The database search yielded 951 results. After deduplication, abstract screening, and full review, 13 articles met the inclusion criteria. Two predominant categories of intervention delivery methods were identified in this research: group-based interventions (educational sessions) and individual-based interventions (home visits).

Conclusion This review highlights effective interventions for promoting the health and well-being of children under 5 experiencing homelessness, including educational sessions and home visits. Research has supported the importance of home-visiting to be instrumental in breaking down language, cultural, and health literacy barriers.

Keywords: children under 5s; homelessness; parenting support; interventions; feeding; nutrition; mental health; well-being;

For peer review only

STRENGTHS AND LIMITATIONS:

- Adhered to the Arksey and O'Malley framework and the PRISMA-ScR checklist, ensuring a focused and methodical approach.
- The employment of diverse methodological quality assessment tools for different study types allows for a thorough and nuanced evaluation of potential biases, increasing the reliability of this review's conclusions.
- Unlike systematic reviews, this review did not conduct formal data synthesis, potentially limiting the comprehensiveness of evidence overview.
- High heterogeneity, resulting from the inclusion of various study designs, and low generalizability due to the restriction of studies to those conducted in HICs, limits the comparative and broader applicability of the results.

INTRODUCTIONS:

As per the McKinney-Vento definition of homeless, homeless children and youths indicates individuals who lack a fixed, regular, and adequate night-time residence [1]. This definition includes children and youths who are doubling up with others due to housing loss or economic challenges, residing in motels, hotels, trailer parks, or camping grounds due to lack of alternative adequate accommodations, living in emergency or transitional shelters, or abandoned in hospitals. It also covers those living in places not typically used for regular sleeping, such as cars, parks, public spaces, abandoned buildings, substandard housing, bus or train stations, as well as migratory children living in similar conditions[2]. The UN Office of the High Commissioner for Human Rights, acknowledges that homelessness has "emerged as a worldwide human rights concern," especially in high-income nation-states with the means to address it [3]. Homelessness among families with children is now a growing problem in High-Income Countries (HICs). Countries with Gross National Income (GNI) per capita of $13,206 or more are defined as HICs [4]. Between 2014 and 2018, family homelessness almost doubled in Ireland, rising from 407 to over 1,600 families. Between 2006 and 2013, New Zealand had a 44% rise in family homelessness. In 2018, the United States had around 56,300 families with children, accounting for one-third of the country's homeless population [5]. In 2019, the charity Shelter reported that a child loses their home every 8 min in Great Britain, which is the equivalent of 183 children per day. According to a 2019 report from the charity organization Shelter, one child in Great Britain becomes homeless every 8 minutes. This approximates to 183 children daily [5]. According to the Children's Commissioner, there might be as many as 210,000 homeless children living in temporary accommodation, or couch surfing in England, as well as roughly 585,000 people who are either homeless or in danger of becoming homeless.

In particular, the plight of children aged five years or less residing in temporary accommodation is often overlooked or underrecognized. There is a lack of policy supporting them since they are not seen on the streets as homeless. However, many of the children have pre-existing conditions such as epilepsy, asthma, anxiety, and diabetes, and potentially are the most susceptible to viral infection [5]. Moreover, the first five years of life is a critical duration for

For peer review only - http://bmjopen.bmj.com/site/about/guidelines.xhtml

the optimal development of the brain. This is especially important for children who experience poverty/housing/transient lifestyle, as this places them at risk of failing to reach the full development of the brain, potentially leading to many health concerns and issues with language development and motor skills and social problems [6].

Our partnership initially concentrated on evidence-based and community-based participatory engagement approaches that enhance the health and wellbeing of children under 5s who are or at risk of being homeless. This was informed by an investigation into how services could adapt to, and learn from, global public health interventions and family experiences, the purpose of which was to firstly tailor current health strategies for effective outreach to populations who are homeless or at risk of homelessness. Then, secondly, in light of both existing and new findings, provide easily accessible resources to health and social care professionals, as well as families of "invisible" children. These children were excluded from research and national policies, as they are often not counted by services, or are not seen as existing as "homeless." However, current literature did not elucidate on the existing intervention strategies, the delivery channels used by these strategies, the language and cultural barriers encountered, the methods for circumventing these barriers, and the creation of acceptable materials for children under 5s. To guide the literature search and review, the authors predefined several themes of importance for the health and wellbeing of homeless children: feeding and nutrition, care practices, dental care, mental health and wellbeing, safe sleeping, physical activity, and parenting support. These themes, identified as common problems in homeless populations and known to have been effectively addressed in other Low- and Middle-Income Countries (LMICs) [7, 8], served as the foundation for this scoping review. These themes are critical for the integrated health of children under 5s living in temporary accommodation, and there is a complex need to incorporate all these aspects to address or alleviate the current situation of homelessness.

The primary objective of this review is to map the content, and method of delivery of interventions that are culturally sensitive and accessible for populations at the crossroads of poverty/housing/transient lifestyle and focussed on children under 5s. Specifically, our study aims to address the following points:

- Develop inclusive and engaging practice interventions for populations living in temporary accommodation/homelessness, with children under 5s
- Identify the potential critical points of contact. These refer to individuals who play pivotal roles in delivering health services or interventions and engaging with the target population. They serve as key intermediaries, bridging the gap between the health system or intervention and the individuals it aims to benefit.
- Understand how to communicate with mobile populations who have poor health knowledge, literacy, and/or language barriers
- Identify methods of creating appropriate, acceptable, and accessible communication materials

For peer review only - http://bmjopen.bmj.com/site/about/guidelines.xhtml

METHODS:

This focused and methodical scoping review, which was enriched by insights and real-world experiences from global experts, followed the guidelines set forth by the Arksey and O'Malley framework [9] as well as the PRISMA-ScR checklist for scoping reviews [10]. This review follows a predefined protocol (see Supplemental Material 1). There was no patient and public involvement in this study. In aligning with the objectives and methodology of a scoping review, this study embraces an exploratory approach, encompassing a wide range of topics related to the health of homeless children.

1. Eligibility Criteria

The eligibility criteria for our scoping review are structured to include studies that are in English, published between 2000 to 2022, and conducted in High-Income Countries (HICs) as defined by the World Bank [4]. The study focused on interventions targeted at children aged 5 years or less from marginalized or socially excluded families/population groups. It included studies that described, measured, or evaluated a pilot or implementation of a strategy, tactic, process, and/or method targeted at improving child health programmes, with a specific focus on mapping the culturally sensitive approaches. The interventions included were those that demonstrated improvements in outcomes such as service coverage and optimization, access, utilization, and specifically in the nine identified areas of 1. Feeding, 2. Nutrition, 3. Care practice, 4. Parenting, 5. Dental, 6. Wellbeing, 7. Mental health, 8. Safe sleeping, and 9. Physical activity. Studies in languages other than English, from non-HICs as per the World Bank, targeting children over 5 years, involving non-marginalized groups, or those not demonstrating improvement in the outlined 9 areas were excluded. By adhering to these eligibility criteria, the scoping review will ensure that the findings are based on the highest quality evidence and are relevant to the topic of interest. In case the number of these studies is less than 30, we will broaden our investigation to include studies on interventions aimed at children over the age of 5, but only if they offer valuable insights or strategies that can be adapted for younger age groups.

2. Information Sources and Search Strategy

The databases searched from inception of this project up to 24 March 2022 included: PubMed, MEDLINE, SCOPUS, The Cochrane Library (Cochrane Database of Systematic Reviews, Cochrane Central Register of Controlled Trials [CENTRAL]), and Google Scholar. One researcher (YT) developed the search strategy after preliminary deliberations and consensus within the review team. Conference abstracts and Third Sector Reports were also searched for grey literature. Experts from Donor/ Philanthropic organizations and other key experts were contacted for any additional published or unpublished work.

The search strategy was developed to ensure that any relevant studies were identified helping to make the review exhaustive. In the search for literature on the homeless population, two concepts were utilized: 'marginalization' and 'social exclusion'. The search strategy was

For peer review only - http://bmjopen.bmj.com/site/about/guidelines.xhtml

comprised of key search terms drawn from current search strings and customized for each electronic database. We also conducted the relational and citation search to screen cited articles of the 1st iteration of selections, followed by citations and related articles for each inclusion in PubMed, Google Scholar, and PubMed respectively.

3. Study Selection

Search results were uploaded to the Covidence software management system [11] where several reviewers screened content. All articles were extracted and compiled in a single spreadsheet. The spreadsheet was equipped with duplicate study filters, and inclusion filters (inclusion criteria not managed through Search Term) and selection columns. Inclusion and selection filters were filled in independently by one reviewer (YT). All studies selected by two reviewers in Covidence were included, while all studies rejected by two reviewers in Covidence, were rejected. All studies marked for inclusion by one reviewer were marked as disputed selection. In the case of disputes, a third expert reviewer (ML) made the final inclusion decision. Two round of screening was undertaken: Title-Abstract (TiAb) and Full Text (FT). The reasons for exclusion was recorded and mapped in a flow chart, as per PRISMA guidelines.

4. Data charting process

A data extraction form (DEF) was developed as per CEB Critically Appraised Topics (CAT) guidelines by KS and piloted by YT on the first 6 article included. The piloting essentially informed whether the DEF could extract necessary and sufficient information as per the objectives set. The form was then amended based on the pilot and the finalized form used to extract data from full-text articles. The authors abstracted data on article characteristics (target geography, study design), demographic characteristics (age and gender), and intervention characteristics (number of participants, number of sites, type of setting, duration, channels and agency of delivery, service point, end-users).

5. Methodological Quality Appraisal

The methodological quality of included articles was assessed using different tools, relative to the article type. The assessment of Risk of Bias (RoB) tool was only used for interventional studies when results were quantitative analysis or pooling. GRADE [12] was used for assessment of bias in selective publications and selective non-reporting for systematic reviews. For randomized trials, Cochrane RoB 2.0 [13] was used to assess multiple sources of bias and RoBANS [14] was used for assess bias due to selective non-reporting and bias in the selection of the reported result. All studies were critically appraised as per the CAT grading.

For peer review only - http://bmjopen.bmj.com/site/about/guidelines.xhtml

6. Data Synthesis

The data from the included studies were collated, and a summary table was constructed to present the characteristics and conclusions of each study (Table 1). This table encapsulated key information including study authors, age group targeted, geographical location, sample size, contact points, intervention outcomes, and their primary focus. The integration of our findings focused on extracting meaningful insights from the commonalities and variances across different studies. This included identifying effective communication strategies and contact points that are crucial in reaching and engaging families and children in need. We synthesized the information to identify common themes, participants characteristics, delivery methods, critical point of contact, and communication strategy with poor health literacy population.

7. Patient and Public Involvement

No patient involved

RESULTS:

1. The Literature Search

The database search yielded a total of 951 results; no additional articles were found in the grey literature search. 523 records were evaluated after deduplication. After screening title and abstract, 433 relevant articles were excluded. This resulted in 90 articles for review, of which 77 were excluded, as they did not meet the inclusion and exclusion criteria (Figure 1).

For peer review only

2. Data Summary and Synthesis

Data from included studies were collated, and Table 1 below presents characteristics and conclusions reported.

Table 1. Summarization in data extraction of included studies.

Authors	Age	Location	Sample size	Contact Points	Intervention focus
Burgi et al., 2012[15]	5.2 years (SD=0.6)	Switzerland	*N* =652	Health promoters (volunteers) and preschool teacher	Sessions of Physical activity (PA) for children, workshops for teachers, interactive information and discussion evenings for parents
Foka et al., 2021[16]	7-12 years	Greece	*N* =72	Trained facilitators (volunteers)	Resilience-building program with group-focused interactive educational activities
Goodman et al., 2022[17]	6 months	USA	*N* =1243	Trained nurses and staff	A home visit of two hours duration is implemented with the aim to enhance the health and overall well-being of families having infants and young children
Grace et al., 2019[18]	15 months	Australia	*N* =363	Trained volunteers	Weekly 2-hour home visits by trained volunteers, targeting social isolation, community connectedness, and parenting skills
Gross et al., 2009[19]	2-4 years	USA	*N* =292	Trained volunteers	11 weekly video scenarios and group discussions for parental behavior enhancement
Guerrero et al., 2021[20]	0-12 years old	USA	*N* =1749	Trained staff (volunteers)	Group-based educational sessions on stress, depression, and parenting

For peer review only - http://bmjopen.bmj.com/site/about/guidelines.xhtml

Holtrop and Holcomb, 2018[21]	3.18 years old (SD=1.66)	USA	*N* =12	Marriage and family therapist	Parent management training encourages parenting strategies through educational sessions, practice tasks, and satisfaction surveys
Melley, 2010[22]	2 weeks - 3 years old	USA	*N* =87	Trained nurses and parents	Enhanced children's resilience in homelessness through interactive therapy sessions
Ristkari et al., 2019[23]	4 years old	Finland	*N* =463	Professionals in health care and social services	Web-based parent training for basic and practical positive parenting skills, with follow-up telephone coaching to improve behavior
Rowe et al., 2012[24]	0 to 4 years old	Australia	*N* =116	Early childhood professionals and trained nurses	Tweddle Child & Family Health Service (TCFHS) and Day Stay Program (DSP) offer individual, and group level educational sessions for positive parenting skills, focusing on sleep, feeding, and establishing routines
Dugravier et al., 2013[25]	prenatal to 24 months old	France	*N* =440	Phycologists	Psychologists' home visits for mental wellness, attachment, and depression management over 14 sessions
Spijkers et al., 2010[26]	9-11 years	Netherland	*N* =160	Trained nurses	Multilevel parenting program promoting children's social, emotional, and behavioral development through consultations, educational sessions, home visits, and parent-child interactions.
Yousey et al., 2007[27]	18 months-6 years old	USA	*N* =56	Trained nurses	Improving homeless children's nutrition by educating parents and shelter food providers through sessions developed by dietitians

For peer review only - http://bmjopen.bmj.com/site/about/guidelines.xhtml

3. Quality Review

Based on the screening results of Cochrane RoB 2.0, the study by Grace et al. exhibited a risk of performance bias. Caregivers and personnel administering the interventions were aware of participants' allocated intervention throughout the trial, as this information was disclosed by participants in their questionnaire responses about their experiences with services [18]. Furthermore, there was a risk of selection bias in the study by Gross et al. due to issues with the randomization process. Parents were not randomly assigned to different intervention levels, but instead chose how many group sessions they attended. Consequently, observed improvements in child behaviour might be attributed to factors associated with parental attendance, rather than the intervention itself [19]. Study of Goodman et al. is limited by the use of self-report data, which could contribute to recall and social-desirability bias [17]. Concerns were also raised regarding bias due to missing data for study by Dugravie et al. Only half of the participants completed their perinatal home-visiting program [25].

In light of the screening results of RoBANS, study of Guerrero et al. is also constrained by the use of self-report data [20]. The challenge of retaining parents was also a notable drawback of the research by Holtrop et al., since only 50 of the participants attended more than fifty percent of the sessions [21]. In general, we did not exclude any studies based on risk of bias assessment.

4. Characteristics of Intervention Programmes

4.1 Intervention Themes

In total, 13 studies were included for review (Table 1). Of the nine targeted intervention themes in the inclusion criteria, none pertaining to dental outcomes for marginalized children in HICs were identified in the search. Several overlapping themes, such as feeding, nutrition, safe sleeping, and mental health, were commonly addressed through enhancing positive parenting skills and support. Overall, four intervention theme categories were identified from the evidence synthesis.

- Positive Parenting Skills (sleeping and settling, safe sleep, feeding and meal time, establishing routines, attachment and boding) [17, 19, 21-24]
- Mental Health and Wellbeing (social isolation, chronic stress and depression, toddler behavior) [16, 18, 20, 25, 28]
- Nutrition (Improve nutrition knowledge of parents) [27]
- Physical Activity (life style, adiposity and fitness) [15]

4.2 Characteristics of Participants

Upon an initial exploration of the literature, the number of studies within this age range was limited. Given the importance of our research question and the potential benefits of gaining a

For peer review only - http://bmjopen.bmj.com/site/about/guidelines.xhtml

broader understanding of the available interventions, this study encompassed studies involving children above five. The majority of the studies encompassing children aged under 5-year-olds [15, 17-25, 27], 2 studies included were targeted on early childhood (ages 6 -11) [16, 28]. The justification for this approach lies in the potential transferability of interventions for slightly older children to younger age groups.

Gender distribution is generally even and homogeneous across different study. However, in a non-randomized intervention study by Holtrop et al. [21], the sample consisted of 75% females and 25% males. Gender distribution was not specified in the studies by Yousey et al. and Guerrero et al. [20, 27]. The backgrounds of the participants varied widely across the included studies. Participants encompassed children of migrant and/or parents with low education levels, children from homeless or poverty-stricken families, new-born infants from transitional housing communities, and families living in low socioeconomic areas.

4.3 The Method of Delivery

Detailed description of the intervention programmes are shown in Table 1. The intervention duration varies from 1 week to 2 years, with four included studies having intervention durations of over 12 months [15, 18, 19, 25], four studies ranging from 6 to 12 months [17, 20, 21, 28], and five studies lasting less than 6 months [16, 22-24, 27]. The interventions identified in this study have been broadly categorized as follows:

- Education-based group interventions, performed as group-based intervention, such as those based on the Webster-Stratton Model [29] and the Oregon Model [30], are effective in improving parenting practices and child behaviour, especially in socio-economically disadvantaged communities [15, 19, 21, 23, 24, 27]. These include methods such as web coaching, videotaped vignettes, group discussions, and family meetings. Broadening the intervention scope to include other involved adults and offering education on nutrition, physical activity, media use, and sleep further boosts their efficacy. Programs like the Strengths for the Journey in refugee camps have utilized positive psychology concepts[16], while the Parent-Child Interaction Therapy model [31] successfully strengthened parent-child relationships in shelters. Overall, these education-based group interventions offer a cost-effective, holistic approach to child welfare.

- Home-visiting interventions involved trained professionals offering support and education to families with young children in their homes, with interventions being adapted to each family's unique needs, often complemented by group-based interventions. The goal was to foster community connectivity and independent utilization of services [14, 16, 17]. Some studies, such as Guerrero et al.'s work, supplement these home visits with group-based education, training staff and parents in stress and depression management via a flexible, Train-the-Trainer approach [18]. Additionally, the Positive Parenting Program (Triple P) employs a multilevel system of family intervention combining home-visiting and education session approaches, escalating in intensity based on the severity of the child's behavioural and emotional issues [28].

For peer review only - http://bmjopen.bmj.com/site/about/guidelines.xhtml

4.4 Critical Points of Contact

Marginalized children may face a variety of barriers that can prevent them from accessing healthcare, education, social services, and other resources that are necessary for their well-being. Designing contextual, culturally sensitive, and diverse interventions remains a challenge. However, identifying effective points of contact for interventions can help overcome these obstacles. Studies have utilized maternal and child health nurses [17, 22, 24, 27, 28], and health professionals with relevant expertise [21, 23-25], as critical points of contact. Trained volunteers, often acting as health promoters or facilitators, have also served this role [15, 16, 18-20]. Nurses and trained volunteers are cost-effective, easy to train and access. Health professionals typically possess greater knowledge, skills, and experience, adhering to strict professional standards. It was hypothesized that professionals who were more highly trained in interested field would be more competent in implementing interventions precisely and effectively [10]. The choice of critical points of contact hinges on the study's complexity and scope, warranting a cost-effectiveness analysis.

5. Communication with Poor Health Literacy Population

This review notably aimed to explore strategies for creating suitable, acceptable, and accessible communication materials, especially for mobile populations with limited health knowledge, literacy, or language skills, particularly those with children under five. In relation to the development of such materials, the Chicago Parent Program (CPP), as described in the study by Gross et al., devised an intervention content and strategy. Communication materials were created in partnership with an advisory council of parents, including seven African-American and five Latino parents from different neighbourhoods in Chicago. This council provided input to the program creators about their parenting challenges, the scenarios they'd prefer to see in video format, and the best ways to present parenting techniques that align with their values, lifestyle, and cultural norms. This approach made the content patient-oriented, readily acceptable, and culturally sensitive [19]. Yousey et al. and Guerrero et al. discussed making teaching materials, handouts, and class activities in a low-literacy format, visually appealing (with pictures and varied colours), and game-oriented (puzzles, riddles) as useful strategy that make acceptable, and accessible communication material [20, 27]. As materials developed for marginalized parents with poor health literacy or low education levels, it is important to provide information in different languages. Burgi et al. also equipped native speakers of the main foreign languages to answer any questions from parents throughout the intervention to break language barriers [15]. In terms of the communication method with marginalized population, the reviewed articles emphasized the importance of home-visiting, as a less formal, relationship-based approach is potentially crucial in overcoming barriers to service engagement, such as language and cultural obstacles, and limited health literacy [17, 18, 25, 28].

For peer review only - http://bmjopen.bmj.com/site/about/guidelines.xhtml

DISCUSSIONS:

1. Group-based interventions

Educational sessions were the most commonly employed strategy in group-based interventions included in this scoping review. Providing educational sessions in group level can be a valuable and effective approach to supporting families with children under 5, who experience homelessness in high-income countries. Families experiencing homelessness may feel isolated and disconnected, and participating in a group-based intervention can provide an opportunity for them to connect with other families in similar situations [18]. Therefore, group-based interventions can provide families with a sense of social support and community. Educational sessions can also be tailored to meet the specific needs of families experiencing homelessness with children under 5, since the content of sessions are dependent on the health needs. In short, group-based educational session interventions can empower families experiencing homelessness by providing them with the tools, knowledge, and support they need to improve their situation.

Web-based sessions are an innovated form of carrier of education and thus modifying behaviours and improve health outcomes in children under 5 experiencing homlessness. Parent training programs that utilize technology can have numerous advantages compared to conventional interventions, including improved consistency, enhanced availability, increased convenience, and a reduction in both time and financial expenditure [32, 33]. The concept of technology-based parent training isn't new; dating back to 1988, Webster-Stratton and his team [29] used video recordings as the main medium to conduct a parent training initiative. The innovation of web-based sessions offers an efficient platform for imparting necessary knowledge, modifying behaviours, and improving health outcomes in the children under 5 living in temporary accommodation. Given the high usage of cell phones among the homeless population in High-Income Countries (HICs), the potential for web-based interventions becomes even more evident. This suggests a promising intersection between technology and accessible parent training programs within the context of homelessness. Evidence indicated that most people experiencing homelessness have cell phones in HICs. In a case report conducted in Los Angeles, USA, 85% of homeless people used a cell phone and used text messaging daily, and 51% accessed the Internet on their cell phone [34]. A number of other studies have postulated a convergence on the similar findings [35-37]. The current cell phone using status support the possibility of Web-based intervention use in the homelessness context. Web-based interventions can potentially eliminate the obstacles related to in-person sessions, allowing individuals to pursue assistance for mental health issues without the concern of stigma [38-40]. The web-based intervention was demonstrated to be low cost, low threshold and of great implications for evidence-based interventions in the future. Accordingly, recent studies in the sphere of parent training have pivoted towards online training programs. These interactive online training platforms for parents can successfully surmount numerous obstacles typically encountered during the execution phase of traditional programs, suggesting a promising intersection between technology and accessible parent training programs within the context of homelessness. [23].

For peer review only - http://bmjopen.bmj.com/site/about/guidelines.xhtml

2. Individualized interventions

Parents, and particularly mothers, are susceptible to social isolation, especially during the initial transition to parenthood when they may experience intense feelings of fatigue or a lack of readiness. In research concerning parents facing additional challenges, such as parenting a child with a disability, recent immigration, experiencing cognitive or mental health difficulties, the prevalence of social isolation was found to be significant [41-43]. To address this issue, one commonly utilized strategy is home-visiting. Typically coordinated by a local organization, this intervention involves assigning a volunteer or professional to provide social support to individuals identified as needing additional help. Through regular visits, these health providers offer a variety of support services and work towards enhancing the individual's engagement with formal services, fostering greater community connection, and promoting independent utilization of services.

Individualized interventions (home-visiting) can be more flexible than group-based interventions, as they can be adapted to meet the changing needs of families over time, provide personalized support tailoring to different family's needs, provide families with access to a range of resources and services, and provide a greater sense of confidentiality and privacy. These individualized approaches are instrumental in dismantling barriers to service engagement, including those tied to language and culture [44]. Previous studies advocate for the possible benefits of home visits by non-professionals, such as facilitating the dissemination of health-related information [45], enhancing social networks for those in isolation, fostering emotional wellbeing and parental proficiency, and endorsing positive health outcomes [46]. Furthermore, previous research has emphasized the value of home-visiting, a less formal, relationship-based approach that complements other more structured services in the service landscape [44]. This idea suggests that combining group-level educational sessions with home visits could reinforce training content by allowing for individual feedback collection and catering to personal needs. Given these potential benefits of individualized interventions, the strategy of home-visiting has gained attention in both research and practice. By bridging the benefits of both individualized interventions and community support, home-visiting appears to be a promising strategy that could be more widely applied in future intervention designs for homeless population.

3. Research Gap

While there's a growing focus on understanding barriers to dental services for the homeless population in High-Income Countries (HICs), it's evident that service provision, particularly for homeless children, is woefully inadequate [47-49]. Studies have shown a lack of dental health care services for the homeless across governmental, private, and third-sector levels [47]. Moreover, current peer-reviewed literature is sparse on strategies improving access to, and uptake of, dental care for this marginalized group [50]. A scoping review of grey literature identified only two services specifically catering to the homeless population in Australia [50]. The effectiveness of these services remains unknown, raising questions about their generalizability across various geographic settings or age groups. The issue is pressing considering the vulnerability of marginalized children to adverse dental problems. An estimated three million children in Europe are believed to receive inadequate dental treatment

For peer review only - http://bmjopen.bmj.com/site/about/guidelines.xhtml

[51], and children from low-income families are twice as likely to have cavities compared to those from higher-income households [52]. Consequently, there is an urgent public health need to implement targeted dental services for young children within the homeless population.

In the context of our scoping review, another noticeable gap is that only one study focused specifically on the theme of physical activity [15]. This paucity of research is a concern given the global public health issue of childhood overweight and obesity [53]. In addition, children of migrant parents and those with low socioeconomic status are considered high risk groups for the development of obesity and of low fitness, which is known as the hunger-obesity paradox [54]. In addressing this issue, it was scoped that Burgi et al. developed a lifestyle intervention in Switzerland aimed at enhancing physical activity level in preschool children, primarily from migrant or lower education level (EL) families [15]. This intervention showed beneficial effects, yet it remains an outlier; overall, interventions in these populations have shown less effectiveness [55-58]. Given the magnitude of the obesity and low fitness issue among homeless populations, the paucity of targeted interventions underlines a pressing need for further research. Future studies should aim to develop effective, evidence-based approaches that integrate health promotion programs within the broader context of social and cultural values. This could help in designing interventions tailored to this vulnerable population, thereby addressing this significant gap in the research.

4. Strengths and Limitations

This study represents the first scoping review of peer-reviewed literature dedicated specifically to intervention approaches for marginalized children living in temporary accommodation under five years old across various themes. We deployed a rigorous strategy to extensively map available evidence, with the aim of identifying knowledge gaps to inform future research. The quality of all included studies was high, underscoring the credibility of our findings. Despite these strengths, several limitations should be acknowledged. Unlike systematic reviews, scoping reviews typically do not conduct formal data synthesis. Consequently, the results may not provide as comprehensive or robust an overview of the evidence as systematic reviews do. Furthermore, this review exhibits a high level of heterogeneity due to the incorporation of studies with diverse designs and methodologies, which can make comparing and synthesizing results challenging. Lastly, this review's generalizability may be limited due to the typically small scale of interventions in the included studies. This focus on HICs could also limit the generalizability of the findings to countries with different socioeconomic contexts and resource availability.

CONCLUSIONS:

This review sheds light on health interventions that effectively reach children under five years old who are homeless or at risk of homelessness. It contributes not only to the literature but also provides actionable resources for health and social care professionals and the families of these often 'invisible' children. While a robust body of research focuses on parenting support, mental health, well-being, nutrition and feeding, and care practices, we found a significant gap

For peer review only - http://bmjopen.bmj.com/site/about/guidelines.xhtml

in addressing dental health and over-weight within marginalized families in HICs. The review of thirteen interventions revealed that two primary methods, group-based educational sessions and individual-based home visits, hold promise. Notably, an integrated approach combining these two methods appears to be feasible. It was also identified that health professionals, nurses, and trained volunteers play crucial roles in delivering these interventions. Despite these insights, several areas necessitate further investigation. For example, a comparison of cost-effectiveness between group-based and individualized interventions remains elusive. Moreover, the review identified a common issue of low community connectedness and service utilization, highlighting the need to explore ways to enhance community resource usage in HICs further.

Figures

Figure 1. Preferred Reporting Items for Systematic Reviews and Meta-Analyses (PRISMA) 2020 Flow Diagram.

Acknowledgements

Not applicable.

Contributors

Each author made substantial contributions to the design and execution of the study, and to the writing and revising of this article. YT played a primary role in conceptualizing the study, data collection and analysis, and drafting and revising the manuscript. KS, ML and NS guided the study protocol and developed the search strategy. KS was actively involved in data collection and analysis. ML and NS were key contributor to the study conception and design, guided data analysis, and revised the manuscript. PP, ZP, YP, MA, and RR were responsible for all major areas of concept development and study planning, were consulted on the data analysis and its interpretation and provided manuscript edits. All authors reviewed and finalised the manuscript.

Funding

This work was supported by the ESRC as part of UK Research & Innovation's rapid response to COVID-19 (ES/V016253/1).

Competing interests

The authors report that there are no competing interests to declare.

Patient consent for publication

Not required.

Ethics approval

Research ethics approval was not required as this study did not involve participants.

For peer review only - http://bmjopen.bmj.com/site/about/guidelines.xhtml

For peer review only

Provenance and peer review

Not commissioned; externally peer reviewed.

Data availability statement

Data are available upon reasonable request to the corresponding author (https://orcid.org/0000-0002-9855-2043).

REFERENCES:

1. Miller, P.M., *An examination of the McKinney-Vento Act and its influence on the homeless education situation.* Educational Policy, 2011. **25**(3): p. 424-450.
2. Homeless, N.C.f.t., *Who is Homeless?*, N.C.f.t. Homeless, Editor. 2005.
3. housing, S.R.o.t.r.t.a. *Report on homelessness as a global human rights crisis*. 2015 [cited 2022 04/26]; Available from: https://www.ohchr.org/en/calls-for-input/report-homelessness-global-human-rights-crisis.
4. Bank, W., *World Bank country and lending groups—World Bank data help desk.* World Bank Country and Lending Groups–World Bank Data Help Desk, 2021.
5. Rosenthal, D.M., et al., *Impacts of COVID-19 on vulnerable children in temporary accommodation in the UK.* The Lancet Public Health, 2020. **5**(5): p. e241-e242.
6. Cusick, S.E. and M.K. Georgieff, *The role of nutrition in brain development: the golden opportunity of the "first 1000 days".* The Journal of pediatrics, 2016. **175**: p. 16-21.
7. Zlotnick, C., T. Tam, and S. Zerger, *Common needs but divergent interventions for U.S. homeless and foster care children: results from a systematic review.* Health Soc Care Community, 2012. **20**(5): p. 449-76.
8. Weber, J.J., *A systematic review of nurse-led interventions with populations experiencing homelessness.* Public Health Nurs, 2019. **36**(1): p. 96-106.
9. Levac, D., H. Colquhoun, and K.K. O'Brien, *Scoping studies: advancing the methodology.* Implementation science, 2010. **5**: p. 1-9.
10. Tricco, A.C., et al., *PRISMA extension for scoping reviews (PRISMA-ScR): checklist and explanation.* Annals of internal medicine, 2018. **169**(7): p. 467-473.
11. Innovation, V.H. *Covidence systematic review software, Melbourne, Australia. Available online: www.covidence.org.*
12. Morgan, R.L., et al., *A risk of bias instrument for non-randomized studies of exposures: a users' guide to its application in the context of GRADE.* Environment international, 2019. **122**: p. 168-184.
13. Sterne, J.A., et al., *RoB 2: a revised tool for assessing risk of bias in randomised trials.* bmj, 2019. **366**.
14. Park, J., et al. *Risk of bias assessment tool for non-randomized studies (RoBANS):*

For peer review only - http://bmjopen.bmj.com/site/about/guidelines.xhtml

development and validation of a new instrument. in *19th Cochrane Colloquium*. 2011.

15. Burgi, F., et al., *Effect of a lifestyle intervention on adiposity and fitness in socially disadvantaged subgroups of preschoolers: a cluster-randomized trial (Ballabeina).* Prev Med, 2012. **54**(5): p. 335-40.
16. Foka, S., et al., *Promoting well-being in refugee children: An exploratory controlled trial of a positive psychology intervention delivered in Greek refugee camps.* Dev Psychopathol, 2021. **33**(1): p. 87-95.
17. Goodman, W.B., et al., *Evaluation of a Family Connects Dissemination to Four High-Poverty Rural Counties.* Matern Child Health J, 2022.
18. Grace, R., et al., *Effectiveness of the Volunteer Family Connect Program in Reducing Isolation of Vulnerable Families and Supporting Their Parenting: Randomized Controlled Trial With Intention-To-Treat Analysis of Primary Outcome Variables.* JMIR Pediatr Parent, 2019. **2**(2): p. e13023.
19. Gross, D., et al., *Efficacy of the Chicago parent program with low-income African American and Latino parents of young children.* Prev Sci, 2009. **10**(1): p. 54-65.
20. Guerrero, A.D., et al., *Evaluation of a Health Education Intervention to Improve Parental Knowledge and Attitudes About Chronic Stress and Depression Among Head Start Families.* Health Promot Pract, 2021: p. 15248399211061132.
21. Holtrop, K. and J.E. Holcomb, *Adapting and Pilot Testing a Parenting Intervention for Homeless Families in Transitional Housing.* Fam Process, 2018. **57**(4): p. 884-900.
22. Melley, A.H.C., Kim; Norris-Shortle, Carole; Kiser, Laurel J.; Levey, Eric B.; Coble, Catherine A.; Leviton, Audrey, *Supporting Positive Parenting for Young Children Experiencing Homelessness: The PACT Therapeutic Nursery.* Zero to Three, 2010. **30**(4): p. 39-45.
23. Ristkari, T., et al., *Web-Based Parent Training Intervention With Telephone Coaching for Disruptive Behavior in 4-Year-Old Children in Real-World Practice: Implementation Study.* J Med Internet Res, 2019. **21**(4): p. e11446.
24. Rowe, H., et al., *Admission to day stay early parenting program is associated with improvements in mental health and infant behaviour: A prospective cohort study.* Int J Ment Health Syst, 2012. **6**(1): p. 11.
25. Dugravier, R., et al., *Impact of a manualized multifocal perinatal home-visiting program using psychologists on postnatal depression: the CAPEDP randomized controlled trial.* PLoS One, 2013. **8**(8): p. e72216.
26. Spijkers, W., et al., *Effectiveness of a parenting programme in a public health setting: a randomised controlled trial of the positive parenting programme (Triple P) level 3 versus care as usual provided by the preventive child healthcare (PCH).* BMC Public Health, 2010. **10**: p. 131.
27. Yousey, Y., et al., *Education in a homeless shelter to improve the nutrition of young children.* Public Health Nurs, 2007. **24**(3): p. 249-55.
28. Spijkers, W., D.E. Jansen, and S.A. Reijneveld, *Effectiveness of Primary Care Triple P on child psychosocial problems in preventive child healthcare: a randomized controlled trial.* BMC

For peer review only

medicine, 2013. **11**(1): p. 1-8.

29. Webster-Stratton, C., *Preventing conduct problems in Head Start children: strengthening parenting competencies.* Journal of consulting and clinical psychology, 1998. **66**(5): p. 715.
30. Forgatch, M.S. and G.R. Patterson, *Parent Management Training—Oregon Model: An intervention for antisocial behavior in children and adolescents.* 2010.
31. Fernandez, M.A. and S.M. Eyberg, *Predicting treatment and follow-up attrition in parent–child interaction therapy.* Journal of abnormal child psychology, 2009. **37**(3): p. 431-441.
32. Breitenstein, S.M., D. Gross, and R. Christophersen, *Digital delivery methods of parenting training interventions: a systematic review.* Worldviews on Evidence‐Based Nursing, 2014. **11**(3): p. 168-176.
33. Taylor, T.K., et al., *Computer‐based intervention with coaching: An example using the Incredible Years program.* Cognitive behaviour therapy, 2008. **37**(4): p. 233-246.
34. Rhoades, H., et al., *No digital divide? Technology use among homeless adults.* Journal of Social Distress and the Homeless, 2017. **26**(1): p. 73-77.
35. Rice, E., A. Lee, and S. Taitt, *Cell phone use among homeless youth: potential for new health interventions and research.* Journal of Urban Health, 2011. **88**(6): p. 1175-1182.
36. Freedman, M.J., et al., *Cell phones for ecological momentary assessment with cocaine-addicted homeless patients in treatment.* Journal of substance abuse treatment, 2006. **30**(2): p. 105-111.
37. Tyler, K.A. and R.M. Schmitz, *Using cell phones for data collection: Benefits, outcomes, and intervention possibilities with homeless youth.* Children and Youth Services Review, 2017. **76**: p. 59-64.
38. Griffiths, K.M., et al., *Effect of web-based depression literacy and cognitive–behavioural therapy interventions on stigmatising attitudes to depression: Randomised controlled trial.* The British Journal of Psychiatry, 2004. **185**(4): p. 342-349.
39. Finkelstein, J. and O. Lapshin, *Reducing depression stigma using a web-based program.* International journal of medical informatics, 2007. **76**(10): p. 726-734.
40. Rodda, S., et al., *Web-based counseling for problem gambling: exploring motivations and recommendations.* Journal of medical Internet research, 2013. **15**(5): p. e2474.
41. Grace, R., et al., *Far from ideal: Everyday experiences of mothers and early childhood professionals negotiating an inclusive early childhood experience in the Australian context.* Topics in Early Childhood Special Education, 2008. **28**(1): p. 18-30.
42. McMichael, C. and L. Manderson, *Somali women and well-being: Social networks and social capital among immigrant women in Australia.* Human organization, 2004. **63**(1): p. 88-99.
43. Llewellyn, G., et al., *Support network of mothers with an intellectual disability: An exploratory study.* Journal of Intellectual and Developmental Disability, 1999. **24**(1): p. 7-26.
44. Byrne, F., et al., *Structured social relationships: a review of volunteer home visiting programs for parents of young children.* Australian health review, 2015. **40**(3): p. 262-269.
45. Johnson, Z., F. Howell, and B. Molloy, *Community mothers' programme: randomised controlled trial of non-professional intervention in parenting.* British Medical Journal, 1993. **306**(6890): p.

For peer review only - http://bmjopen.bmj.com/site/about/guidelines.xhtml

1449-1452.

46. Deković, M., et al., *Tracing changes in families who participated in the home-start parenting program: Parental sense of competence as mechanism of change.* Prevention science, 2010. **11**(3): p. 263-274.
47. Paisi, M., et al., *Barriers and enablers to accessing dental services for people experiencing homelessness: A systematic review.* Community Dent Oral Epidemiol, 2019. **47**(2): p. 103-111.
48. King, T.B. and G. Gibson, *Oral health needs and access to dental care of homeless adults in the United States: a review.* Spec Care Dentist, 2003. **23**(4): p. 143-7.
49. Stormon, N., et al., *Facilitating Access to Dental Care for People Experiencing Homelessness.* JDR Clinical & Translational Research, 2021. **6**(4): p. 420-429.
50. Goode, J., H. Hoang, and L. Crocombe, *Strategies to improve access to and uptake of dental care by people experiencing homelessness in Australia: a grey literature review.* Aust Health Rev, 2020. **44**(2): p. 297-303.
51. Rigby, M.J., *Potentially over 3 million children in EU Europe believed not to be receiving needed medical and dental treatment-and parents' reasons why.* Child Care Health Dev, 2020. **46**(3): p. 390-396.
52. Dye, B.A., X. Li, and E.D. Beltran-Aguilar, *Selected oral health indicators in the United States, 2005-2008.* NCHS Data Brief, 2012(96): p. 1-8.
53. Daniels, S.R., *The consequences of childhood overweight and obesity.* The future of children, 2006. **16**(1): p. 47-67.
54. Koh, K.A., et al., *The hunger–obesity paradox: obesity in the homeless.* Journal of Urban Health, 2012. **89**(6): p. 952-964.
55. Danielzik, S., et al., *First lessons from the Kiel obesity prevention study (KOPS).* International Journal of Obesity, 2005. **29**(2): p. S78-S83.
56. Nemet, D., D. Geva, and A. Eliakim, *Health promotion intervention in low socioeconomic kindergarten children.* The Journal of pediatrics, 2011. **158**(5): p. 796-801. e1.
57. Muckelbauer, R., et al., *Promotion and provision of drinking water in schools for overweight prevention: randomized, controlled cluster trial.* Pediatrics, 2009. **123**(4): p. e661-e667.
58. Müller, M.J., S. Danielzik, and S. Pust, *School-and family-based interventions to prevent overweight in children.* Proceedings of the Nutrition Society, 2005. **64**(2): p. 249-254.

For peer review only - http://bmjopen.bmj.com/site/about/guidelines.xhtml

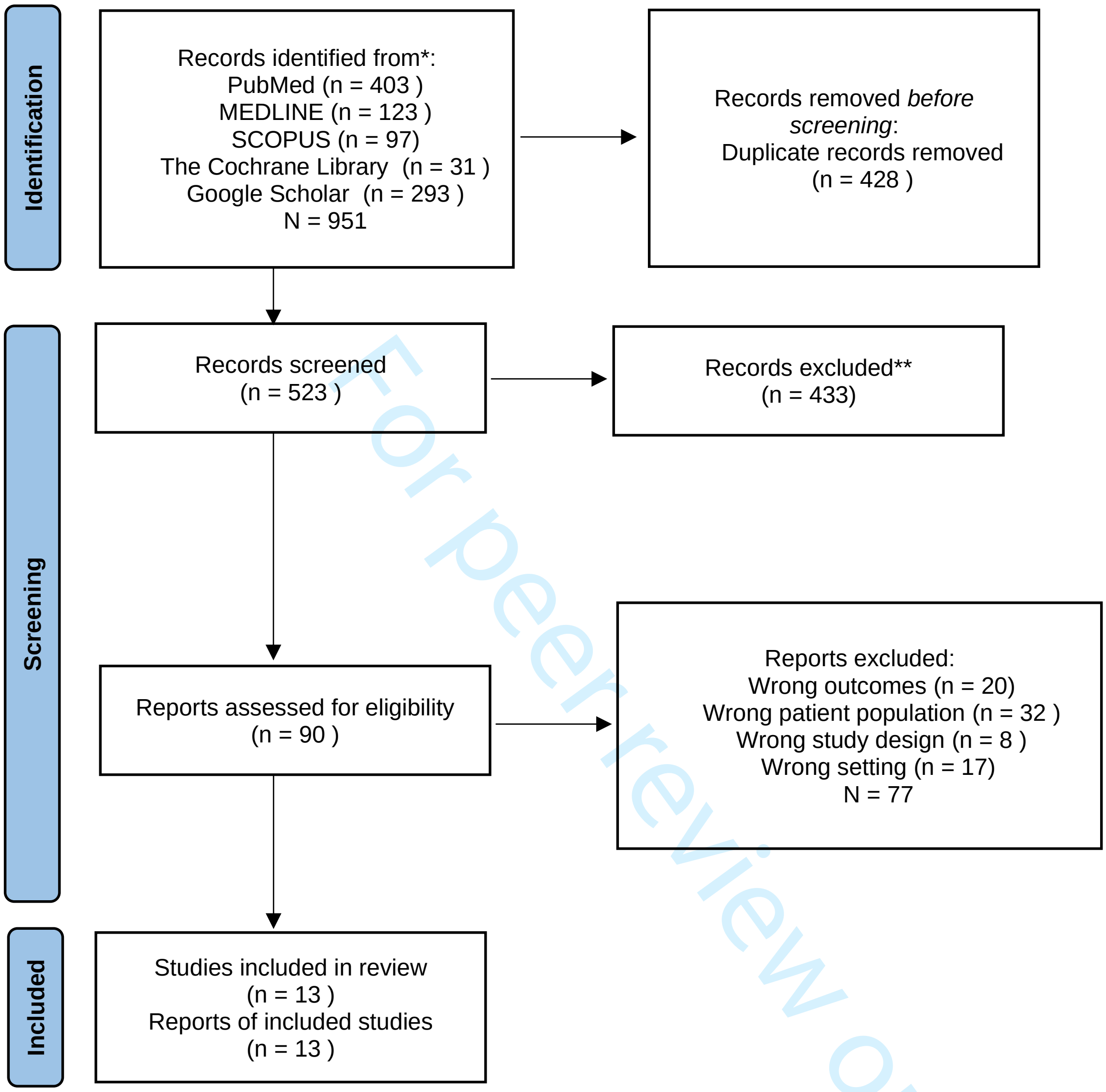

Figure 1. Preferred Reporting Items for Systematic Reviews and Meta-Analyses (PRISMA) 2020 Flow Diagram.

For peer review only

Scoping Review Protocol

TITLE

Interventions to promote the health and well- being of children under 5s experiencing homelessness in high-income countries: A Scoping Review

PROTOCOL INFORMATION

Authors: All team members
Contact person: Monica Lakhanpaul
Dates: Anticipated start date of search: December 2021
Anticipated completion date: March 2022

BACKGROUND

According to the McKinney-Vento definition of homeless, "homeless children and youths" means individuals who lack a fixed, regular, and adequate night-time residence (Miller, 2009). According to the UN Office of the High Commissioner for Human Rights, homelessness has "emerged as a worldwide human rights concern," especially in high-income nation-states with the means to address it. Between 2014 and 2018, family homelessness almost doubled in Ireland, rising from 407 to over 1,600 families. Between 2006 and 2013, New Zealand had a 44% rise in family homelessness. In 2018, the United States had around 56,300 families with children, accounting for one-third of the country's homeless population (Rosenthal DM, 2021). In 2019, the charity Shelter reported that a child loses their home every 8 min in Great Britain, which is the equivalent of 183 children per day. In 2019, the overall number of children who were homeless or in temporary housing in England climbed to 126020, including 88080 in London. According to the Children's Commissioner, there might be around 210000 homeless children in temporary accommodation or couch surfing in England, as well as roughly 585000 people who are either homeless or in danger of becoming homeless.

In particular, young children aged ≤5 years living in temporary accommodation (U5TA) have an inconspicuous plight. There is a lack of policy supporting them since they are not on the streets as homeless. However, many U5TA have pre-existing conditions such as epilepsy, asthma, anxiety, and diabetes; they might be the most susceptible to viral infection (Rosenthal et al., 2020). Moreover, the first five years of life is a critical duration for the optimal development of the brain, especially for children who experience poverty/housing/transient lifestyle, which not only leads to many health concerns but also leads to language development and motor skills and social problems (Cusick and Georgieff, 2016).

Therefore, this scoping review was undertaken to gather existing interventions, especially for families with homeless children within themes: feeding, nutrition, care practice, parenting, dental, wellbeing and mental health, safe sleeping, physical activity, parenting support, which were already effectively conducted in other HICs. Those themes are essential to the integrated health of U5TA, and there is a complex need to include all those themes to address or alleviate the current situation of homelessness. The primary objective of this review is to examine the content and method of delivery of interventions that are culturally sensitive and accessible for the demographic that is on the crossroads of poverty/housing/transient lifestyle. Specifically, our study also aims to address and answer the following question: How best to communicate with mobile populations with poor health literacy/literacy/language barriers? Who are possible and critical points of contact? How to develop an inclusive and engaging practice with

For peer review only - http://bmjopen.bmj.com/site/about/guidelines.xhtml

populations in homelessness? How to create appropriate, acceptable, and accessible communication materials? (e.g., through health visitors, family nurse partnerships, etc.)

The scoping review is a core work package of the project titled, “Children in Homeless Accommodations Managing Pandemic Invisibility or Non-inclusive Strategies” to map the existing global evidence on the content and method of delivery of practical strategies and interventions to improve coverage, access, and utilization of early child health programmes in marginalized and excluded under5s.

OBJECTIVES

- O-1. To identify the evidence base available around the content of delivery of interventions programmes for families with children under 5.
- O-2. To explore the method of delivery of these interventions: when to intervene, how to intervene, entry points for crucial health concerns.
- O-3. To find out the most feasible settings in which the strategies and interventions are effective

Specific parameters that will be mapped from studies that relate to the review objectives will comprise:

(i) Challenge area

(ii) Settings of interventions (Content and delivery method)

(iii) Outcome of the interventions

(iv) Culturally accessible/appropriate for mobile populations

(v) Scale of impact

(vi) Economy of impact

METHODS

We will conduct a targeted systematic rapid global scoping review (supplemented by knowledge and lived experiences from international experts) using the Arksey and O’Malley framework (Levac et al., 2010)

1.1. Review Methods

1.1.1. Inclusion Criteria

1.1.1.1. **Language and Geography:** English language studies of HICs;

1.1.1.2. **Types of studies:** Studies of any design, where the study objective was to describe, measure, or evaluate the piloting or implementation of a strategy, tactics, process, and/or method targeted at improving child health programmes in High-income countries;

1.1.1.3. **Types of participants:** Studies that include interventions for under-5 children; The review will look into two qualifiers – marginalized and excluded what? Populations?. Marginalization is defined as “a process whereby something or someone is pushed to the edge of a group and accorded lesser importance.” Social exclusion is defined as “ It involves the lack or denial of resources, rights, goods and services, and the inability to participate **in** the normal relationships and activities, available to the majority of people **in** a society, whether **in** economic, social, cultural or political

For peer review only

arenas." Operationally these two terminologies have been defined to be inclusive of the following keywords:

(Marginal[Title/Abstract])) OR (Marginalised[Title/Abstract])) OR (Marginalized[Title/Abstract])) OR (Refugee[Title/Abstract])) OR (Homeless[Title/Abstract])) OR (Migrant[Title/Abstract])) OR (financial catastrophe[Title/Abstract])) OR (poverty[Title/Abstract])) OR (conflict-affected[Title/Abstract])) OR (Under attack[Title/Abstract])) OR (Displaced[Title/Abstract])) OR (Temporary Accommodation[Title/Abstract])) OR (Temporary Settlement[Title/Abstract])) OR (Temporary Housing[Title/Abstract])) OR (Transitional Settlement[Title/Abstract])) OR (Transitional shelter[Title/Abstract])) OR (Emergency shelter[Title/Abstract])) OR (Emergency accommodation[Title/Abstract])) OR (Emergency housing[Title/Abstract])) OR (Makeshift shelter[Title/Abstract])) OR (Makeshift accommodation[Title/Abstract])) OR (Slash[Title/Abstract] AND Burn Cultivation[Title/Abstract])) OR (Shifting Cultivation[Title/Abstract])) OR (Feral[Title/Abstract])))

1.1.1.4. **Types of intervention:** Studies that will include interventions to improve or optimize the coverage of services, accessibility of the target population, and service utilization; we will include efficacy trials that include interventions only to improve specific health conditions within themes: feeding, nutrition, care practice, parenting, dental, wellbeing, and mental health, safe sleeping, physical activity, parenting support; inclusion of any bridging study will be decided jointly by the lead reviewers and the PI based on any added value)

1.1.1.5. **Types of comparators:** Studies that include marginality, conflict, or inequality for overall outcome, comparison, or sub-grouping.

1.1.1.6. **Types of outcome measures:** Studies that measure service output (coverage, access, utilization) and health (mortality, morbidity, quality-adjusted life years, disease/ disability-adjusted life years) and/or economic (efficiency, cost, return on investment);

For peer review only

1.1.1.7. **Time of Publications:** The dates searched will be from January 2000 to

FINAL SEARCH TERM (See Annexure for More Details)

((((((Child*[Title/Abstract]) OR (((Under five[Title/Abstract]) OR (Under 5[Title/Abstract])) OR (U5[Title/Abstract]))) OR (((Newborn[Title/Abstract]) OR (Infant[Title/Abstract])) OR (1000 days[Title/Abstract]))) AND (((Intervention*[Title/Abstract]) OR (Strateg*[Title/Abstract])) OR (Practice*[Title/Abstract]))) AND ((((((((((((((((((((((((Vulnerable[Title/Abstract]) OR (Marginal[Title/Abstract])) OR (Marginalised[Title/Abstract])) OR (Marginalized[Title/Abstract])) OR (Refugee[Title/Abstract])) OR (Homeless[Title/Abstract])) OR (Migrant[Title/Abstract])) OR (financial catastrophe[Title/Abstract])) OR (poverty[Title/Abstract])) OR (conflict-affected[Title/Abstract])) OR (Under attack[Title/Abstract])) OR (Displaced[Title/Abstract])) OR (Temporary Accommodation[Title/Abstract])) OR (Temporary Settlement[Title/Abstract])) OR (Temporary Housing[Title/Abstract])) OR (Transitional Settlement[Title/Abstract])) OR (Transitional shelter[Title/Abstract])) OR (Emergency shelter[Title/Abstract])) OR (Emergency accommodation[Title/Abstract])) OR (Emergency housing[Title/Abstract])) OR (Makeshift shelter[Title/Abstract])) OR (Makeshift accommodation[Title/Abstract])) OR (Slash[Title/Abstract] AND Burn Cultivation[Title/Abstract])) OR (Shifting Cultivation[Title/Abstract])) OR (Feral[Title/Abstract]))) AND (((((((Access[Title/Abstract]) OR (Accessibility[Title/Abstract])) OR (Inaccessible[Title/Abstract])) OR (inaccess[Title/Abstract]))) OR (((coverage[Title/Abstract]) OR (outreach[Title/Abstract])) OR (reach[Title/Abstract]))) OR ((((Use[Title/Abstract]) OR (Usage[Title/Abstract])) OR (Utilisation[Title/Abstract])) OR (Utilization[Title/Abstract]))) OR ((Available[Title/Abstract]) OR (Availability[Title/Abstract])))) AND (((((((Feeding[Title/Abstract]) OR (Nutrition[Title/Abstract])) OR (Care practice[Title/Abstract])) OR (Parenting[Title/Abstract])) OR (((dental[Title/Abstract]) OR (wellbeing[Title/Abstract])) OR (mental health[Title/Abstract]))) OR ((((safe sleeping[Title/Abstract]) OR (physical activity[Title/Abstract])) OR (parenting support[Title/Abstract])) OR (sleep hygiene[Title/Abstract]))) OR ((sleep practice[Title/Abstract]))))) AND (Andorra*[Title/Abstract] OR Antigua and Barbuda*[Title/Abstract] OR Australia*[Title/Abstract] OR Austria*[Title/Abstract] OR Bahrain*[Title/Abstract] OR Barbados*[Title/Abstract] OR Belgium*[Title/Abstract] OR Brunei*[Title/Abstract] OR Canada*[Title/Abstract] OR Chile*[Title/Abstract] OR Croatia*[Title/Abstract] OR Cyprus*[Title/Abstract] OR Czech Republic*[Title/Abstract] OR Denmark*[Title/Abstract] OR Estonia*[Title/Abstract] OR Finland*[Title/Abstract] OR France*[Title/Abstract] OR Germany*[Title/Abstract] OR Greece*[Title/Abstract] OR Hungary*[Title/Abstract] OR Iceland*[Title/Abstract] OR Israel*[Title/Abstract] OR Italy*[Title/Abstract] OR Japan*[Title/Abstract] OR South Korea*[Title/Abstract] OR Kuwait*[Title/Abstract] OR Latvia*[Title/Abstract] OR Liechtenstein*[Title/Abstract] OR Lithuania*[Title/Abstract] OR Luxembourg*[Title/Abstract] OR Monaco*[Title/Abstract] OR Nauru*[Title/Abstract] OR Netherlands*[Title/Abstract] OR New Zealand*[Title/Abstract] OR Norway*[Title/Abstract] OR Oman*[Title/Abstract] OR Palau*[Title/Abstract] OR Poland*[Title/Abstract] OR Portugal*[Title/Abstract] OR Qatar*[Title/Abstract] OR Saint Kitts and Nevis*[Title/Abstract] OR San Marino*[Title/Abstract] OR Seychelles*[Title/Abstract] OR Singapore*[Title/Abstract] OR Slovakia*[Title/Abstract] OR Slovenia*[Title/Abstract] OR Spain*[Title/Abstract] OR Sweden*[Title/Abstract] OR Switzerland*[Title/Abstract] OR Trinidad and Tobago*[Title/Abstract] OR United Arab Emirates*[Title/Abstract] OR United States*[Title/Abstract] OR Aruba*[Title/Abstract] OR Bermuda*[Title/Abstract] OR British Virgin Islands*[Title/Abstract] OR Cayman Islands*[Title/Abstract] OR Cook Islands*[Title/Abstract] OR Curaçao*[Title/Abstract] OR Faroe Islands*[Title/Abstract] OR French Polynesia*[Title/Abstract] OR Gibraltar*[Title/Abstract] OR Greenland*[Title/Abstract] OR Guam*[Title/Abstract] OR Hong Kong*[Title/Abstract] OR Isle of Man*[Title/Abstract] OR New Caledonia*[Title/Abstract] OR Northern Mariana Islands*[Title/Abstract] OR Puerto Rico*[Title/Abstract] OR Saint Martin*[Title/Abstract] OR Taiwan*[Title/Abstract] OR Turks and Caicos Islands*[Title/Abstract] OR U.S. Virgin Islands*[Title/Abstract])AND (("2000/01/01"[Date - Publication] : "3000"[Date - Publication]))

December 2021.

only

1.1.2. Search Database

1.1.2.1. **Electronic Database:**

- **i.** Databases: PubMed, MEDLINE, SCOPUS, The Cochrane Library (Cochrane Database of Systematic Reviews, Cochrane Central Register of Controlled Trials [CENTRAL]), and Google Scholar.
- **ii.** Clinical trial registries: ClinicalTrials.gov, Current Controlled Trials meta Register of Controlled Trials, International Clinical Trials Registry Platform (ICTRP) and.

1.1.2.2. **Grey literature:** Conference abstracts, Third Sector Reports.

1.1.2.3. **Correspondence:** Experts from Donor/ Philanthropic organizations and other key experts selected for an interview will be contacted for any additional published or unpublished work.

1.1.3. Relational and Citation Search:

We will conduct snowballing to screen cited articles of the 1st iteration of selections, followed by citations and related articles for each inclusion in PubMed, Google Scholar, and PubMed respectively. A maximum of three iterations will be undertaken.

1.1.4. DATA COLLECTION

1.1.4.1. **Screening Tool and Selection Method:** All articles will be extracted and compiled in a single spreadsheet. The spreadsheet will be equipped with duplicate study filters, and inclusion filters (inclusion criteria not managed through Search Term)

**Screening Filters**
**Geography:** High income countries (HICs) (Inclusion)<br>**Types of Emergency:** Grade 1, Grade 2, Grade 3 emergencies (WHO),<br>**Age of Target Population:** 0-1 (inclusion), 1-5 (inclusion), 5-14 (Exclusion)<br>**Interventions:** Interventions on access, coverage, utilization of health services; (inclusion)<br>**Measurements:** Output, outcome, impact (inclusion) input, process (exclusion)

and selection columns. Inclusion filters will be filled in independently by one reviewer (YT). Selection filters will be applied independently by two reviewers (YT and KS). All studies selected by two reviewers will be included. All studies rejected by two reviewers will be rejected. All studies having one inclusion will be marked as disputed selection. In case of dispute in selection third expert reviewer (ML) will make the final inclusion decision.

1.1.4.2. **Rounds of Screening:** Two round of screening will be undertaken– Title-Abstract (TiAb) and Full Text (FT).

1.1.4.3. **Preferred Reporting Items for Systematic Reviews and Meta-Analyses (PRISMA) Compliance:** The reasons for exclusion will be recorded and mapped in a flow chart as per PRISMA guidelines.

1.1.4.4. **Data Extraction:** A data extraction form (DEF) will be developed as per CEB Critically Appraised Topics (CAT) guidelines by KS and piloted by two authors (YT and KS) on the first 6 article inclusions (3 each). The piloting will essentially inform whether the DEF can extract necessary and sufficient information as per the objectives set. Any queries raised by the pilot will be reviewed by ML, NS, and ZP. The form will be amended based on the pilot. Two reviewers (YT and KS) will use the finalized form to extract data from full-text articles. If clarification is required, a domain expert (NS, YP, ML) will be consulted.

1.1.5. **Assessment of Risk of Bias (RoB):** RoB assessment will only be used for interventional studies for which results will be used for quantitative analysis or pooling. We will use multiple tools for risk of bias assessment based on the study type–

i. Systematic Reviews: **GRADE** for bias due to selective publication and selective non-reporting for systematic reviews

ii. Randomized Trials: **Cochrane RoB 2.0** to assess multiple sources of bias

iii. Non-randomised studies of interventions: **RoBANS** for bias due to selective non-reporting and bias in the selection of the reported result.

1.1.6. **Critical Appraisal for the included studies:** All studies will be critically appraised as per the CAT grading.

1.2. **Strategy for data synthesis**

The set of evidence will be categorized into qualitative, quantitative and mixed method. The qualitative evidence will be coded to find qualifiers through Word Cloud. The quantitative evidence will be subjected to descriptive analysis, followed by meta-analysis if possible.

1.3. **Referencing Software:** ENDNOTE

1.4. **Quality Assessment**

https://www.who.int/hrh/retention/annex1_grade_evidence_profiles.pdf

1.5. **Method Schema**

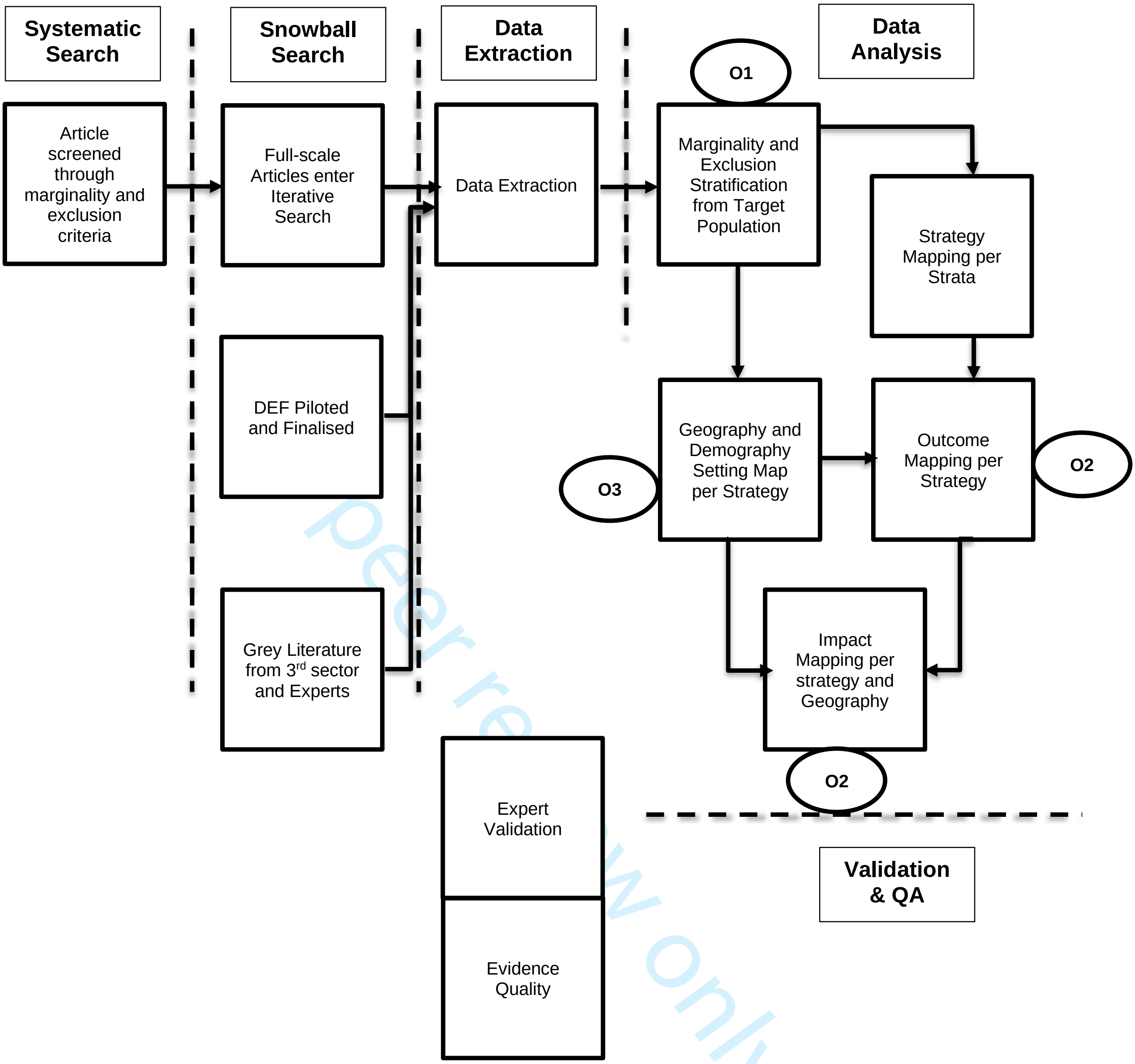

ANNEXURE

Search Strategy

Search number	Query	Sort By	Filters	Search Details	Results	Time
18	(("2000/01/01"[Date - Publication] : "3000/12/31"[Date - Publication])) AND ((((((("child*"[Title/Abstract]) OR ("under five"[Title/Abstract] OR "under 5"[Title/Abstract] OR "U5"[Title/Abstract])) OR ("Newborn"[Title/Abstract] OR "Infant"[Title/Abstract] OR "1000 days"[Title/Abstract])) AND ("intervention*"[Title/Abstract] OR "strategy*"[Title/Abstract] OR "practice*"[Title/Abstract])) AND ("Vulnerable"[Title/Abstract] OR "Marginal"[Title/Abstract] OR "Marginalised"[Title/Abstract] OR "Marginalized"[Title/Abstract] OR "Refugee"[Title/Abstract] OR "Homeless"[Title/Abstract] OR "Migrant"[Title/Abstract] OR "financial catastrophe"[Title/Abstract] OR "poverty"[Title/Abstract] OR "conflict-affected"[Title/Abstract] OR ("Under"[All Fields] OR "attack"[Title/Abstract]) OR "Displaced"[Title/Abstract] OR "temporary accommodation"[Title/Abstract] OR "temporary settlement"[Title/Abstract] OR "temporary housing"[Title/Abstract] OR (("transit"[All Fields] OR "transited"[All Fields] OR "transiting"[All Fields] OR "transition"[All Fields] OR "Transitional"[All Fields] OR "transitionals"[All Fields] OR "transitioned"[All Fields] OR "transitioning"[All Fields] OR "transitions"[All Fields] OR "transits"[All Fields]) OR "Settlement"[Title/Abstract]) OR "transitional shelter"[Title/Abstract] OR "emergency shelter"[Title/Abstract] OR "emergency accommodation"[Title/Abstract] OR "emergency housing"[Title/Abstract] OR ("Makeshift"[All Fields] OR "shelter"[Title/Abstract]) OR ("Makeshift"[All Fields] OR "Accommodation"[Title/Abstract]) OR ("Slash"[Title/Abstract] OR "burn cultivation"[Title/Abstract]) OR "shifting cultivation"[Title/Abstract] OR "Feral"[Title/Abstract)) AND (((("Access"[Title/Abstract] OR "Accessibility"[Title/Abstract] OR "Inaccessible"[Title/Abstract] OR "inaccess"[Title/Abstract]) OR ("coverage"[Title/Abstract] OR "outreach"[Title/Abstract] OR "reach"[Title/Abstract])) OR ("Use"[Title/Abstract] OR "Usage"[Title/Abstract] OR "Utilisation"[Title/Abstract] OR "Utilization"[Title/Abstract])) OR ("Available"[Title/Abstract] OR			("child*"[Title/Abstract] OR ("under five"[Title/Abstract] OR "under 5"[Title/Abstract] OR "U5"[Title/Abstract]) OR ("Newborn"[Title/Abstract] OR "Infant"[Title/Abstract] OR "1000 days"[Title/Abstract])) AND ("intervention*"[Title/Abstract] OR "strategy*"[Title/Abstract] OR "practice*"[Title/Abstract]) AND ("Vulnerable"[Title/Abstract] OR "Marginal"[Title/Abstract] OR "Marginalised"[Title/Abstract] OR "Marginalized"[Title/Abstract] OR "Refugee"[Title/Abstract] OR "Homeless"[Title/Abstract] OR "Migrant"[Title/Abstract] OR "financial catastrophe"[Title/Abstract] OR "poverty"[Title/Abstract] OR "conflict-affected"[Title/Abstract] OR ("Under"[All Fields] AND "attack"[Title/Abstract]) OR "Displaced"[Title/Abstract] OR "temporary accommodation"[Title/Abstract] OR "temporary settlement"[Title/Abstract] OR "temporary housing"[Title/Abstract] OR (("transit"[All Fields] OR "transited"[All Fields] OR "transiting"[All Fields] OR "transition"[All Fields] OR "Transitional"[All Fields] OR "transitionals"[All Fields] OR "transitioned"[All Fields] OR "transitioning"[All Fields] OR "transitions"[All Fields] OR "transits"[All Fields]) AND "Settlement"[Title/Abstract]) OR "transitional shelter"[Title/Abstract] OR "emergency shelter"[Title/Abstract] OR "emergency accommodation"[Title/Abstract] OR "emergency housing"[Title/Abstract] OR ("Makeshift"[All Fields] AND "shelter"[Title/Abstract]) OR ("Makeshift"[All Fields]	523	2:33:18

For peer review only

	"Availability"[Title/Abstract]))) AND ((Feeding*[Title/Abstract]) OR (nutrition*[Title/Abstract]) OR (care practice*[Title/Abstract]) OR (parenting*[Title/Abstract]) OR (dental*[Title/Abstract]) OR (wellbeing*[Title/Abstract]) OR (mental health*[Title/Abstract]) OR (safe sleeping*[Title/Abstract]) OR (physical activity*[Title/Abstract]) OR (parenting support*[Title/Abstract]) OR (sleep hygiene*[Title/Abstract]) OR (physical activity*[Title/Abstract]))) AND (Andorra*[Title/Abstract] OR Antigua and Barbuda*[Title/Abstract] OR Australia*[Title/Abstract] OR Austria*[Title/Abstract] OR Bahrain*[Title/Abstract] OR Barbados*[Title/Abstract] OR Belgium*[Title/Abstract] OR Brunei*[Title/Abstract] OR Canada*[Title/Abstract] OR Chile*[Title/Abstract] OR Croatia*[Title/Abstract] OR Cyprus*[Title/Abstract] OR Czech Republic*[Title/Abstract] OR Denmark*[Title/Abstract] OR Estonia*[Title/Abstract] OR Finland*[Title/Abstract] OR France*[Title/Abstract] OR Germany*[Title/Abstract] OR Greece*[Title/Abstract] OR Hungary*[Title/Abstract] OR Iceland*[Title/Abstract] OR Israel*[Title/Abstract] OR Italy*[Title/Abstract] OR Japan*[Title/Abstract] OR South Korea*[Title/Abstract] OR Kuwait*[Title/Abstract] OR Latvia*[Title/Abstract] OR Liechtenstein*[Title/Abstract] OR Lithuania*[Title/Abstract] OR Luxembourg*[Title/Abstract] OR Monaco*[Title/Abstract] OR Nauru*[Title/Abstract] OR Netherlands*[Title/Abstract] OR New Zealand*[Title/Abstract] OR Norway*[Title/Abstract] OR Oman*[Title/Abstract] OR Palau*[Title/Abstract] OR Poland*[Title/Abstract] OR Portugal*[Title/Abstract] OR Qatar*[Title/Abstract] OR Saint Kitts and Nevis*[Title/Abstract] OR San Marino*[Title/Abstract] OR Seychelles*[Title/Abstract] OR Singapore*[Title/Abstract] OR Slovakia*[Title/Abstract] OR Slovenia*[Title/Abstract] OR Spain*[Title/Abstract] OR Sweden*[Title/Abstract] OR Switzerland*[Title/Abstract] OR Trinidad and Tobago*[Title/Abstract] OR United Arab Emirates*[Title/Abstract] OR United States*[Title/Abstract] OR Aruba*[Title/Abstract] OR Bermuda*[Title/Abstract] OR British Virgin Islands*[Title/Abstract] OR Cayman Islands*[Title/Abstract] OR Cook Islands*[Title/Abstract] OR Curaçao*[Title/Abstract] OR Faroe Islands*[Title/Abstract] OR French Polynesia*[Title/Abstract] OR			AND "Accommodation"[Title/Abstract]) OR ("Slash"[Title/Abstract] AND "burn cultivation"[Title/Abstract]) OR "shifting cultivation"[Title/Abstract] OR "Feral"[Title/Abstract]) AND ("Access"[Title/Abstract] OR "Accessibility"[Title/Abstract] OR "Inaccessible"[Title/Abstract] OR "inaccess"[Title/Abstract] OR ("coverage"[Title/Abstract] OR "outreach"[Title/Abstract] OR "reach"[Title/Abstract]) OR ("Use"[Title/Abstract] OR "Usage"[Title/Abstract] OR "Utilisation"[Title/Abstract] OR "Utilization"[Title/Abstract]) OR ("Available"[Title/Abstract] OR "Availability"[Title/Abstract])) AND (Feeding*[Title/Abstract])) OR (nutrition*[Title/Abstract])) OR (care practice*[Title/Abstract])) OR (parenting*[Title/Abstract])) OR (dental*[Title/Abstract])) OR (wellbeing*[Title/Abstract])) OR (mental health*[Title/Abstract])) OR (safe sleeping*[Title/Abstract])) OR (physical activity*[Title/Abstract])) OR (parenting support*[Title/Abstract])) OR (sleep hygiene*[Title/Abstract])) OR (physical activity*[Title/Abstract]) AND (Andorra*[Title/Abstract] OR Antigua and Barbuda*[Title/Abstract] OR Australia*[Title/Abstract] OR Austria*[Title/Abstract] OR Bahrain*[Title/Abstract] OR Barbados*[Title/Abstract] OR Belgium*[Title/Abstract] OR Brunei*[Title/Abstract] OR Canada*[Title/Abstract] OR Chile*[Title/Abstract] OR Croatia*[Title/Abstract] OR Cyprus*[Title/Abstract] OR Czech Republic*[Title/Abstract] OR		

For peer review only

	Gibraltar*[Title/Abstract] OR Greenland*[Title/Abstract] OR Guam*[Title/Abstract] OR Hong Kong*[Title/Abstract] OR Isle of Man*[Title/Abstract] OR New Caledonia*[Title/Abstract] OR Northern Mariana Islands*[Title/Abstract] OR Puerto Rico*[Title/Abstract] OR Saint Martin*[Title/Abstract] OR Taiwan*[Title/Abstract] OR Turks and Caicos Islands*[Title/Abstract] OR U.S. Virgin Islands*[Title/Abstract]))			Denmark*[Title/Abstract] OR Estonia*[Title/Abstract] OR Finland*[Title/Abstract] OR France*[Title/Abstract] OR Germany*[Title/Abstract] OR Greece*[Title/Abstract] OR Hungary*[Title/Abstract] OR Iceland*[Title/Abstract] OR Israel*[Title/Abstract] OR Italy*[Title/Abstract] OR Japan*[Title/Abstract] OR South Korea*[Title/Abstract] OR Kuwait*[Title/Abstract] OR Latvia*[Title/Abstract] OR Liechtenstein*[Title/Abstract] OR Lithuania*[Title/Abstract] OR Luxembourg*[Title/Abstract] OR Monaco*[Title/Abstract] OR Nauru*[Title/Abstract] OR Netherlands*[Title/Abstract] OR New Zealand*[Title/Abstract] OR Norway*[Title/Abstract] OR Oman*[Title/Abstract] OR Palau*[Title/Abstract] OR Poland*[Title/Abstract] OR Portugal*[Title/Abstract] OR Qatar*[Title/Abstract] OR Saint Kitts and Nevis*[Title/Abstract] OR San Marino*[Title/Abstract] OR Seychelles*[Title/Abstract] OR Singapore*[Title/Abstract] OR Slovakia*[Title/Abstract] OR Slovenia*[Title/Abstract] OR Spain*[Title/Abstract] OR Sweden*[Title/Abstract] OR Switzerland*[Title/Abstract] OR Trinidad and Tobago*[Title/Abstract] OR United Arab Emirates*[Title/Abstract] OR United States*[Title/Abstract] OR Aruba*[Title/Abstract] OR Bermuda*[Title/Abstract] OR British Virgin Islands*[Title/Abstract] OR Cayman Islands*[Title/Abstract] OR Cook Islands*[Title/Abstract] OR Curaçao*[Title/Abstract] OR Faroe Islands*[Title/Abstract] OR French Polynesia*[Title/Abstract] OR Gibraltar*[Title/Abstract] OR Greenland*[Title/Abstract] OR Guam*[Title/Abstract] OR Hong Kong*[Title/Abstract] OR		

For peer review only

				Isle of Man*[Title/Abstract] OR New Caledonia*[Title/Abstract] OR Northern Mariana Islands*[Title/Abstract] OR Puerto Rico*[Title/Abstract] OR Saint Martin*[Title/Abstract] OR Taiwan*[Title/Abstract] OR Turks and Caicos Islands*[Title/Abstract] OR U.S. Virgin Islands*[Title/Abstract]))AN D 2010/01/01:3000/12/31[Dat e - Publication]		

For peer review only

17	(((((("child*"[Title/Abstract]) OR ("under five"[Title/Abstract] OR "under 5"[Title/Abstract] OR "U5"[Title/Abstract])) OR ("Newborn"[Title/Abstract] OR "Infant"[Title/Abstract] OR "1000 days"[Title/Abstract])) AND ("intervention*"[Title/Abstract] OR "strategy*"[Title/Abstract] OR "practice*"[Title/Abstract])) AND ("Vulnerable"[Title/Abstract] OR "Marginal"[Title/Abstract] OR "Marginalised"[Title/Abstract] OR "Marginalized"[Title/Abstract] OR "Refugee"[Title/Abstract] OR "Homeless"[Title/Abstract] OR "Migrant"[Title/Abstract] OR "financial catastrophe"[Title/Abstract] OR "poverty"[Title/Abstract] OR "conflict-affected"[Title/Abstract] OR ("Under"[All Fields] OR "attack"[Title/Abstract]) OR "Displaced"[Title/Abstract] OR "temporary accommodation"[Title/Abstract] OR "temporary settlement"[Title/Abstract] OR "temporary housing"[Title/Abstract] OR (("transit"[All Fields] OR "transited"[All Fields] OR "transiting"[All Fields] OR "transition"[All Fields] OR "Transitional"[All Fields] OR "transitionals"[All Fields] OR "transitioned"[All Fields] OR "transitioning"[All Fields] OR "transitions"[All Fields] OR "transits"[All Fields]) OR "Settlement"[Title/Abstract]) OR "transitional shelter"[Title/Abstract] OR "emergency shelter"[Title/Abstract] OR "emergency accommodation"[Title/Abstract] OR "emergency housing"[Title/Abstract] OR ("Makeshift"[All Fields] OR "shelter"[Title/Abstract]) OR ("Makeshift"[All Fields] OR "Accommodation"[Title/Abstract]) OR ("Slash"[Title/Abstract] OR "burn cultivation"[Title/Abstract]) OR "shifting cultivation"[Title/Abstract] OR "Feral"[Title/Abstract)) AND (((("Access"[Title/Abstract] OR "Accessibility"[Title/Abstract] OR "Inaccessible"[Title/Abstract] OR "inaccess"[Title/Abstract]) OR ("coverage"[Title/Abstract] OR "outreach"[Title/Abstract] OR "reach"[Title/Abstract])) OR ("Use"[Title/Abstract] OR "Usage"[Title/Abstract] OR "Utilisation"[Title/Abstract] OR "Utilization"[Title/Abstract])) OR ("Available"[Title/Abstract] OR "Availability"[Title/Abstract]))) AND ((Feeding*[Title/Abstract]) OR (nutrition*[Title/Abstract]) OR (care practice*[Title/Abstract]) OR (parenting*[Title/Abstract]) OR (dental*[Title/Abstract]) OR (wellbeing*[Title/Abstract]) OR (mental health*[Title/Abstract]) OR (safe sleeping*[Title/Abstract]) OR (physical activity*[Title/Abstract]) OR			(("child*"[Title/Abstract] OR ("under five"[Title/Abstract] OR "under 5"[Title/Abstract] OR "U5"[Title/Abstract]) OR ("Newborn"[Title/Abstract] OR "Infant"[Title/Abstract] OR "1000 days"[Title/Abstract])) AND ("intervention*"[Title/Abstract] OR "strategy*"[Title/Abstract] OR "practice*"[Title/Abstract]) AND ("Vulnerable"[Title/Abstract] OR "Marginal"[Title/Abstract] OR "Marginalised"[Title/Abstract] OR "Marginalized"[Title/Abstract] OR "Refugee"[Title/Abstract] OR "Homeless"[Title/Abstract] OR "Migrant"[Title/Abstract] OR "financial catastrophe"[Title/Abstract] OR "poverty"[Title/Abstract] OR "conflict-affected"[Title/Abstract] OR ("Under"[All Fields] AND "attack"[Title/Abstract]) OR "Displaced"[Title/Abstract] OR "temporary accommodation"[Title/Abstract] OR "temporary settlement"[Title/Abstract] OR "temporary housing"[Title/Abstract] OR (("transit"[All Fields] OR "transited"[All Fields] OR "transiting"[All Fields] OR "transition"[All Fields] OR "Transitional"[All Fields] OR "transitionals"[All Fields] OR "transitioned"[All Fields] OR "transitioning"[All Fields] OR "transitions"[All Fields] OR "transits"[All Fields]) AND "Settlement"[Title/Abstract]) OR "transitional shelter"[Title/Abstract] OR "emergency shelter"[Title/Abstract] OR "emergency accommodation"[Title/Abstract] OR "emergency housing"[Title/Abstract] OR ("Makeshift"[All Fields] AND "shelter"[Title/Abstract]) OR ("Makeshift"[All Fields] AND "Accommodation"[Title/Abstract]) OR ("Slash"[Title/Abstract] AND "burn cultivation"[Title/Abstract]) OR "shifting cultivation"[Title/Abstract]	642	2:32:48

For peer review only

	(parenting support*[Title/Abstract]) OR (sleep hygiene*[Title/Abstract]) OR (physical activity*[Title/Abstract]))) AND (Andorra*[Title/Abstract] OR Antigua and Barbuda*[Title/Abstract] OR Australia*[Title/Abstract] OR Austria*[Title/Abstract] OR Bahrain*[Title/Abstract] OR Barbados*[Title/Abstract] OR Belgium*[Title/Abstract] OR Brunei*[Title/Abstract] OR Canada*[Title/Abstract] OR Chile*[Title/Abstract] OR Croatia*[Title/Abstract] OR Cyprus*[Title/Abstract] OR Czech Republic*[Title/Abstract] OR Denmark*[Title/Abstract] OR Estonia*[Title/Abstract] OR Finland*[Title/Abstract] OR France*[Title/Abstract] OR Germany*[Title/Abstract] OR Greece*[Title/Abstract] OR Hungary*[Title/Abstract] OR Iceland*[Title/Abstract] OR Israel*[Title/Abstract] OR Italy*[Title/Abstract] OR Japan*[Title/Abstract] OR South Korea*[Title/Abstract] OR Kuwait*[Title/Abstract] OR Latvia*[Title/Abstract] OR Liechtenstein*[Title/Abstract] OR Lithuania*[Title/Abstract] OR Luxembourg*[Title/Abstract] OR Monaco*[Title/Abstract] OR Nauru*[Title/Abstract] OR Netherlands*[Title/Abstract] OR New Zealand*[Title/Abstract] OR Norway*[Title/Abstract] OR Oman*[Title/Abstract] OR Palau*[Title/Abstract] OR Poland*[Title/Abstract] OR Portugal*[Title/Abstract] OR Qatar*[Title/Abstract] OR Saint Kitts and Nevis*[Title/Abstract] OR San Marino*[Title/Abstract] OR Seychelles*[Title/Abstract] OR Singapore*[Title/Abstract] OR Slovakia*[Title/Abstract] OR Slovenia*[Title/Abstract] OR Spain*[Title/Abstract] OR Sweden*[Title/Abstract] OR Switzerland*[Title/Abstract] OR Trinidad and Tobago*[Title/Abstract] OR United Arab Emirates*[Title/Abstract] OR United States*[Title/Abstract] OR Aruba*[Title/Abstract] OR Bermuda*[Title/Abstract] OR British Virgin Islands*[Title/Abstract] OR Cayman Islands*[Title/Abstract] OR Cook Islands*[Title/Abstract] OR Curaçao*[Title/Abstract] OR Faroe Islands*[Title/Abstract] OR French Polynesia*[Title/Abstract] OR Gibraltar*[Title/Abstract] OR Greenland*[Title/Abstract] OR Guam*[Title/Abstract] OR Hong Kong*[Title/Abstract] OR Isle of Man*[Title/Abstract] OR New Caledonia*[Title/Abstract] OR Northern Mariana Islands*[Title/Abstract] OR Puerto Rico*[Title/Abstract] OR Saint Martin*[Title/Abstract] OR			OR "Feral"[Title/Abstract]) AND ("Access"[Title/Abstract] OR "Accessibility"[Title/Abstract] OR "Inaccessible"[Title/Abstract] OR "inaccess"[Title/Abstract] OR ("coverage"[Title/Abstract] OR "outreach"[Title/Abstract] OR "reach"[Title/Abstract]) OR ("Use"[Title/Abstract] OR "Usage"[Title/Abstract] OR "Utilisation"[Title/Abstract] OR "Utilization"[Title/Abstract]) OR ("Available"[Title/Abstract] OR "Availability"[Title/Abstract]))) AND (Feeding*[Title/Abstract])) OR (nutrition*[Title/Abstract])) OR (care practice*[Title/Abstract])) OR (parenting*[Title/Abstract])) OR (dental*[Title/Abstract])) OR (wellbeing*[Title/Abstract])) OR (mental health*[Title/Abstract])) OR (safe sleeping*[Title/Abstract])) OR (physical activity*[Title/Abstract])) OR (parenting support*[Title/Abstract])) OR (sleep hygiene*[Title/Abstract])) OR (physical activity*[Title/Abstract]) AND (Andorra*[Title/Abstract] OR Antigua and Barbuda*[Title/Abstract] OR Australia*[Title/Abstract] OR Austria*[Title/Abstract] OR Bahrain*[Title/Abstract] OR Barbados*[Title/Abstract] OR Belgium*[Title/Abstract] OR Brunei*[Title/Abstract] OR Canada*[Title/Abstract] OR Chile*[Title/Abstract] OR Croatia*[Title/Abstract] OR Cyprus*[Title/Abstract] OR Czech Republic*[Title/Abstract] OR Denmark*[Title/Abstract] OR Estonia*[Title/Abstract] OR Finland*[Title/Abstract] OR France*[Title/Abstract] OR Germany*[Title/Abstract] OR Greece*[Title/Abstract] OR		

	Taiwan*[Title/Abstract] OR Turks and Caicos Islands*[Title/Abstract] OR U.S. Virgin Islands*[Title/Abstract])			Hungary*[Title/Abstract] OR Iceland*[Title/Abstract] OR Israel*[Title/Abstract] OR Italy*[Title/Abstract] OR Japan*[Title/Abstract] OR South Korea*[Title/Abstract] OR Kuwait*[Title/Abstract] OR Latvia*[Title/Abstract] OR Liechtenstein*[Title/Abstract] OR Lithuania*[Title/Abstract] OR Luxembourg*[Title/Abstract] OR Monaco*[Title/Abstract] OR Nauru*[Title/Abstract] OR Netherlands*[Title/Abstract] OR New Zealand*[Title/Abstract] OR Norway*[Title/Abstract] OR Oman*[Title/Abstract] OR Palau*[Title/Abstract] OR Poland*[Title/Abstract] OR Portugal*[Title/Abstract] OR Qatar*[Title/Abstract] OR Saint Kitts and Nevis*[Title/Abstract] OR San Marino*[Title/Abstract] OR Seychelles*[Title/Abstract] OR Singapore*[Title/Abstract] OR Slovakia*[Title/Abstract] OR Slovenia*[Title/Abstract] OR Spain*[Title/Abstract] OR Sweden*[Title/Abstract] OR Switzerland*[Title/Abstract] OR Trinidad and Tobago*[Title/Abstract] OR United Arab Emirates*[Title/Abstract] OR United States*[Title/Abstract] OR Aruba*[Title/Abstract] OR Bermuda*[Title/Abstract] OR British Virgin Islands*[Title/Abstract] OR Cayman Islands*[Title/Abstract] OR Cook Islands*[Title/Abstract] OR Curaçao*[Title/Abstract] OR Faroe Islands*[Title/Abstract] OR French Polynesia*[Title/Abstract] OR Gibraltar*[Title/Abstract] OR Greenland*[Title/Abstract] OR Guam*[Title/Abstract] OR Hong Kong*[Title/Abstract] OR Isle of Man*[Title/Abstract] OR New Caledonia*[Title/Abstract] OR Northern Mariana Islands*[Title/Abstract] OR Puerto Rico*[Title/Abstract] OR Saint Martin*[Title/Abstract] OR		

For peer review only

				Taiwan*[Title/Abstract] OR Turks and Caicos Islands*[Title/Abstract] OR U.S. Virgin Islands*[Title/Abstract]))		

For peer review only

16	(((((Child*[Title/Abstract]) OR (((Under five[Title/Abstract]) OR (Under 5[Title/Abstract])) OR (U5[Title/Abstract]))) OR (((Newborn[Title/Abstract]) OR (Infant[Title/Abstract])) OR (1000 days[Title/Abstract]))) AND (((Intervention*[Title/Abstract]) OR (Strateg*[Title/Abstract])) OR (Practice*[Title/Abstract]))) AND (((((((((((((((((((((((((Vulnerable[Title/Abstract]) OR (Marginal[Title/Abstract])) OR (Marginalised[Title/Abstract])) OR (Marginalized[Title/Abstract])) OR (Refugee[Title/Abstract])) OR (Homeless[Title/Abstract])) OR (Migrant[Title/Abstract])) OR (financial catastrophe[Title/Abstract])) OR (poverty[Title/Abstract])) OR (conflict-affected[Title/Abstract])) OR (Under attack[Title/Abstract])) OR (Displaced[Title/Abstract])) OR (Temporary Accommodation[Title/Abstract])) OR (Temporary Settlement[Title/Abstract])) OR (Temporary Housing[Title/Abstract])) OR (Transitional Settlement[Title/Abstract])) OR (Transitional shelter[Title/Abstract])) OR (Emergency shelter[Title/Abstract])) OR (Emergency accommodation[Title/Abstract])) OR (Emergency housing[Title/Abstract])) OR (Makeshift shelter[Title/Abstract])) OR (Makeshift accommodation[Title/Abstract])) OR (Slash[Title/Abstract] AND Burn Cultivation[Title/Abstract])) OR (Shifting Cultivation[Title/Abstract])) OR (Feral[Title/Abstract]))) AND (((((((Access[Title/Abstract]) OR (Accessibility[Title/Abstract])) OR (Inaccessible[Title/Abstract])) OR (inaccess[Title/Abstract])) OR (((coverage[Title/Abstract]) OR (outreach[Title/Abstract])) OR (reach[Title/Abstract]))) OR ((((Use[Title/Abstract]) OR (Usage[Title/Abstract])) OR (Utilisation[Title/Abstract])) OR (Utilization[Title/Abstract]))) OR ((Available[Title/Abstract]) OR (Availability[Title/Abstract])))) AND (((((((Feeding[Title/Abstract]) OR (Nutrition[Title/Abstract])) OR (Care practice[Title/Abstract])) OR (Parenting[Title/Abstract])) OR (((dental[Title/Abstract]) OR (wellbeing[Title/Abstract])) OR (mental health[Title/Abstract]))) OR ((((safe sleeping[Title/Abstract]) OR (physical activity[Title/Abstract])) OR (parenting support[Title/Abstract])) OR (sleep hygiene[Title/Abstract]))) OR ((sleep practice[Title/Abstract])))))			("child*"[Title/Abstract] OR ("under five"[Title/Abstract] OR "under 5"[Title/Abstract] OR "U5"[Title/Abstract]) OR ("Newborn"[Title/Abstract] OR "Infant"[Title/Abstract] OR "1000 days"[Title/Abstract])) AND ("intervention*"[Title/Abstract] OR "strategy*"[Title/Abstract] OR "practice*"[Title/Abstract]) AND ("Vulnerable"[Title/Abstract] OR "Marginal"[Title/Abstract] OR "Marginalised"[Title/Abstract] OR "Marginalized"[Title/Abstract] OR "Refugee"[Title/Abstract] OR "Homeless"[Title/Abstract] OR "Migrant"[Title/Abstract] OR "financial catastrophe"[Title/Abstract] OR "poverty"[Title/Abstract] OR "conflict-affected"[Title/Abstract] OR ("Under"[All Fields] AND "attack"[Title/Abstract]) OR "Displaced"[Title/Abstract] OR "temporary accommodation"[Title/Abstract] OR "temporary settlement"[Title/Abstract] OR "temporary housing"[Title/Abstract] OR (("transit"[All Fields] OR "transited"[All Fields] OR "transiting"[All Fields] OR "transition"[All Fields] OR "Transitional"[All Fields] OR "transitionals"[All Fields] OR "transitioned"[All Fields] OR "transitioning"[All Fields] OR "transitions"[All Fields] OR "transits"[All Fields]) AND "Settlement"[Title/Abstract]) OR "transitional shelter"[Title/Abstract] OR "emergency shelter"[Title/Abstract] OR "emergency accommodation"[Title/Abstract] OR "emergency housing"[Title/Abstract] OR ("Makeshift"[All Fields] AND "shelter"[Title/Abstract]) OR ("Makeshift"[All Fields] AND "Accommodation"[Title/Abstract]) OR ("Slash"[Title/Abstract] AND "burn cultivation"[Title/Abstract]) OR "shifting cultivation"[Title/Abstract]		

For peer review only

				OR "Feral"[Title/Abstract]) AND ("Access"[Title/Abstract] OR "Accessibility"[Title/Abstract] OR "Inaccessible"[Title/Abstract] OR "inaccess"[Title/Abstract] OR ("coverage"[Title/Abstract] OR "outreach"[Title/Abstract] OR "reach"[Title/Abstract]) OR ("Use"[Title/Abstract] OR "Usage"[Title/Abstract] OR "Utilisation"[Title/Abstract] OR "Utilization"[Title/Abstract]) OR ("Available"[Title/Abstract] OR "Availability"[Title/Abstract])) AND (Feeding*[Title/Abstract])) OR (nutrition*[Title/Abstract])) OR (care practice*[Title/Abstract])) OR (parenting*[Title/Abstract])) OR (dental*[Title/Abstract])) OR (wellbeing*[Title/Abstract])) OR (mental health*[Title/Abstract])) OR (safe sleeping*[Title/Abstract])) OR (physical activity*[Title/Abstract])) OR (parenting support*[Title/Abstract])) OR (sleep hygiene*[Title/Abstract])) OR (physical activity*[Title/Abstract])		

For peer review only

15	(((((Child*[Title/Abstract]) OR (((Under five[Title/Abstract]) OR (Under 5[Title/Abstract])) OR (U5[Title/Abstract]))) OR (((Newborn[Title/Abstract]) OR (Infant[Title/Abstract])) OR (1000 days[Title/Abstract]))) AND (((Intervention*[Title/Abstract]) OR (Strateg*[Title/Abstract])) OR (Practice*[Title/Abstract]))) AND (((((((((((((((((((((((((Vulnerable[Title/Abstract]) OR (Marginal[Title/Abstract])) OR (Marginalised[Title/Abstract])) OR (Marginalized[Title/Abstract])) OR (Refugee[Title/Abstract])) OR (Homeless[Title/Abstract])) OR (Migrant[Title/Abstract])) OR (financial catastrophe[Title/Abstract])) OR (poverty[Title/Abstract])) OR (conflict-affected[Title/Abstract])) OR (Under attack[Title/Abstract])) OR (Displaced[Title/Abstract])) OR (Temporary Accommodation[Title/Abstract])) OR (Temporary Settlement[Title/Abstract])) OR (Temporary Housing[Title/Abstract])) OR (Transitional Settlement[Title/Abstract])) OR (Transitional shelter[Title/Abstract])) OR (Emergency shelter[Title/Abstract])) OR (Emergency accommodation[Title/Abstract])) OR (Emergency housing[Title/Abstract])) OR (Makeshift shelter[Title/Abstract])) OR (Makeshift accommodation[Title/Abstract])) OR (Slash[Title/Abstract] AND Burn Cultivation[Title/Abstract])) OR (Shifting Cultivation[Title/Abstract])) OR (Feral[Title/Abstract]))) AND (((((((Access[Title/Abstract]) OR (Accessibility[Title/Abstract])) OR (Inaccessible[Title/Abstract])) OR (inaccess[Title/Abstract])) OR (((coverage[Title/Abstract]) OR (outreach[Title/Abstract])) OR (reach[Title/Abstract]))) OR ((((Use[Title/Abstract]) OR (Usage[Title/Abstract])) OR (Utilisation[Title/Abstract])) OR (Utilization[Title/Abstract]))) OR ((Available[Title/Abstract]) OR (Availability[Title/Abstract])))			("child*"[Title/Abstract] OR ("under five"[Title/Abstract] OR "under 5"[Title/Abstract] OR "U5"[Title/Abstract]) OR ("Newborn"[Title/Abstract] OR "Infant"[Title/Abstract] OR "1000 days"[Title/Abstract])) AND ("intervention*"[Title/Abstract] OR "strategy*"[Title/Abstract] OR "practice*"[Title/Abstract]) AND ("Vulnerable"[Title/Abstract] OR "Marginal"[Title/Abstract] OR "Marginalised"[Title/Abstract] OR "Marginalized"[Title/Abstract] OR "Refugee"[Title/Abstract] OR "Homeless"[Title/Abstract] OR "Migrant"[Title/Abstract] OR "financial catastrophe"[Title/Abstract] OR "poverty"[Title/Abstract] OR "conflict-affected"[Title/Abstract] OR ("Under"[All Fields] AND "attack"[Title/Abstract]) OR "Displaced"[Title/Abstract] OR "temporary accommodation"[Title/Abstract] OR "temporary settlement"[Title/Abstract] OR "temporary housing"[Title/Abstract] OR (("transit"[All Fields] OR "transited"[All Fields] OR "transiting"[All Fields] OR "transition"[All Fields] OR "Transitional"[All Fields] OR "transitionals"[All Fields] OR "transitioned"[All Fields] OR "transitioning"[All Fields] OR "transitions"[All Fields] OR "transits"[All Fields]) AND "Settlement"[Title/Abstract]) OR "transitional shelter"[Title/Abstract] OR "emergency shelter"[Title/Abstract] OR "emergency accommodation"[Title/Abstract] OR "emergency housing"[Title/Abstract] OR ("Makeshift"[All Fields] AND "shelter"[Title/Abstract]) OR ("Makeshift"[All Fields] AND "Accommodation"[Title/Abstract]) OR ("Slash"[Title/Abstract] AND "burn cultivation"[Title/Abstract]) OR "shifting cultivation"[Title/Abstract]	14,828	2:31:46

				OR "Feral"[Title/Abstract]) AND ("Access"[Title/Abstract] OR "Accessibility"[Title/Abstract] OR "Inaccessible"[Title/Abstract] OR "inaccess"[Title/Abstract] OR ("coverage"[Title/Abstract] OR "outreach"[Title/Abstract] OR "reach"[Title/Abstract]) OR ("Use"[Title/Abstract] OR "Usage"[Title/Abstract] OR "Utilisation"[Title/Abstract] OR "Utilization"[Title/Abstract]) OR ("Available"[Title/Abstract] OR "Availability"[Title/Abstract]))		
14	((((((Access[Title/Abstract]) OR (Accessibility[Title/Abstract])) OR (Inaccessible[Title/Abstract])) OR (inaccess[Title/Abstract])) OR (((coverage[Title/Abstract]) OR (outreach[Title/Abstract])) OR (reach[Title/Abstract]))) OR ((((Use[Title/Abstract]) OR (Usage[Title/Abstract])) OR (Utilisation[Title/Abstract])) OR (Utilization[Title/Abstract]))) OR ((Available[Title/Abstract]) OR (Availability[Title/Abstract]))			"Access"[Title/Abstract] OR "Accessibility"[Title/Abstract] OR "Inaccessible"[Title/Abstract] OR "inaccess"[Title/Abstract] OR "coverage"[Title/Abstract] OR "outreach"[Title/Abstract] OR "reach"[Title/Abstract] OR "Use"[Title/Abstract] OR "Usage"[Title/Abstract] OR "Utilisation"[Title/Abstract] OR "Utilization"[Title/Abstract] OR "Available"[Title/Abstract] OR "Availability"[Title/Abstract]	4,907,175	2:31:24
13	(Available[Title/Abstract]) OR (Availability[Title/Abstract])			"Available"[Title/Abstract] OR "Availability"[Title/Abstract]	1,358,789	2:30:57

For peer review only

12	(((Use[Title/Abstract]) OR (Usage[Title/Abstract])) OR (Utilisation[Title/Abstract])) OR (Utilization[Title/Abstract])			"Use"[Title/Abstract] OR "Usage"[Title/Abstract] OR "Utilisation"[Title/Abstract] OR "Utilization"[Title/Abstract]	3,385,414	2:30:36
11	((coverage[Title/Abstract]) OR (outreach[Title/Abstract])) OR (reach[Title/Abstract])			"coverage"[Title/Abstract] OR "outreach"[Title/Abstract] OR "reach"[Title/Abstract]	325,002	2:28:26
10	(((Access[Title/Abstract]) OR (Accessibility[Title/Abstract])) OR (Inaccessible[Title/Abstract])) OR (inaccess[Title/Abstract])			"Access"[Title/Abstract] OR "Accessibility"[Title/Abstract] OR "Inaccessible"[Title/Abstract] OR "inaccess"[Title/Abstract]	424,084	2:27:31
9	((((Child*[Title/Abstract]) OR (((Under five[Title/Abstract]) OR (Under 5[Title/Abstract])) OR (U5[Title/Abstract]))) OR (((Newborn[Title/Abstract]) OR (Infant[Title/Abstract])) OR (1000 days[Title/Abstract]))) AND (((Intervention*[Title/Abstract]) OR (Strateg*[Title/Abstract])) OR (Practice*[Title/Abstract]))) AND ((((((((((((((((((((((((Vulnerable[Title/Abstract]) OR (Marginal[Title/Abstract])) OR (Marginalised[Title/Abstract])) OR (Marginalized[Title/Abstract])) OR (Refugee[Title/Abstract])) OR (Homeless[Title/Abstract])) OR (Migrant[Title/Abstract])) OR (financial catastrophe[Title/Abstract])) OR (poverty[Title/Abstract])) OR (conflict-affected[Title/Abstract])) OR (Under attack[Title/Abstract])) OR (Displaced[Title/Abstract])) OR (Temporary Accommodation[Title/Abstract])) OR (Temporary Settlement[Title/Abstract])) OR (Temporary Housing[Title/Abstract])) OR (Transitional Settlement[Title/Abstract])) OR (Transitional shelter[Title/Abstract])) OR (Emergency shelter[Title/Abstract])) OR (Emergency accommodation[Title/Abstract])) OR (Emergency housing[Title/Abstract])) OR (Makeshift shelter[Title/Abstract])) OR (Makeshift accommodation[Title/Abstract])) OR (Slash[Title/Abstract] AND Burn Cultivation[Title/Abstract])) OR (Shifting Cultivation[Title/Abstract])) OR (Feral[Title/Abstract]))			("child*"[Title/Abstract] OR ("under five"[Title/Abstract] OR "under 5"[Title/Abstract] OR "U5"[Title/Abstract]) OR ("Newborn"[Title/Abstract] OR "Infant"[Title/Abstract] OR "1000 days"[Title/Abstract])) AND ("intervention*"[Title/Abstract] OR "strategy*"[Title/Abstract] OR "practice*"[Title/Abstract]) AND ("Vulnerable"[Title/Abstract] OR "Marginal"[Title/Abstract] OR "Marginalised"[Title/Abstract] OR "Marginalized"[Title/Abstract] OR "Refugee"[Title/Abstract] OR "Homeless"[Title/Abstract] OR "Migrant"[Title/Abstract] OR "financial catastrophe"[Title/Abstract] OR "poverty"[Title/Abstract] OR "conflict-affected"[Title/Abstract] OR ("Under"[All Fields] AND "attack"[Title/Abstract]) OR "Displaced"[Title/Abstract] OR "temporary accommodation"[Title/Abstract] OR "temporary settlement"[Title/Abstract] OR "temporary housing"[Title/Abstract] OR (("transit"[All Fields] OR "transited"[All Fields] OR "transiting"[All Fields] OR "transition"[All Fields] OR "Transitional"[All Fields] OR "transitionals"[All Fields] OR "transitioned"[All Fields] OR "transitioning"[All Fields] OR "transitions"[All Fields] OR "transits"[All	35,906	2:24:50

For peer review only

				Fields]) AND "Settlement"[Title/Abstract]) OR "transitional shelter"[Title/Abstract] OR "emergency shelter"[Title/Abstract] OR "emergency accommodation"[Title/Abstract] OR "emergency housing"[Title/Abstract] OR ("Makeshift"[All Fields] AND "shelter"[Title/Abstract]) OR ("Makeshift"[All Fields] AND "Accommodation"[Title/Abstract]) OR ("Slash"[Title/Abstract] AND "burn cultivation"[Title/Abstract]) OR "shifting cultivation"[Title/Abstract] OR "Feral"[Title/Abstract])		

For peer review only

8	(((((((((((((((((((((((Vulnerable[Title/Abstract]) OR (Marginal[Title/Abstract])) OR (Marginalised[Title/Abstract])) OR (Marginalized[Title/Abstract])) OR (Refugee[Title/Abstract])) OR (Homeless[Title/Abstract])) OR (Migrant[Title/Abstract])) OR (financial catastrophe[Title/Abstract])) OR (poverty[Title/Abstract])) OR (conflict-affected[Title/Abstract])) OR (Under attack[Title/Abstract])) OR (Displaced[Title/Abstract])) OR (Temporary Accommodation[Title/Abstract])) OR (Temporary Settlement[Title/Abstract])) OR (Temporary Housing[Title/Abstract])) OR (Transitional Settlement[Title/Abstract])) OR (Transitional shelter[Title/Abstract])) OR (Emergency shelter[Title/Abstract])) OR (Emergency accommodation[Title/Abstract])) OR (Emergency housing[Title/Abstract])) OR (Makeshift shelter[Title/Abstract])) OR (Makeshift accommodation[Title/Abstract])) OR (Slash[Title/Abstract] AND Burn Cultivation[Title/Abstract])) OR (Shifting Cultivation[Title/Abstract])) OR (Feral[Title/Abstract])			"Vulnerable"[Title/Abstract] OR "Marginal"[Title/Abstract] OR "Marginalised"[Title/Abstract] OR "Marginalized"[Title/Abstract] OR "Refugee"[Title/Abstract] OR "Homeless"[Title/Abstract] OR "Migrant"[Title/Abstract] OR "financial catastrophe"[Title/Abstract] OR "poverty"[Title/Abstract] OR "conflict-affected"[Title/Abstract] OR ("Under"[All Fields] OR "attack"[Title/Abstract]) OR "Displaced"[Title/Abstract] OR "temporary accommodation"[Title/Abstract] OR "temporary settlement"[Title/Abstract] OR "temporary housing"[Title/Abstract] OR (("transit"[All Fields] OR "transited"[All Fields] OR "transiting"[All Fields] OR "transition"[All Fields] OR "Transitional"[All Fields] OR "transitionals"[All Fields] OR "transitioned"[All Fields] OR "transitioning"[All Fields] OR "transitions"[All Fields] OR "transits"[All Fields]) OR "Settlement"[Title/Abstract]) OR "transitional shelter"[Title/Abstract] OR "emergency shelter"[Title/Abstract] OR "emergency accommodation"[Title/Abstract] OR "emergency housing"[Title/Abstract] OR ("Makeshift"[All Fields] OR "shelter"[Title/Abstract]) OR ("Makeshift"[All Fields] OR "Accommodation"[Title/Abstract]) OR ("Slash"[Title/Abstract] OR "burn cultivation"[Title/Abstract]) OR "shifting cultivation"[Title/Abstract] OR "Feral"[Title/Abstract]	2,716,400	2:24:36

7	(((Child*[Title/Abstract]) OR (((Under five[Title/Abstract]) OR (Under 5[Title/Abstract])) OR (U5[Title/Abstract]))) OR (((Newborn[Title/Abstract]) OR (Infant[Title/Abstract])) OR (1000 days[Title/Abstract]))) AND (((Intervention*[Title/Abstract]) OR (Strateg*[Title/Abstract])) OR (Practice*[Title/Abstract]))			("child*"[Title/Abstract] OR ("under five"[Title/Abstract] OR "under 5"[Title/Abstract] OR "U5"[Title/Abstract]) OR ("Newborn"[Title/Abstract] OR "Infant"[Title/Abstract] OR "1000 days"[Title/Abstract])) AND ("intervention*"[Title/Abstract] OR "strateg*"[Title/Abstract] OR "practice*"[Title/Abstract])	262,165	2:12:15
6	((Intervention*[Title/Abstract]) OR (Strateg*[Title/Abstract])) OR (Practice*[Title/Abstract])			"intervention*"[Title/Abstract] OR "strategy*"[Title/Abstract] OR "practice*"[Title/Abstract]	2,553,193	2:11:54
5	((Child*[Title/Abstract]) OR (((Under five[Title/Abstract]) OR (Under 5[Title/Abstract])) OR (U5[Title/Abstract]))) OR (((Newborn[Title/Abstract]) OR (Infant[Title/Abstract])) OR (1000 days[Title/Abstract]))			"child*"[Title/Abstract] OR "under five"[Title/Abstract] OR "under 5"[Title/Abstract] OR "U5"[Title/Abstract] OR "Newborn"[Title/Abstract] OR "Infant"[Title/Abstract] OR "1000 days"[Title/Abstract]	1,806,063	2:10:53
4	((Newborn[Title/Abstract]) OR (Infant[Title/Abstract])) OR (1000 days[Title/Abstract])			"Newborn"[Title/Abstract] OR "Infant"[Title/Abstract] OR "1000 days"[Title/Abstract]	350,613	2:10:40
3	(Child*[Title/Abstract]) OR (((Under five[Title/Abstract]) OR (Under 5[Title/Abstract])) OR (U5[Title/Abstract]))			"child*"[Title/Abstract] OR "under five"[Title/Abstract] OR "under 5"[Title/Abstract] OR "U5"[Title/Abstract]	1,559,369	2:09:49
2	((Under five[Title/Abstract]) OR (Under 5[Title/Abstract])) OR (U5[Title/Abstract])			"under five"[Title/Abstract] OR "under 5"[Title/Abstract] OR "U5"[Title/Abstract]	16,327	2:09:27
1	Child*[Title/Abstract]			"child*"[Title/Abstract]	1,554,019	2:09:17

For peer review only

Data Extraction Form

(i) Citation Details

(ii) Research Design

(iii) Target Population

a. Sample Size

b. Inclusion

c. Number of participants
d. Number of sites
e. Type of Setting
f. Exclusion criteria
g. Demographics
    i. Age
    ii. Gender

(iv) Target Geography

(v) Intervention
a. Duration
b. Number of groups
c. Channels of Delivery
d. Agency for Delivery
e. Service Points
f. End-Users
g. Scale
h. Other Details

(vi) Evaluation
a. Type of Evaluation
b. Outcome Measures
    i. Primary
        1. ΔCoverage
        2. ΔAccess
        3. ΔUtilization
    ii. Secondary
c. Impact Measures
    i. Health
        1. ΔMorbidity
        2. ΔMortality
        3. ΔDALY
        4. ΔQALI
    ii. Economic
        1. RoI

For peer review only

2. ΔEfficiency
3. ΔCost

REFERENCES

CUSICK, S. E. & GEORGIEFF, M. K. 2016. The Role of Nutrition in Brain Development: The Golden Opportunity of the "First 1000 Days". *J Pediatr,* 175**,** 16-21.

LEVAC, D., COLQUHOUN, H. & O'BRIEN, K. K. 2010. Scoping studies: advancing the methodology. *Implementation science,* 5**,** 1-9.

MILLER, P. M. 2009. An Examination of the McKinney-Vento Act and Its Influence on the Homeless Education Situation. *Educational Policy,* 25**,** 424-450.

ROSENTHAL DM, L. C., HEYS M, SCHOENTHALER AM, UCCI M, HAYWARD A, ET AL. 2021. Barriers to Optimal Health for Under 5s Experiencing Homelessness and Living In Temporary Accommodation in High-Income Countries: A Scoping Review. *Ann Public Health Res,* 8(1):**,** 1103.

ROSENTHAL, D. M., UCCI, M., HEYS, M., HAYWARD, A. & LAKHANPAUL, M. 2020. Impacts of COVID-19 on vulnerable children in temporary accommodation in the UK. *The Lancet Public Health,* 5**,** e241-e242.

For peer review only

Preferred Reporting Items for Systematic reviews and Meta-Analyses extension for Scoping Reviews (PRISMA-ScR) Checklist

SECTION	ITEM	PRISMA-ScR CHECKLIST ITEM	REPORTED ON PAGE #
**TITLE**			
Title	1	Identify the report as a scoping review.	Page 1
**ABSTRACT**			
Structured summary	2	Provide a structured summary that includes (as applicable): background, objectives, eligibility criteria, sources of evidence, charting methods, results, and conclusions that relate to the review questions and objectives.	Page 2
**INTRODUCTION**			
Rationale	3	Describe the rationale for the review in the context of what is already known. Explain why the review questions/objectives lend themselves to a scoping review approach.	Page 3/4
Objectives	4	Provide an explicit statement of the questions and objectives being addressed with reference to their key elements (e.g., population or participants, concepts, and context) or other relevant key elements used to conceptualize the review questions and/or objectives.	Page 4
**METHODS**			
Protocol and registration	5	Indicate whether a review protocol exists; state if and where it can be accessed (e.g., a Web address); and if available, provide registration information, including the registration number.	Page 5
Eligibility criteria	6	Specify characteristics of the sources of evidence used as eligibility criteria (e.g., years considered, language, and publication status), and provide a rationale.	Page 5
Information sources*	7	Describe all information sources in the search (e.g., databases with dates of coverage and contact with authors to identify additional sources), as well as the date the most recent search was executed.	Page 6
Search	8	Present the full electronic search strategy for at least 1 database, including any limits used, such that it could be repeated.	Page 6
Selection of sources of evidence†	9	State the process for selecting sources of evidence (i.e., screening and eligibility) included in the scoping review.	Page 6
Data charting process‡	10	Describe the methods of charting data from the included sources of evidence (e.g., calibrated forms or forms that have been tested by the team before their use, and whether data charting was done independently or in duplicate) and any processes for obtaining and confirming data from investigators.	Page 7
Data items	11	List and define all variables for which data were sought and any assumptions and simplifications made.	Page 7
Critical appraisal of individual sources of evidence§	12	If done, provide a rationale for conducting a critical appraisal of included sources of evidence; describe the methods used and how this information was used in any data synthesis (if appropriate).	Page 7

St. Michael's
Inspired Care.
Inspiring Science.

For peer review only - http://bmjopen.bmj.com/site/about/guidelines.xhtml

SECTION	ITEM	PRISMA-ScR CHECKLIST ITEM	REPORTED ON PAGE #
Synthesis of results	13	Describe the methods of handling and summarizing the data that were charted.	Page 7
**RESULTS**			
Selection of sources of evidence	14	Give numbers of sources of evidence screened, assessed for eligibility, and included in the review, with reasons for exclusions at each stage, ideally using a flow diagram.	Page 8
Characteristics of sources of evidence	15	For each source of evidence, present characteristics for which data were charted and provide the citations.	Page 9-12
Critical appraisal within sources of evidence	16	If done, present data on critical appraisal of included sources of evidence (see item 12).	Page 13
Results of individual sources of evidence	17	For each included source of evidence, present the relevant data that were charted that relate to the review questions and objectives.	Page 9-12
Synthesis of results	18	Summarize and/or present the charting results as they relate to the review questions and objectives.	Page 13-15
**DISCUSSION**			
Summary of evidence	19	Summarize the main results (including an overview of concepts, themes, and types of evidence available), link to the review questions and objectives, and consider the relevance to key groups.	Page 16-18
Limitations	20	Discuss the limitations of the scoping review process.	Page 18
Conclusions	21	Provide a general interpretation of the results with respect to the review questions and objectives, as well as potential implications and/or next steps.	Page 18-19
**FUNDING**			
Funding	22	Describe sources of funding for the included sources of evidence, as well as sources of funding for the scoping review. Describe the role of the funders of the scoping review.	Page 19

JBI = Joanna Briggs Institute; PRISMA-ScR = Preferred Reporting Items for Systematic reviews and Meta-Analyses extension for Scoping Reviews.

* Where *sources of evidence* (see second footnote) are compiled from, such as bibliographic databases, social media platforms, and Web sites.

† A more inclusive/heterogeneous term used to account for the different types of evidence or data sources (e.g., quantitative and/or qualitative research, expert opinion, and policy documents) that may be eligible in a scoping review as opposed to only studies. This is not to be confused with *information sources* (see first footnote).

‡ The frameworks by Arksey and O'Malley (6) and Levac and colleagues (7) and the JBI guidance (4, 5) refer to the process of data extraction in a scoping review as data charting.

§ The process of systematically examining research evidence to assess its validity, results, and relevance before using it to inform a decision. This term is used for items 12 and 19 instead of "risk of bias" (which is more applicable to systematic reviews of interventions) to include and acknowledge the various sources of evidence that may be used in a scoping review (e.g., quantitative and/or qualitative research, expert opinion, and policy document).

From: Tricco AC, Lillie E, Zarin W, O'Brien KK, Colquhoun H, Levac D, et al. PRISMA Extension for Scoping Reviews (PRISMAScR): Checklist and Explanation. Ann Intern Med. 2018;169:467–473. doi: 10.7326/M18-0850.
